# High Reactivity of Dimethyl Ether Activated by Zeolite Ferrierite within a Fer Cage: A Prediction Study

**DOI:** 10.3390/molecules29092000

**Published:** 2024-04-26

**Authors:** Xiaofang Chen, Pei Feng, Xiujie Li

**Affiliations:** 1 Institute of Frontier Chemistry, School of Chemistry and Chemical Engineering, Shandong University, Qingdao 266237, China; 2 State Key Laboratory of Molecular Reaction Dynamics, Dalian Institute of Chemical Physics, Chinese Academy of Sciences, Dalian 116023, China; fengpei@stdu.edu.cn; 3 State Key Laboratory of Catalysis, Dalian Institute of Chemical Physics, Chinese Academy of Sciences, Dalian 116023, China

**Keywords:** zeolite, dimethyl ether, carbonylation, ferrierite, oxonium ion, periodic density functional theory (PDFT), periodic boundary structure, barrier, rate constant

## Abstract

The zeolite-catalyzed conversion of DME into chemicals is considered environmentally friendly in industry. The periodic density functional theory, statistical thermodynamics, and the transition state theory are used to study some possible parallel reactions about the hydrogen-bonded DME over zeolite ferrierite. The following are the key findings: (1) the charge separation probably leads to the conversion of a hydrogen-bonded DME into a dimethyl oxonium ion (i.e., DMO^+^ or (CH_3_)_2_OH^+^) with a positive charge of about 0.804 e; (2) the methylation of DME, CH_3_OH, H_2_O, and CO by DMO^+^ at the T2O6 site of zeolite ferrierite shows the different activated internal energy (∆E^≠^) ranging from 18.47 to 30.06 kcal/mol, implying the strong methylation ability of DMO^+^; (3) H-abstraction by DMO^+^ is about 3.94–15.53 or 6.57–18.16 kcal/mol higher than DMO^+^ methylation in the activation internal energy; (4) six DMO^+^-mediated reactions are more likely to occur due to the lower barriers, compared to the experimental barrier (i.e., 39.87 kcal/mol) for methyl acetate synthesis; (5) active intermediates, such as (CH_3_)_3_O^+^, (CH_3_)_2_OH^+^, CH_3_CO^+^, CH_3_OH_2_^+^, and CH_2_=OH^+^, are expected to appear; (6) DMO^+^ is slightly weaker than the well-known surface methoxy species (ZO-CH_3_) in methylation; and (7) the methylated activity declines in the order of DME, CH_3_OH, H_2_O, and CO, with corresponding rate constants at 463.15 K of about 3.4 × 10^4^, 1.1 × 10^2^, 0.18, and 8.2 × 10^−2^ s^−1^, respectively.

## 1. Introduction

Acidic zeolites have been widely used to catalyze the conversion of dimethyl ether (DME) or methanol into valuable chemicals (e.g., olefins, methyl acids, and methyl acetates (MAs)) over the past several decades. In 2006–2007, the Iglesia group [1,2,3] reported that acidic zeolites like mordenite and ferrierite, which serve as halide-free catalysts, have the ability to accelerate DME carbonylation. They found that the selectivity towards methyl acetate was particularly high, exceeding 99% at optimized experiment temperatures ranging from 423 K to 463 K. The enduring catalytic activity of those zeolites could be retained for a long time with the progress of chemical reactions, and there is less coke formation during DME carbonylation [1,2,3,4,5]. The outstanding catalytic performances and high product selectivity have received increasing attention in the field of both academy and industry [6,7,8,9,10,11,12,13,14,15,16,17,18,19,20,21,22,23,24].

The crystal structures of zeolites are of importance. Zeolite ferrierite is the rare aluminosilicate mineral [25]. In terms of X-ray powder data, the crystalline ferrierite was previously identified as the porous material with the space group of Immm and two obvious intersecting channels [26,27,28]. The high symmetry of the space group reduces the number of structural non-equivalent atoms, with one unit cell (UC) of pure-silica FER framework containing eight non-equivalent oxygen atoms and four non-equivalent tetrahedral (T) atoms. Later, the crystal structure of ferrierite was re-determined by the synchrotron X-ray and the neutron powder diffraction experiment, in which the Si-O-Si angle was proved to be ~170° rather than 180° [29]. The favored Al substitution sites were suggested to be at the T2 or T4 site of the acidic zeolite ferrierite [30,31,32]. Two Brönsted acid (BA) protons at O4 (shared by T1 and T3 cations, pointing to a fer cage) and O6 (shared by two T4 cations) sites of the low-silica ferrierite were recognized by the neutron experiment [33].

The catalytic behavior of zeolites is nearly determined by the confinement environment around zeolitic Brönsted acid sites, including factors such as the adsorption location, the proton distribution and siting, the acid strength, and the reaction center [11,12,34,35,36,37,38,39]. Low or undetectable amounts of carbonylation species were evidenced in experiments where certain zeolites lacked eight-membered ring (8MR) channels (e.g., H-BEA, H-FAU, and H-MFI) [1,2,3]. The eight-member ring channels in zeolites mordenite and ferrierite were suggested as the specific zones for CO insertion into chemisorbed species during DME carbonylation [3,6,7,13,32,40,41]. The shift in hydroxyl vibrational frequency indicated that CO, N_2_, and H_2_ preferred a 10-membered ring channel over a fer cage of zeolite ferrierite [42]. Additionally, the interaction of CO with silanol groups was also evidenced at low temperatures, with the carbonyl adsorbed species varying with the temperature [43]. At the reaction temperature, CO and DME molecules assumedly preferred the 8-membered ring channel of zeolite ferrierite [12]. It was found that smaller alkanes than n-pentane could access the entire pore of ferrierite, while longer alkanes preferred the 10-membered ring to the 8-membered ring cage [44]. T2O6 configuration was suggested to be the strongest BA site of the acidic zeolite ferrierite from the statistical perspective [13]. Experimental and theoretical evidence indicated that small molecules like methanol and DME were mainly accumulated in the 8- and 6-member ring channels of zeolite ferrierite [1,2,3,6,13,32,40].

Based on the potential active intermediate, many researchers have attempted to interpret the fast kinetic process of DME carbonylation. The Iglesia group [1,2,3] proposed that the surface methoxy species (i.e., SMS or ZO-CH_3_) was a key intermediate with high activity, which served as a catalyst to trigger and propagate the elemental chemical reactions during DME carbonylation. They also presented a potential catalytic circle grounded on ZO-CH_3_. The high reactivity of ZO-CH_3_ was theoretically confirmed by other researchers [6,7,13]. Apart from ZO-CH_3_, three species of CH_3_CO^+^, CH_3_OH, and H_2_O were also suggested to be other possible intermediates by the Iglesia group [1,2,3,16]. Later, Jensen’s group [45] theoretically predicted ketene (CH_2_CO) as a possible reaction intermediate for DME carbonylation, and they further verified the existence of ketene by doubly deuterated acetic acid (CH_2_DCOOD) in the experiment. Meanwhile, the hydrogen-bonded DME could coexist with ZO-CH_3_, which is frequently detected by the IR spectra experiment with the peaks at 3011, 2971, 2947, and 2844 cm^−1^ [1,2,46]. This hydrogen-bonded DME was initially formed by DME reacting with the proton (H^+^) of the acidic zeolite; sometimes, it was regarded as the protonated DME or DME adsorption at the Brönsted acid site [1,2,3]. The BA proton has a strong tendency to draw the electron density of the electron-rich O atom and then changes the electrostatic potential of the molecule of DME. DME moiety in the adsorption complex is readily activated by acidic zeolite. Even now, it is a relatively unexplored area of research on the chemical role of the hydrogen-bonded DME over the acidic zeolites. 

In this study, we design six possible chemical reactions (Equations (1)–(6)) between the attacking molecules and the hydrogen-bonded DME at the T2O6 site of zeolite ferrierite. The attacking molecules are two feed molecules (i.e., DME and CO) and two key intermediates (i.e., CH_3_OH and H_2_O) from DME carbonylation. Equations (1)–(4) are the hydrogen-bonded DME methylating with four attacking molecules (i.e., DME, CH_3_OH, H_2_O and CO), respectively. Equation (5) or (6) is the hydrogen-bonded DME abstracting one of the methyl H atoms of DME or CH_3_OH. Zeolite ferrierite is a porous material with a three-dimensional network topology. We employ the period density functional theory (PDFT), statistical thermodynamics, and transition state theory to predict the chemical kinetics and the reaction mechanism of those six chemical reactions. The aim of this study is to provide some theoretical evidence to identify the hydrogen-bonded DME as the highly active intermediate from different perspectives.

- Methylation of hydrogen-bonded DME within the deprotonated zeolite (ZO^−^):
  (CH_3_)_2_OH^+^ + CH_3_OCH_3_ → (CH_3_)_3_O^+^ (i.e., TMO^+^) + CH_3_OH (1)
  (CH_3_)_2_OH^+^ + CH_3_OH → CH_3_OH + (CH_3_)_2_OH^+^ (2)
  (CH_3_)_2_OH^+^ + H_2_O → CH_3_OH + CH_3_OH_2_^+^ (3)
  (CH_3_)_2_OH^+^ + CO → CH_3_CO^+^ + CH_3_OH (4)
- H-abstraction of hydrogen-bonded DME within ZO^-^:
  (CH_3_)_2_OH^+^ + CH_3_OCH_3_ → CH_3_OH + CH_4_ + CH_3_OCH_2_^+^ (5)
  (CH_3_)_2_OH^+^ + CH_3_OH → CH_3_OH + CH_4_ + CH_2_OH^+^ (6)

## 2. Results and Discussion

The acidic zeolite ferrierite features an FER framework containing eight non-equivalent oxygen atoms and four non-equivalent tetrahedral (T) atoms in one unit cell [26,27,28]. The T2O6 site was identified as the strongest acid site of the acidic zeolite ferrierite from a statistical perspective [11]. In this context, we focus on the reactivity of the hydrogen-bonded DME only at the T2O6 site of the acidic zeolite ferrierite. One of the attacking molecules and one DME molecule are all put in the fer cage of the acidic zeolite ferrierite. The attacking molecules influence on the reactivity of DMO^+^ at the T2O6 site will be addressed and discussed in the following sections. 

### 2.1. DME Adsorbed on the Zeolitic Surface

**Figure 1:**
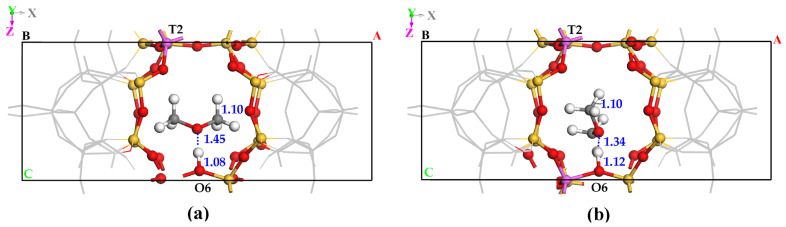
Dimethyl ester (DME) adsorbed at the T2O6 site of zeolite ferrierite within the eight-membered ring (8MR) channel: (**a**) parallel to and (**b**) perpendicular to the [010] plane. Noting that (1) O in red, Si in yellow, Al in pink, C in grey, and H in white; (2) grey lines for interconnecting atoms away from the 8MR, and (3) the blue number is the distance (in Å) between two atoms.

The experiment observed that DME pressure (0.8–8.0 kPa) did not determine the rate of methyl acetate synthesis [1,2] from DME carbonylation. This phenomenon was likely caused by the active sites saturated by DME-derived intermediates. The Brönsted acid proton is electron-deficient, while the O atom of DME has a lone electron pair. It is generally accepted that DME is first adsorbed end-on at the zeolitic Brönsted acid site via the hydrogen bond interaction. The 8MR channel as the specific zone of DME carbonylation was frequently emphasized in previous studies [3,6,13,32,40,41]. DME adsorbed at the T2O6 site would act as an ion–pair complex [47]. DME could be accumulated in the 8MR channel of the acidic zeolite ferrierite (H-FER). In the adsorption complex, DME is nearly parallel to Figure 1a or perpendicular to Figure 1b, the [010] plane of H-FER. As for the internal energy, the former lies above the latter by 3.25 kcal/mol. The pore size of the 8MR channel in zeolite ferrierite is 4.8 × 3.5 Å [28], while the molecule size of DME is generally regarded as 4.3–5.0 Å (Figure S1 in Supplementary Materials). When a DME molecule is perpendicular to the [010] plane, it would be better to overcome the space hindrance. This might contribute to accounting for the space preference of DME moiety in the adsorption complex. We only consider DME perpendicular to the [010] plane of H-FER in DME adsorption in the following sections. The Brönsted acid H atom is calculated to be 0.98 Å away from the zeolitic framework O6 atom of the undisturbed H-FER. The BA hydroxyl bond length is elongated to 1.12 Å in the presence of DME. The electron density transfers to the O6 atom from the Brönsted acid H atom, making the H···O(CH_3_)_2_ moiety exhibit the positive charge, and the Bader charge analysis indicates that H···O(CH_3_)_2_ moiety has the positive charge of about 0.804 e. The moiety of H···O(CH_3_)_2_ in the adsorption complex tends to become a somewhat dimethyl oxonium ion [48] (i.e., (CH_3_)_2_OH^+^ or DMO^+^) via the charge separation. For convenience, DMO^+^ is used to represent the hydrogen-bonded DME in the following sections.

### 2.2. Attacking Molecule Serving as Electron Donor

**Scheme 1:**
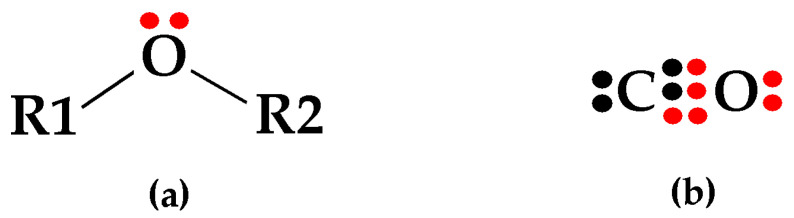
Lone electrons in (**a**) DME, CH_3_OH, and H_2_O, and (**b**) CO. Notes: (1) for DME, R1 = R2 = -CH_3_; (2) for CH_3_OH, R1 = CH_3_, R2 = H; (3) for H_2_O, R1 = R2 = H; (4) a red or black dot represents an electron from the O or C atom.

In the context of DME carbonylation, DME and CO serve as main feed materials, while CH_3_OH and H_2_O are suggested to be two key intermediates of DME carbonylation [1,2,13]. Each molecule of DME, CH_3_OH, or H_2_O has two lone electrons stationed at the O atom (Scheme 1a). A CO molecule has a triple covalent C≡O bond originating from three pairs of shared double electrons: two pairs are from C and O atoms, while the third pair is completely from the O atom and then enters an unoccupied *p*-orbital of the C atom (Scheme 1b). Those four molecules all have the lone electron pair. DME, CH_3_OH, H_2_O, or CO belongs to the electron-rich molecule. Consequently, each molecules is expected to have the ability to act as an electron donor.

### 2.3. Methylation Process of Hydrogen-Bonded DME (i.e., DMO^+^)

#### 2.3.1. Reaction Course of DMO^+^ Methylation

**Figure 2:**
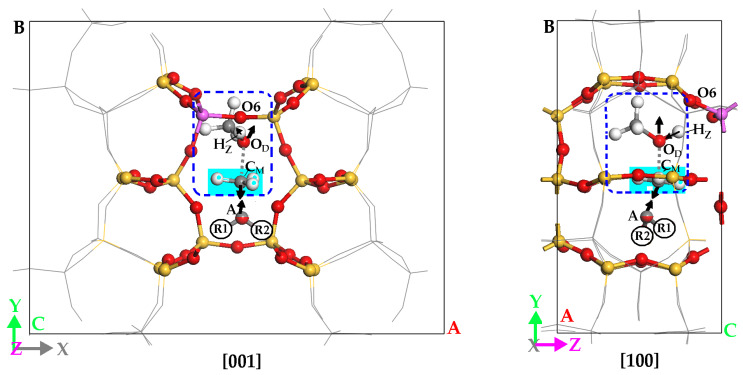
Within a fer cage of zeolite ferrierite, an attacking molecule (R1-A-R2) is methylated by a hydrogen-bonded DME molecule (i.e., DMO^+^) at the T2O6 site (in a blue dash square). Notes: (1) O in red, Si in yellow, Al in pink, C in grey, and H in white, and grey lines for interconnecting atoms and bonds away from the fer cage; (2) DME: A = O, R1 = R2 = CH_3_; (3) CH_3_OH: A = O, R1 = CH_3_, R2 = H; (4) H_2_O: A = O, R1 = R2 = H; (5) CO: A = C, R1 = O, and R2 is omitted; (6) H_Z_, O_D_, C_M_, and A are the Brönsted acid proton, O atom of DME, the migrant C atom, and target A atom, and (7) the black arrow is the main direction of atom movement.

DME, CH_3_OH, H_2_O, and CO are assumedly abundant in DME carbonylation. The O atom of DME, CH_3_OH, or H_2_O and the C atom of CO are defined as the target A atom in this study. When DME is adsorbed at the T2O6 site of the acidic zeolite ferrierite in the presence of an attacking molecule (R1-O-R2), we could put an attacking molecule into a fer cage along the eight-membered ring channel of the acidic zeolite ferrierite (Figure 2). As shown in Figure 2, the target A atom would be most close to one of the methyl groups of the hydrogen-bonded DME. In the reactant systems of Re1-Re4, the BA hydroxyl bond length (d(O6-Hz)) is stretched to 1.31, 1.28, 1.28, and 1.15 Å, respectively, already elongated relative to that (0.98 Å) in the undisturbed H-FER. Meanwhile, the BA proton further approaches the O atom of DME, and the moiety of H···O(CH_3_)_2_ is with the larger positive charge. The Bader charge analysis indicates that the charge of H···O(CH_3_)_2_ moiety in Re1-Re4 is about 0.790 e, 0.919 e, 0.945 e and 0.739 e, respectively. In turn, the transmethylation could occur between the electron-deficient DMO^+^ and the electron-rich attacking molecule via the S_N_2-like [49] transition state (i.e., TS1-TS4) (Figure 2), in which three atoms, O_D_, C_M_, and target A atom in each transition state, are almost in line with the ∠O_D_C_M_A angle ranging from 168.85° to 178.54°. Herein, H_Z_, C_M_, O_D,_ and A are the BA proton, the migrant C atom, the O atom of the activated DME, and the target A atom. The migrant methyl group becomes nearly planar (∠C_M_HHH below 7.86°) from the umbrella undisturbed DMO^+^. For the structures and the coordinates of reactants and transition states, please refer to Supplementary Materials.

The calculated imaginary frequency is 398.94*i*, 389.71*i*, 381.85*i*, or 542.61*i,* responsible for the bond breakage and the bond formation when DMO^+^ methylating with DME, CH_3_OH, H_2_O, or CO. The black arrows in Figure 2 show the direction of atom movement of interest. As shown, the will-be-broken chemical bonds involve the BA hydroxyl bond (i.e., O6-H_Z_) of H-FER and the C-O bond of DME, while two new bonds would be formed between (i) the acidic zeolite BA proton and the O atom of DME, and (ii) between the migrant methyl group of the hydrogen-bonded DME and target A atom of the attacking molecule (R1-A-R2). Along the reaction course of DMO^+^ methylation, the BA proton gradually moves away from the zeolitic framework O6 atom and scrambles to the O atom of DME; the migrant methyl group (-C_M_H_3_) gradually scrambles to the target atom (A) of the attacking molecule.

**Figure 3:**
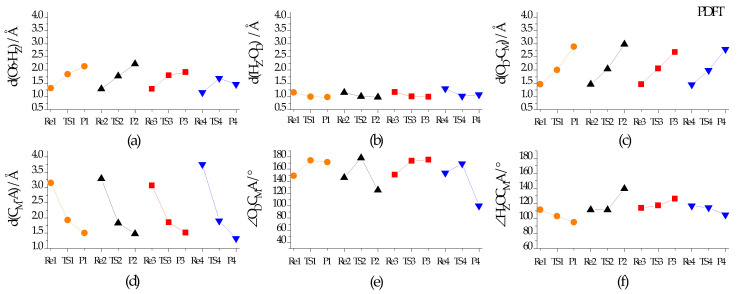
Some distances (**d**) and angles (∠) in the reactant (Rex), the transition state (TSx), and the product (Px) of Equation (x) (x = 1–4) in the Section 1: (**a**) d(O6-H_Z_), (**b**) d(H_Z_-O_D_), (**c**) d(O_D_-C_M_), (**d**) d(C_M_-A), (**e**) ∠O_D_C_M_A, and (**f**) ∠H_Z_OC_M_A. Notes: (1) O6, H_Z_, O_D_, and C_M_ are the framework O6 atom and the BA proton of zeolite ferrierite, and the O atom and the migrant C atom of the hydrogen-bonded DME (i.e., DMO^+^); (2) A is the O atom of DME, CH_3_OH or H_2_O moiety, yet it is the C atom of CO moiety; (3) Each color represents the species in one chemical equation.

Figure 3 further shows the numerical change of structure parameters of interest. Along the reaction course of reactant (Rex) → transition state (TSx) → product (Px) (x = 1–4), as calculated, (i) either the BA hydroxyl distance (i.e., d(O6-H_Z_)) (Figure 3a) or the distance between the BA proton and O atom of DME moiety (i.e., d(O_D_-C_M_)) (Figure 3c) generally grows; (ii) either the distance between the BA proton and the O atom of DME (i.e., d(Hz-O_D_)) (Figure 3b) or the distance between the migrant C atom (C_M_) of DMO^+^ and the target atom (A) of the attacking molecule (i.e., d(C_M_-A)) (Figure 3d) declines gradually; (iii) three atoms O_D_, C_M_, and A (Figure 3e), oscillate and their angle (i.e., ∠O_D_C_M_A) wanders around 180°; but (iv) the dihedral angle of H_Z_OC_M_A (i.e., ∠H_Z_OC_M_A) changes and yet does not have the same trend. Taking TS4 for an example, d(O6-H_Z_) in TS4 is stretched to 1.70 Å from 1.12 Å in DME adsorption complex (Figure 1b) while d(H_Z_-O_D_) in TS4 is shrunk to 1.01 Å from 1.34 Å in the DME adsorption complex (Figure 1b); d(C_M_-A) in TS4 becomes 1.91 Å from 3.76 Å in Re4; ∠O_D_C_M_A in TS4 is equal to 168.85°, and ∠H_Z_OC_M_A equals to 153.81°.

#### 2.3.2. Energy Parameters and Products for DMO^+^ Methylation

**Figure 4:**
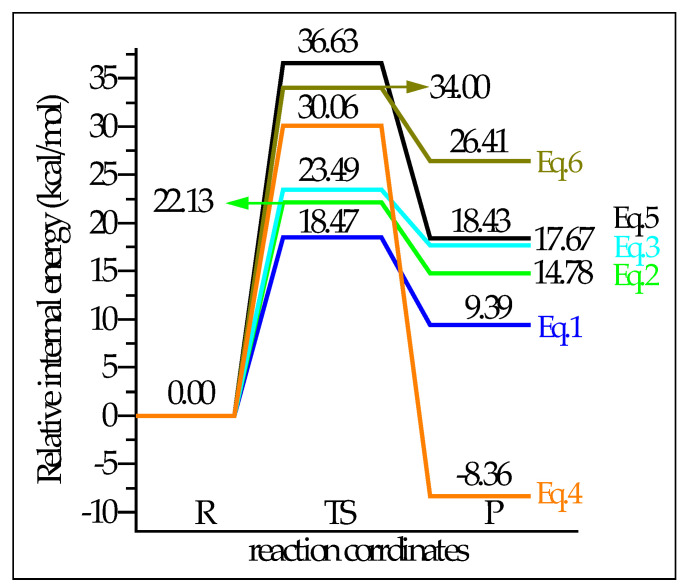
Calculated energy profile for DMO^+^ methylation and H-abstraction at T2O6 site of zeolite ferrierite within a fer cage. For Equations (1)–(6), please refer to the Section 1.

Figure 4 shows that the activated internal energies (∆E^≠^) for those four methylation reactions (Equations (1)–(4)) are all below 30.06 kcal/mol when DMO^+^ is located at the T2O6 site, and the attacking molecule is within the fer cage of zeolite ferrierite. This implies that those transmethylations readily proceed rapidly in kinetics. Among the four attacking molecules, the DME molecule needs to overcome the lowest activated internal energy (~18.47 kcal/mol) after adsorbing a lower reaction heat of about 9.39 kcal/mol. In contrast, CO requires surmounting the highest activated internal energy (~30.06 kcal/mol) despite releasing a low reaction heat of approximately −8.36 kcal/mol. Thus, the methylation of DME by DMO^+^ would be kinetically favored, while the methylation of CO by DMO^+^ would be thermodynamically favored at the T2O6 site of zeolite ferrierite. The methylation of DME by DMO^+^ yields a contentious active species called trimethyloxonium ion (TMO^+^ or (CH_3_)_3_O^+^), which plays a crucial role in the first C-C bond formation in the DME/methanol-to-olefin (DMTO) via the direct mechanism [2,6,13,50,51,52]. When methylating with CO, DMO^+^ might be regarded as an efficient precursor “seed” of the carbon chain growth for C1 species (Equation (4)), firstly generating CH_3_CO^+^ and then likely leading to CH_3_COCH_3_ [1,2]. CH_3_OH methylated by DMO^+^ is an ion exchange reaction in which the reactant species are the same as the product species. Its activated internal energy is 22.13 kcal/mol, close to a theoretical ∆E^≠^ (i.e., 19.9 kcal/mol) for CH_3_OH methylated by the most stable ZO-CH_3_ on MOR [6]. DMO^+^ methylating with H_2_O will produce a highly active intermediate of CH_3_OH_2_^+^, called a methoxonium ion (Equation (3)) [53]. The activated internal energy for DMO^+^ methylating with water is about 6.57 kcal/mol less than that for DMO^+^ methylating with CO. DMO^+^ preference of H_2_O over CO also contributes to accounting for the experimental phenomenon that a small amount of water inhibits DME carbonylation [1,2]. Apart from CO, DME, CH_3_OH, and H_2_O methylated by DMO^+^ are all endothermic. The least endothermic methylation process (i.e., 9.39 kcal/mol) is from DME, followed by CH_3_OH. The reaction heat would be easily supplied by DME carbonylation under the specific condition. To complete the methylation process, two attacking molecules of CH_3_OH and H_2_O require absorbing the higher reaction internal energy (about 14.78 and 17.67 kcal/mol, respectively). The magnitude of activated internal energy also implies that four attacking molecules exhibit differently methylated abilities; in kinetics, the order is assumedly DME > CH_3_OH > H_2_O > CO. It is worth noting that the calculated activated internal energies for those four methylation processes are all lower than the deduced experimental barrier (about 39.87 kcal/mol) for methyl acetate synthesis [1,2].

### 2.4. H-Abstract of Hydrogen-Bonded DME (i.e., DMO^+^)

#### 2.4.1. Reaction Course of DMO^+^ H-Abstraction

**Figure 5:**
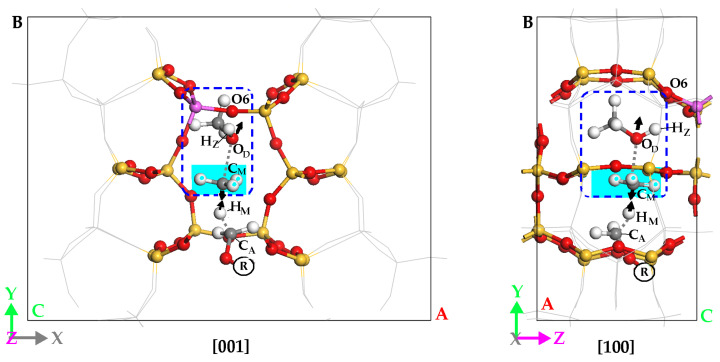
The hydrogen-bonded DME (in blue dash square) abstracts one of methyl H atoms of attracting molecules (i.e., DME or CH_3_OH) at the T2O6 site within a fer cage of zeolite ferrierite. Notes: (1) O in red, Si in yellow, Al in pink, C in grey, and H in white, grey lines for interconnecting atoms and bonds away from the fer cage; (2) for DME, R = -CH_3_; for CH_3_OH, R = H; (3) O_D_, C_M_, and H_M_ are the O atom and the migrant methyl C atom of hydrogen-bonded DME, and the migrant methyl H atom of attracking molecules, and (4) the black arrow is the main direction of atom movement of interest.

When DME is adsorbed at the T2O6 site, the attacking molecule of DME or CH_3_OH is located within a fer cage of the acidic zeolite ferrierite in another manner, where the methyl group of DME or CH_3_OH, rather than the O atom, is most close to the migrant methyl group of the activated DME (Figure 5). The Brönsted acid hydroxyl bond length (d(O6-Hz)) is calculated to be 1.12 or 1.14 Å in the reactant system of Re5 or Re6 (the structure is similar to that in Figure 5). It is slightly elongated by 0.14 or 0.16 Å relative to the undisturbed H-FER (i.e., d(O6-Hz) = 0.98 Å). When the Brönsted acid proton gradually approaches the O atom of DME, the partial electron density will move to the O atom of DME, resulting in the moiety of H···O(CH_3_)_2_ with the positive charge. The Bader charge analysis indicates that H···O(CH_3_)_2_ moiety in Re5 or Re6 has a positive charge of about 0.769 e or 0.764 e. This implies that the hydrogen-bonded DME in Re5 or Re6 is already activated by the Brönsted acid proton. The migrant methyl group of the hydrogen-bonded DME (i.e., DMO^+^) exhibits a weak carbenium ion, and the calculated Bader charge is about 0.580 e or 0.575 e. Within the deprotonated zeolite ferrierite, it is expected that the hydrogen-bonded DME (i.e., DMO^+^) will abstract one of the methyl H atoms of DME or CH_3_OH. 

Two transition states, TS5 and TS6 are located (the structure is similar to Figure 5), which are responsible for DMO^+^ abstracting the methyl H atom of DME and CH_3_OH, respectively. The migrant atoms in transition states are mainly (i) Brönsted acid proton (H_Z_), (ii) the O atom (O_D_) of the hydrogen-bonded DME, (iii) the C atom (C_M_) of the migrant methyl of the hydrogen-bonded DME, (iv) the migrant methyl H atom (H_M_) of DME or CH_3_OH, and (v) the C atom (C_A_) of the attacking molecule DME or CH_3_OH. For the direction of atom movement of interest, please see the black arrows in Figure 5. Then, the reaction course involves three main parts: the nearly linear O6···H_Z_···O_D_ (163.37° or 165.32°), the nearly linear O_D_···C_M_···H_M_ (173.76° or 173.37°), and the bent C_M_···H_M_···C_A_ (138.61° or 144.24°). The distance (d(O6-H_Z_)) is stretched to 1.82 or 1.77Å from 0.98 Å in the undisturbed H-FER, implying that the BA proton in TS5 or TS6 is gradually away from the zeolitic surface oxygen. The O_D_···C_M_ distance in TS5 or TS6 is stretched to 2.13 Å, then DMO^+^ moiety would undergo the breakage of the O_D_-C_M_ bond. The H_M_···C_A_ distance in the transition state increases to 1.25 Å from 1.10 Å in the methyl group of DME or CH_3_OH, resulting in the breakage of methyl C-H bond of the attacking molecule. The new chemical bond will be formed between the migrant methyl C atom (C_M_) of the reacting DMO^+^ and the methyl H atom of the attacking molecule, and the C_M_···H_M_ distance in the transition state is largely shortened to 1.46 Å or 1.40 Å from 3.36 Å or 2.96 Å in the reacting system. The migrant methyl group in TS5 or TS6 is almost in the format of a carbenium ion, and then it becomes nearly planar in order to better interact with the O_D_ and H_M_ atoms. For the structural details, refer to Figure S4 and Table S2 in Supplementary Materials.

#### 2.4.2. Energy Parameters and Products for DMO^+^ H-Abstraction

When DMO^+^ H-abstraction proceeds, (i) methane (CH_4_) would be formed by the nearly planar CH_3_^+^ interacting with the methyl H atom, (ii) CH_3_OH would be produced by the BA proton transfer and the O-C bond breakage of DMO^+^, and (iii) the attacking molecule of DME or CH_3_OH becomes CH_3_OCH_2_^+^ or the protonated formaldehyde (CH_2_OH^+^) by losing one of methyl H atoms. Whether the attacking molecule is CH_3_OH, the H-abstract of DMO^+^ within zeolite ferrierite probably undergoes a so-called methane-formaldehyde mechanism, similar to some reactions in methanol-to-olefins [54,55,56]. The active intermediate CH_3_OCH_2_^+^ is capable of being further methylated by the methylating agent, producing dimethyloxoniummethylide, [(CH_3_)_2_-O^+^CH_2_^−^]. This might make DME carbonylation occur via an oxonium ion or oxonium-ylide mechanism, which is frequently mentioned in MTO [48,50,51,57,58]. Our results also indicate that, within a fer cage of zeolite ferrierite, each process of DMO^+^ H-abstraction is endothermic (i.e., 18.43 or 26.41 kcal/mol) and has a moderate activation internal energy of 36.63 or 34.00 kcal/mol. The activation internal energy of DMO^+^ H-abstraction is slightly bigger than that (i.e., 32.32 kcal/mol) for ZO-CH_3_ abstracting the methyl H atom of CH_3_OCH_3_ [54], and yet it is slightly smaller than the deduced barrier (about 39.87 kcal/mol) for methyl acetate synthesis in the experiment [1,2]. This implies that DMO^+^ H-abstraction is more likely to occur within a fer cage of zeolite ferrierite. In terms of the activated internal energy, DMO^+^ H-abstraction is kinetically weaker than DMO^+^ methylation (Section 2.3) or ZO-CH_3_ methylation [2,6,13]. The reason for this is probably because the C-H bond is stronger than the C-O bond. As a result, a very small number of CD_3_OCD_3_-CO mixtures were observed in methyl acetate synthesis in the experiment [1,2].

### 2.5. Rate Constant

It is found that DME carbonbylation is experimentally carried out at the optimized reaction temperature of 423–463 K [1,2,3,4,5]. At the higher temperature, the entropic effect is an important contribution to the Gibbs free energy. For the micro-heterogeneous catalysis reaction in the elemental step, the reaction belongs to the first-order reaction in terms of transition state theory (TST). The rate constant is related to the Gibbs free energy at a given temperature. The numerical values of the Gibbs free energy could be calculated according to Equations (7)–(11).

(7) $$k=\frac{k_{b}\cdot T}{h}exp\left(-\frac{∆{G(T)}^{\neq }}{R\cdot T}\right)$$

G(T) = H − T·S (8)

S = S_e_ + S_t_ + S_r_ + S_v_ (9)

(10) $$S={\left(\frac{\partial F}{\partial T}\right)}_{V,N}$$

F = −k_b_·T·lnq^N^ (11)

Herein, *k* is the first-order rate constant, *k_b_* is the Boltzmann constant, *h* is the Plank constant, *R* is the ideal gas constant, *T* is the temperature, and ∆*G*(*T*) is the activation free energy at a given temperature, which could be calculated by the Gibbs free energy (*G*(*T*)) difference between the transition state and the reactant system. *G*(*T*) is related to the enthalpy (*H*), the temperature (*T*), and the entropy (*S*) (Equation (8)). The entropy (*S*) of the material is the sum of the electric (*S_e_*), the transitional (*S_t_*), the vibrational (*S_v_*), and the rotational (*S_r_*) entropy (Equation (9)). The entropy stems from the derivative of Helmholtz free energy (*F*) divided by the temperature at the constant volume and the constant number of particles (Equation (10)). And the Helmholtz free energy (*F*) is related to the partition function (*q*) (Equation (11)). The temperature selected ranges from 273.15 to 773.15 K in this study.

**Figure 6:**
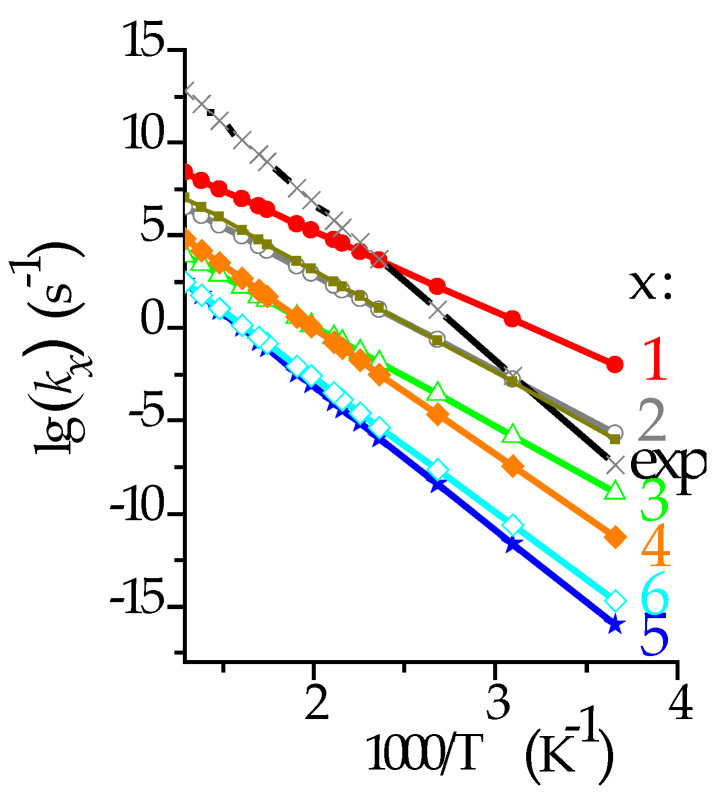
Rate constants (k_x_) for DMO^+^ methylation (x = 1–4) and DMO^+^ H-abstraction (x = 5–6), and the experimental synthesis of methyl acetate (k_exp_).

Figure 6 illustrates the rate constants dependent on the temperature. As calculated, the rate constants for DMO^+^ methylating with CO or H_2_O, as well as DMO^+^ abstracting the methyl H atom of DME or CH_3_OH, are below the deduced rate constant (the black line) for methyl acetate synthesis in the experiment [1,2]. Methylated by DMO^+^, water completes with CO heavily. As calculated, DMO^+^ assumedly prefers water to CO at low temperatures (about <522.60 K), and CH_3_CO^+^ formation is slower than CH_3_OH_2_^+^ formation by about 0–2.38 orders of magnitude. This could quantitatively account for water-inhibiting DME carbonylation at the optimized experiment temperatures of 423–463 K [1,2]. In the presence of attacking molecule DME or CH_3_OH, DMO^+^ H-abstraction is 9.23–8.89 or 6.11–5.90 orders of magnitude slower than the corresponding methylation process at 423.15–463.15 K. This is in line with the experimental observation of the small amount of CD_3_OCD_3_-CO mixtures (*k_H_*/*k_D_* = 1.06) appearing in DME carbonylation [2]. When H-abstracted by DMO^+^ at the T2O6 site within a fer cage, DME is slower than CH_3_OH by 0.50–0.56 orders of magnitude at 443.15–463.15 K. DME carbonylation tends to produce TMO^+^ intermediate in common features with the methanol-to-hydrocarbon process [50]. 

As calculated, the rate constants at 463.15 K for six designed reactions (Equations (1)–(6)) are about 3.4 × 10^4^, 1.1 × 10^2^, 0.18, 8.2 × 10^−2^, 4.4 × 10^−5^, and 1.4 ×10^−4^ s^−1^, respectively. Those numerical values of rate constants imply that DMO^+^ and TMO^+^ are likely to be produced in DME carbonylation. Both DMO^+^ and TMO^+^ act as sources of the methyl agent and are more likely to take part in the sequent CO insertion steps [1,2,6,7,16]. It is worth noting that the orientation of the attacking molecules and the stereoscopic effect will have some influences on the chemical reaction.

## 3. Materials and Methods

All calculations are carried out using the Vienna Ab initio Simulation Package (VASP) [59,60,61,62] implemented with the plane-wave periodic gradient-corrected density functional theory methods. The Perdew–Burke–Ernzerh (PBE) [63] pseudopotentials (version 52) are used to describe the exchange–correlation functional, and the projector augmented wave approximation (PAW) [64] of Blöchl is adopted to describe the electron-ion interaction. Standard PAW pseudopotentials are used in this study. The atoms involved are H, O, C, Al, and Si, and their ENMAX values are 250.00, 400.00, 400.00, 240.30, and 245.35 eV, respectively. The kinetic energy cut-off for the plane wave basis is set to 570.00 eV, which is about 42.5% higher than the maximum of ENMAX (400.00 eV) of all atoms involved. The K-mesh of 1 × 1 × 1 is used to describe the Brillouin zone sampling. A quasi-Newton algorithm is used to relax the ions. The self-consistency loop will break, and the structural energy minimization will stop when both the total energy change and the band structure energy change between two iteration steps are smaller than 1.0 × 10^−7^ eV per atom; the ionic relaxation will not be updated if all forces on all atoms are less than 1.0 × 10^−2^ eV/Å. These calculation parameters have been tested. The total energy of species is almost unchangeable with increasing cutoff energy (Figure S2 in Supplementary Materials). If the K-mesh of H-FER increases to 2 × 2 × 2, the calculated activated internal energy changes slightly within ±1.00 kcal/mol, and the reaction heat changes slightly within ±2.00 kcal/mol (Table S1 in Supplementary Materials); however, the calculation is not easy to converge and the calculation time grows rapidly, and then the calculation becomes somewhat exhausting, and the expensive computational resource is a bit unbearable. Taking the frequency analysis of TS6 computed in a node of Dell-R630 for an example, the calculation time is about 434,713.13 s when the K-mesh is 1 × 1 × 1, but the calculation time is about 3.80 times as long as the K-mesh of 1 × 1 × 1 when the K-mesh becomes 2 × 2 × 2. Herein, the computational node of Dell-R630 is from Shandong University (Qingdao Campus), each node has two CPUs of Intel^®^ Xeon^®^ E5-2670 v3 and 64 GB memory capacity and each CPU has 2 sockets and 12 cores/package. Therefore, the selection of calculation parameters in this study could be ascribed to the tradeoff between calculation precision and computation efficiency.

**Scheme 2:**
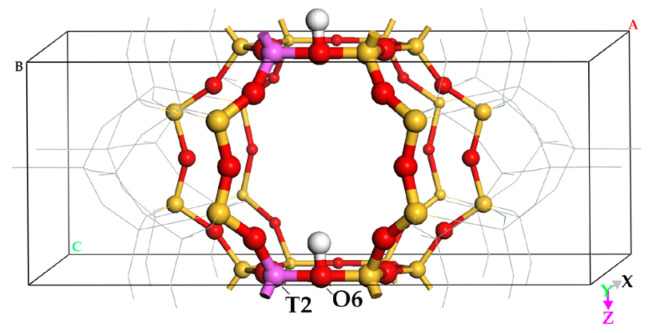
A fer cage (highlights in ball and stick) within the T2O6 configuration of the acidic zeolite ferrierite (H-FER). Note that grey lines stand for interconnecting atoms and bonds away from the fer cage.

A structure model with a Si/Al ratio of 35:1 is selected to represent the topology of the acidic zeolite ferrierite, denoted as H-FER. In this model, one unit cell of H-FER contains 1 Al atom, 36 Si atoms, 72 O atoms, 1 hydrogen atom, and 1 fer cage (Scheme 2). The unit cell of H-FER is a variant of a standard unit cell of the pure silica FER framework. The H-FER unit cell is derived from the pure silicon FER framework by introducing an isomorphous Si → Al substitution at the T2 site. To maintain charge neutrality, a hydrogen atom is introduced to the framework O6 position. In fact, this specific configuration is one of the Brönsted acid sites of the acidic zeolite ferrierite called T2O6. The pure silicon FER framework with the space group of Immm is initially obtained from the Database of Zeolite Structures in the internal zeolite associates (IZA) [65]. 

Through the standard prediction procedure [66,67,68], the unit cell volume of H-FER at the T2O6 site could be obtained through the E(V) curve (Figure S3 in Supplementary Materials) fitting with the Birch-Murnaghan equation and the following calculation of the full geometry optimization. Herein, the E(V) curve is obtained through a series of single-point energy calculations at some given volumes (ranging from 1900–2360 Å^3^) with an interval of 20 Å^3^; the full optimization of H-FER at the T2O6 site is carried out by relaxing the cell shape and the fractional coordinates. The calculated lattice parameters are *a* = 18.982 Å, *b* = 14.324 Å, *c* = 7.541 Å, and *V* = 2050.526 Å^3^. The calculated unit cell volume of H-FER at the T2O6 site slightly (i.e., 30.657 Å^3^) deviates from the experimental equilibrium volume of zeolite ferrierite [27,28]. When small attacking molecules (i.e., DME, CH_3_OH, H_2_O, and CO) are involved, the geometry optimization is performed by fixing the cell shape of H-FER at T2O6 and relaxing the fractional coordinates. 

The second derivatives (Hessian matrix and phonon frequencies) are calculated using the density functional perturbation theory (DPFT). Besides the 3N-3 positive frequencies, each stable species (i.e., reactant, intermediate, or produce) still has three small imaginary frequencies (<6.49 *i* cm^−1^), although many efforts have been made to remove them. Each transition state has 3N-4 positive vibrations, one big main imaginary frequency, and three small imaginary frequencies (<5.80 *i* cm^−1^). Herein, N represents the total atom number of species in the computation model. Each of the small imaginary frequencies shows that the associated atoms oscillate nearly along one direction. It is reasonable to regard the associated nuclear motions as the translation/rotations of the species. The entropy contribution from those pseudo-translation/rotation degrees of freedom is almost equal to that from a harmonic vibration of 12 cm^−1^ [67,69,70,71]. To reduce the computation error as much as possible, three small imaginary frequencies would be replaced by a harmonic vibration of 12 cm^−1^ during the entropy contribution calculation. The transition state is further verified as the desirable state to connect two stable species through vibrational analysis. The charges on atoms of each species are analyzed by the Bader charge method developed by the Henkelman group [72], on the basis of the fully optimized geometry. The optimized geometry of the transition state is verified as the minima along the reaction coordinate by vibrational analysis.

## 4. Conclusions

The acidic zeolite ferrierite is one of the most effective halide-free catalysts to facilitate the fast conversion of DME to methyl acetate with high catalytic performances. DME undergoes a reaction with Brönsted acid proton to yield a dimethyl oxonium ion DMO^+^ (or (CH_3_)_2_OH^+^), also known as the protonated DME [48]. Six DMO^+^-mediated reactions (Equations (1)–(6) are designed, which are classified into DMO^+^ methylation and DMO^+^ H-abstraction. The probability of those reactions has been further analyzed by using PDFT, statistical thermodynamics, the transition state theory, and the Bader decomposition of charge. Detailed information has been provided in this study, such as the adsorption complex, the reaction course, the structural parameter, the energy parameter, the product species, the Bader charge, and the rate constant.

In view of the [001] plane, the fer cage of zeolite ferrierite is a bit like the abdomen of a person (Figure 2 and Figure 5). Within a fer cage, the courses for the reactions between the hydrogen-bonded DME and the attacking molecules are hypothesized to unfold as follows: (i) Initially, a DME molecule within the fer cage is expected to preferentially adsorb at T2O6 site perpendicular to the [010] plane of zeolite ferrierite; (ii) The Brönsted acid proton exhibits a strong tendency to transfer to the O atom of DME; (iii) Subsequently, the breakage of O-C bond of DMO^+^ take place, affording CH_3_OH and a nearly planar intermediate species known as methyl cation (CH_3_^+^); (iv) This methyl cation tends to transfer to O or C atom of the attacking molecules or to abstract one of methyl H atoms from DME or CH_3_OH; (v) The reactions involve the S_N_2-like transition states in either DMO^+^ methylation or DMO^+^ H-abstraction; (vi) Various highly active intermediates are produced, such as (CH_3_)_3_O^+^, (CH_3_)_2_OH^+^, CH_3_CO^+^, CH_3_OH_2_^+^, and CH_2_=OH^+^, contributing to the complexity and the diversity of the reaction pathways within a fer cage.

According to our study, there are some convincing pieces of evidence to support the hypothesis that DMO^+^ is a highly active intermediate, and six DMO^+^-mediated reactions are likely to occur at the optimized temperature range for DME carbonylation, running parallel to SMS-mediated reactions. (i) DMO^+^ has been observed in the IR spectra experiment [1,2,46]. (ii) As far as the activation internal energy is concerted, either DMO^+^ methylation or DMO^+^ H-abstraction is less than the experimental synthesis of methyl acetate [1,2], the barrier difference is 9.81–21.40 or 3.24–5.87 kcal/mol. (iii) At 463.15 K, the rate constants for Equations (1)–(6) are not too low, which are estimated to be about 3.4 × 10^4^, 1.1 × 10^2^, 0.18, 8.2 × 10^−2^, 4.4 × 10^−5^, and 1.4 × 10^−4^ s^−1^, respectively. Whether the reaction orientation is suitable, DMO^+^ is likely to act like the surface methoxyl species and then trigger and further propagate the sequent chemical reaction [1,2,6,7,12]. It is worth noting that the numerical values of energies, rate constants, charges, bond lengths, and bond angles are more or less influenced by the selected calculation parameter. The calculation parameter used in this study is based on a tradeoff between calculation precision and computational efficiency.

## Data Availability

Data are contained within the article and Supplementary Materials.

## Supplementary Materials

The following supporting information can be downloaded at https://www.mdpi.com/article/10.3390/molecules29092000/s1. Figure S1: The pore sizes of (a) 8- and (b) 10-member channels of zeolites ferrierite and (c) the molecule size of dimethyl ether (DME); Figure S2: Total energy of zeolite ferrierite dependent on the energy cutoff; Figure S3: Total energy of zeolite ferrierite dependent on the unit cell volume; Figure S4: Schematic structures of reactants (Rex), transition states (TSx), and products (Px) from Equation (x) (x = 1–6). For Equation (x), please refer to the Section 1; Table S1: The energy difference (in kcal/mol) between two types of K-meshes (i.e., 1 × 1 × 1 and 2 × 2 × 2) of H-FER; Table S2: The fractional coordinates for Rex, TSx, and Px from Equation (x) (x = 1–6).

## Abbreviations

BA	Brönsted acid;
MA	methyl acetate;
H-FER	the acidic zeolite ferrierite;
SMS or ZO-CH_3_	surface methoxy species on zeolites;
DME	dimethyl ether;
DMO^+^	dimethyl oxonium ion;
TMO^+^	trimethyl oxonium ion;
CH_3_OH_2_^+^	methoxonium ion;
∆E^≠^	the activation of internal energy.
