# Supplementary material for: High Reactivity of Dimethyl Ether Activated by Zeolite Ferrierite within a Fer Cage: A Prediction Study
Source: Molecules. 2024 Apr 26;29(9):2000. doi: 10.3390/molecules29092000 (PMC11085771; doi:10.3390/molecules29092000)
Supplement: Supplementary file 1 [file molecules-29-02000-s001.zip › molecules-2923590-supplementary.pdf]

Supplementary Materials for Article

High Reactivity of Dimethyl Ether Activated by Zeolite Ferrierite within a Fer Cage: A Prediction Study

Xiaofang Chen [1,2,*], Pei Feng [2] and Xiujie Li [3,*]

[1] Institute of Frontier Chemistry, School of Chemistry and Chemical Engineering, Shandong University, Qingdao 266237, China
2 State Key Laboratory of Molecular Reaction Dynamics, Dalian Institute of Chemical Physics, Chinese Academy of Sciences, Dalian 116023, China; fengpei@stdu.edu.cn
3 State Key Laboratory of Catalysis, Dalian Institute of Chemical Physics, Chinese Academy of Sciences, Dalian 116023, China
* Correspondence: xf.chen@sdu.edu.cn or chen_smiling@163.com (X.C.); xiujieli@dicp.ac.cn (X.L.)

Content

Figure.S1 The pore sizes of (a) 8- and (b) 10-member channels of zeolite ferrierite, and (c) the molecule size of dimethyl ether (DME)........ 2

Figure.S2 Total energy of zeolite ferrierite dependant on the energy cutoff........ 3

Figure.S3 Total energy of zeolite ferrierite dependant on the unit cell volume........ 4

Figure.S4 Schematic structures of reactants (Rex), transition states (TSx), and products (Px) from Eq.x (x = 1-6). For Eq.x, please refer to the section 1. 5

Table S1 The energy difference (in kcal/mol) between two types of K-meshes (i.e., 1×1×1 and 2×2×2) of H-FER. 7

Table S2. The fractional coordinates for Rex, TSx, and Px from Eq.x (x = 1-6). 8

Figure S1. The pore sizes of (a) 8- and (b) 10-member channels of zeolite ferrierite, and (c) the molecule size of dimethyl ether (DME).

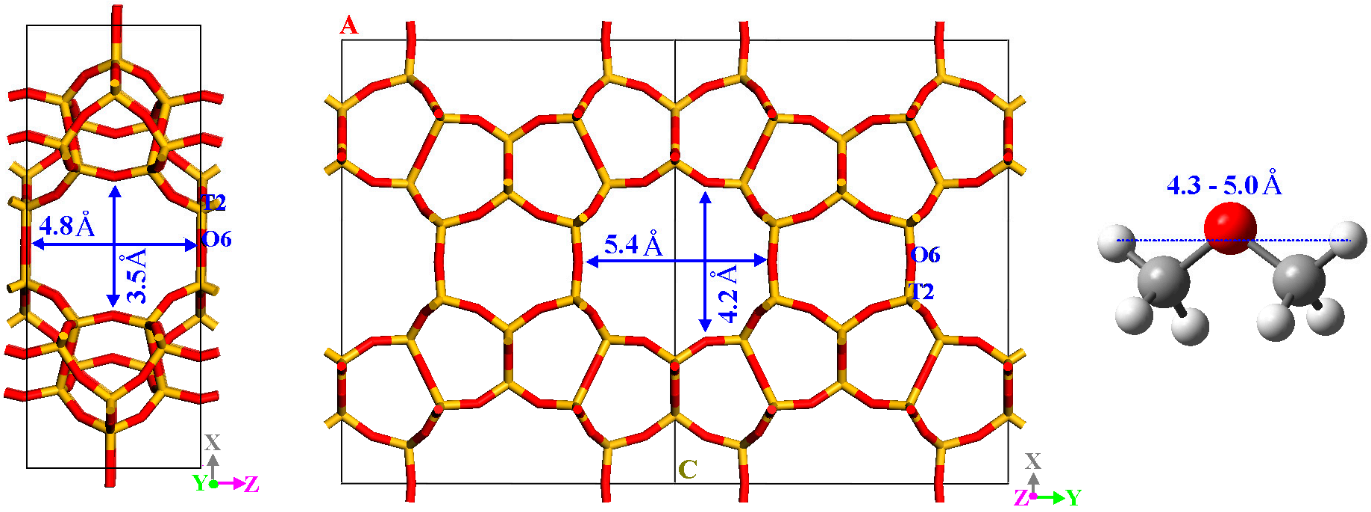

(a) (b) (c)

Figure S2. Total energy of zeolite ferrierite dependant on the energy cutoff

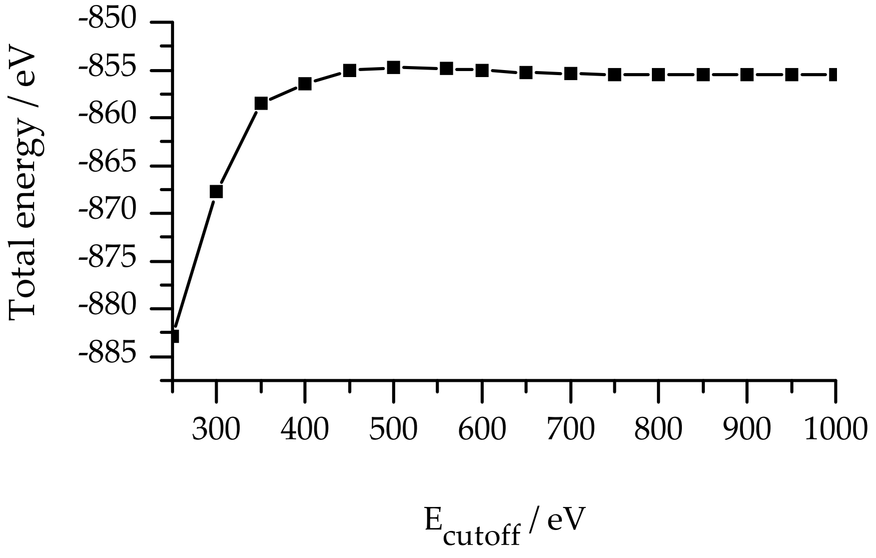

Figure S3. Total energy of zeolite ferrierite dependant on the unit cell volume.

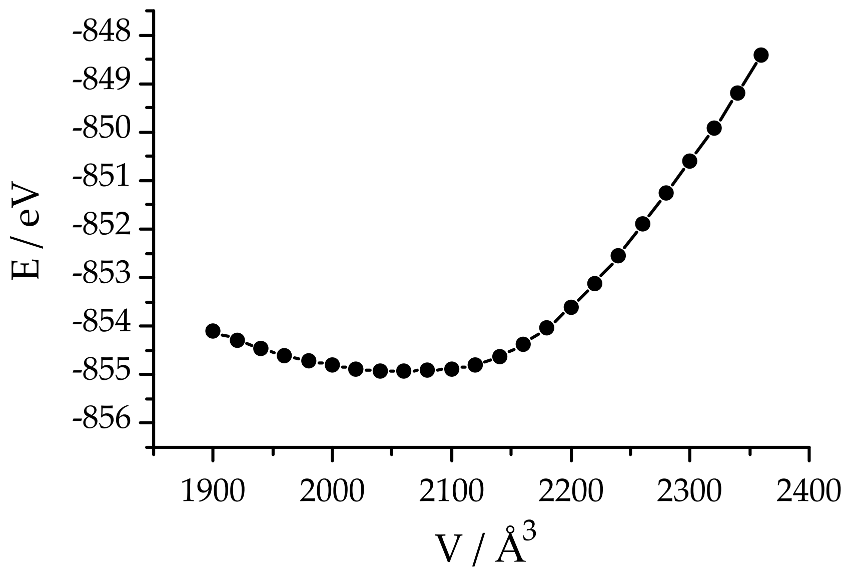

Figure S4. Schematic structures of reactants (Rex), transition states (TSx), and products (Px) from Eq.x (x = 1-6). For Eq.x, please refer to the section 1.

Noting that (1) O in red, Si in yellow, Al in pink, C in grey, and H in write; (2) the detailed coordinates are provided in following S3 in this Supplementary Materials.

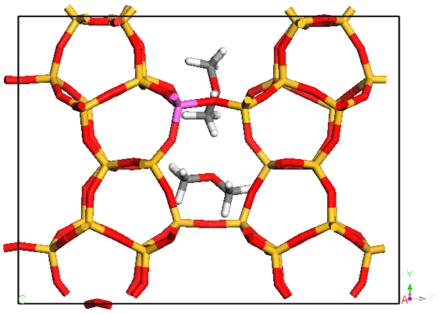

RE1

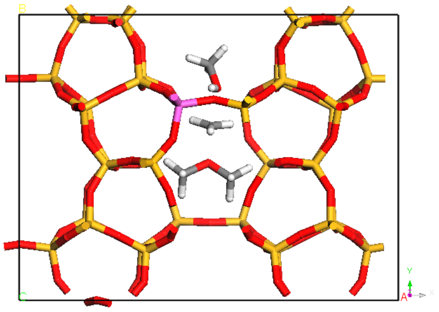

TS1

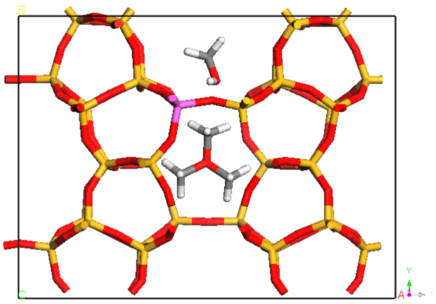

P1

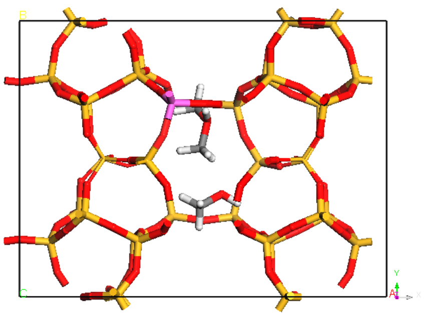

RE2

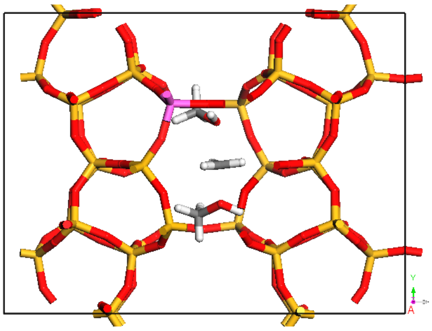

TS2

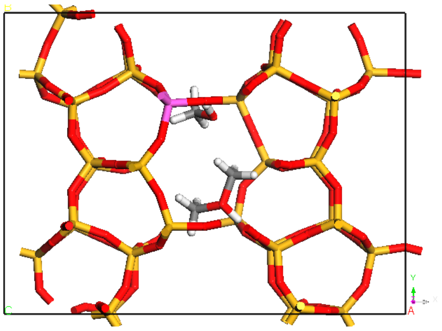

P2

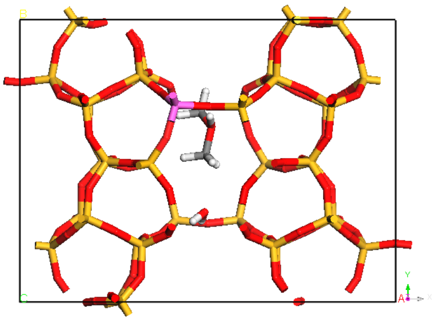

RE3

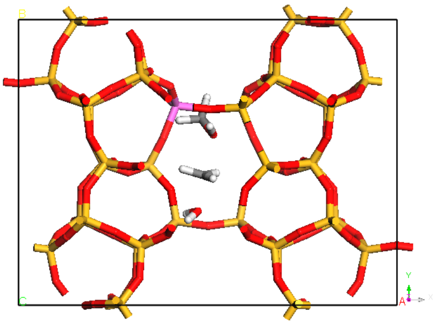

TS3

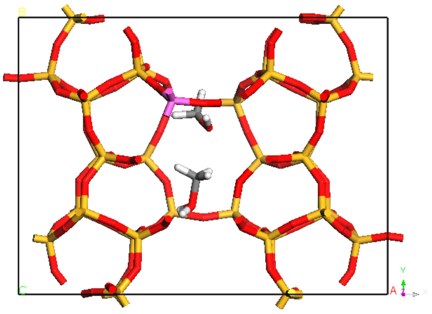

P3

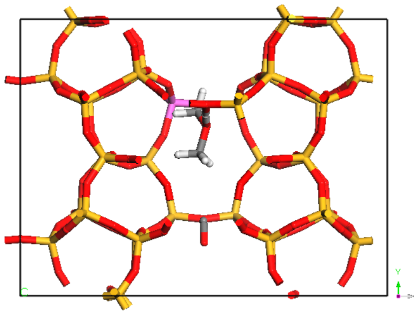

RE4

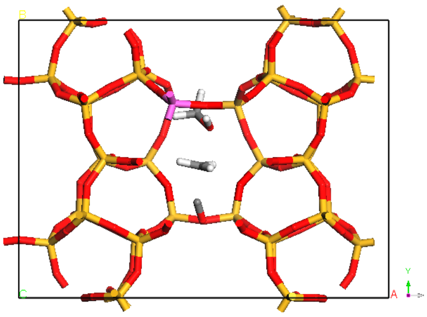

TS4

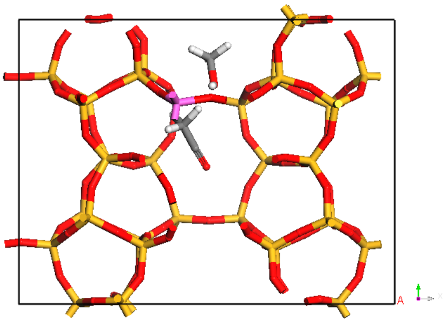

P4

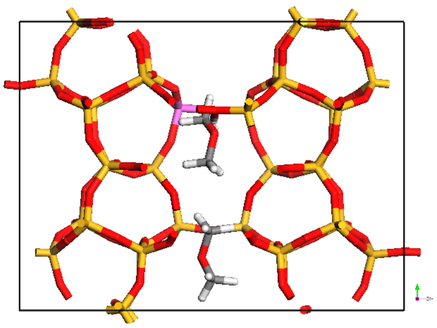

RE5

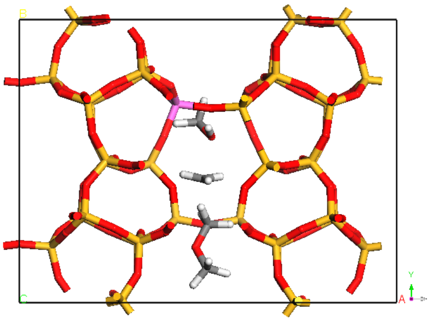

TS5

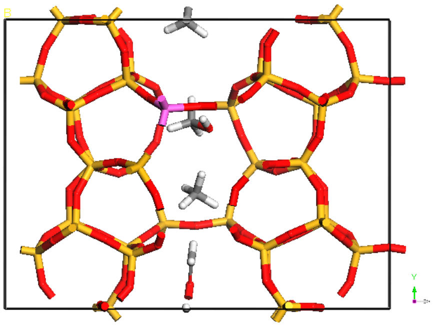

P5

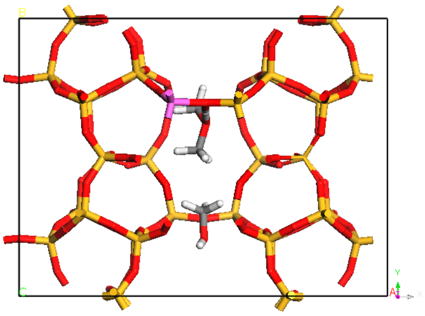

RE6

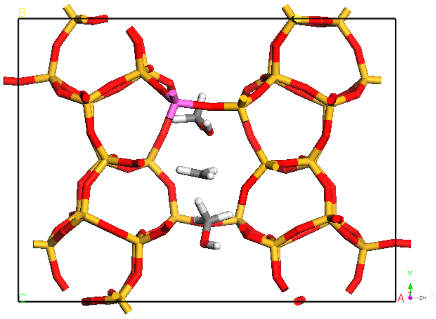

TS6

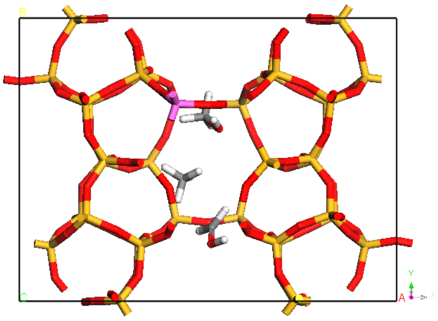

P6

Table S1. The energy difference (in kcal/mol) between two types of K-meshes (i.e., 1×1×1 and 2×2×2) of H-FER.

item	in kcal/mol					
	$\Delta E^{\neq}$(1*1*1)	$\Delta E^{\neq}$ (2*2*2)	$\Delta E^{\neq}$(2*2*2)- $\Delta E^{\neq}$(1*1*1)	E_r(1*1*1)	E_r(2*2*2)	E_r(2*2*2)-E_r(1*1*1)
Eq.1	18.47	17.70	-0.77	9.39	7.61	-1.78
Eq.2	22.13	22.97	0.84	14.78	15.79	1.01
Eq.3	23.49	22.97	-0.52	17.67	16.75	-0.92
Eq.4	30.06	29.47	-0.59	-8.37	-7.85	0.52
Eq.5	34.00	34.21	0.21	26.41	26.27	-0.14
Eq.6	36.63	36.65	0.02	18.43	16.75	-1.68

*Noting that 1) $\Delta E^{\neq}$ (1*1*1) or $\Delta E^{\neq}$ (2*2*2) represents the activation internal barrier when K-mesh is 1*1*1 or 2*2*2; 2) E_r(1*1*1) or E_r(2*2*2) represents the reaction heat when K-mesh is 1*1*1 or 2*2*2.*

Table S2. The fractional coordinates for Rex, TSx, and Px from Eq.x (x = 1-6).

Re1(H13C4O74Al1Si35):

0.5138429324990170	0.7288688809139430	0.7558005565670385
0.5093438279171778	0.8321353742116884	0.3968493583751567
0.5361824539989186	0.8822565488952208	0.6060155747878468
0.4457364644550381	0.8482580277826841	0.5709184239321203
0.5177085226575642	0.6647771467005725	0.3731287739090305
0.4371397386944409	0.6599489872256967	0.4997984813923182
0.5148787259904992	0.5984665060213246	0.5742139802721340
0.5734255452100996	0.3417491334143037	0.5140207032454072
0.5673661835847383	0.4452507383875570	0.6460685600310825
0.6152851208281973	0.4473675685340766	0.4411676811772125
0.4008906723518137	0.4376908110712776	0.4164635869848325
0.4454032934098251	0.3362893910553524	0.5010115932988342
0.4416732452327494	0.4408302767629237	0.6315530748167149
0.5008108431826059	0.8324805616095645	0.5403797784315856
0.4947205665952339	0.6599927062217361	0.5054141462851973
0.5690518863600218	0.4185756335664621	0.5083034235325457
0.4458908050678545	0.4133023572402834	0.4949809991749134
0.3307456882333426	0.0648518279204922	0.7683680602016452
0.4037436349709722	0.2185575452770649	0.8195945858041398
0.8295195595104872	0.5724492240946546	0.2509557086213832
0.8790652600725863	0.7404948646701612	0.3292598594202119
0.6953601188261160	0.8879667897153496	0.7156106849075314
0.6452219736634461	0.7261922205622611	0.8284286991951006
0.1851938302203635	0.3943075509853671	0.2278634708495346
0.1185230823992427	0.2397512736873253	0.3241106994712126
0.6905219499373203	0.0692811771367801	0.2296633101231791
0.6252663332435517	0.2302813279829081	0.1704272303372321
0.1737437196824345	0.5647935328956564	0.7287025188174425
0.1042350172331368	0.7216307741021240	0.6650238371197474
0.3239158749105613	0.8915417649700359	0.2549154505735771
0.4016045920326405	0.7424114592152975	0.1781093084127079
0.8269834742426099	0.3870399185307605	0.7639544441446873
0.8847472518149715	0.2252334487884085	0.6740015588578174
0.6871223076416797	0.8863291872809285	0.2542564805866405
0.6131619316337691	0.7335880790393006	0.1743415492369706
0.1871557701288040	0.3833403251437346	0.7592770025678703
0.1293591548307162	0.2205158542662460	0.6778903605389104
0.3173673595460968	0.0735552046837142	0.2243540671075337
0.3907542216104929	0.2274856150297424	0.1743882370016152
0.8385473705655500	0.5695527706429502	0.7361490409898721
0.9136925468081714	0.7229187946983373	0.6723550945430574
0.3124696484383236	0.8861250379680357	0.7078898524231079
0.3805117825797737	0.7317989676642753	0.7978163563675764
0.8261682489634268	0.3897253606976179	0.2318428387145985
0.8964864741899774	0.2369049935379692	0.3195092817096992
0.6843226018315249	0.0689015271476094	0.7701818572947587
0.6115379340553773	0.2242431344620286	0.8176071376290395
0.1891618494760792	0.5768596093314855	0.2587612644848321
0.1356246197643713	0.7438000710977377	0.3198892205396078
0.3577495980727861	0.1774548549610699	0.4986632504126973
0.1013402235554111	0.0728794764487191	0.4741399371764459
0.8632397240373422	0.6909858803372089	0.9938839021659334
0.6080812398952560	0.5713598650638332	0.9972430679483750
0.6762482347057528	0.7491455027237492	0.4876714265062532
0.9037077170045578	0.8906399670292018	0.5216841090307014
0.1734668579648826	0.2514834984772207	0.0038017036951530
0.3900083408085493	0.3816016705710297	0.9790378166296492
0.6549100459699702	0.1787106405203289	0.4972506041756048
0.9089749259061009	0.0732066839889853	0.4812759690757105
0.1470235357985672	0.6832123077936672	0.9905267051228392
0.4097508205082576	0.5645312724276081	0.9953525362303282
0.3276904824262843	0.7489081435901994	0.4768655336201988
0.1106104716567771	0.8910590885800076	0.5216898897269644
0.8395257245842487	0.2511473570629192	0.0006647507312252
0.6204639716288227	0.3871135792964751	0.9824217869254710
0.2668013611817628	0.2311214418006458	0.7466366370991651
0.7778203434406947	0.7378106967042797	0.7269316140707573
0.7580781952270286	0.2299517693962372	0.2844921670455420
0.2614679312011603	0.7302758479111020	0.1689245438397080
0.7514526912126129	0.7234546824360706	0.1910009231855980
0.2574675153787922	0.2387263099342505	0.2864315303735054
0.2402937437497101	0.7290372463967003	0.7437785943376412
0.7474532159411069	0.2353724148949681	0.7387639640455816
0.0069315004929180	0.2047918814216771	0.5192954602457505
0.5142707163733746	0.7056259464226002	0.9233250332107374
0.0078043157777614	0.7756944666314070	0.4352898737776059
0.5067881570172617	0.2821475689331550	0.0186996833001061
0.2051280303230030	0.0044582668107580	0.6655127094733473
0.7073991183039468	0.4865202208791288	0.1825595160941162
0.8080109793280812	0.9983542724494896	0.6737658918027662
0.3078148419366116	0.4837236094358559	0.1827942018614550
0.8078188068234695	0.9760768013028169	0.3213503199893424
0.2975224976742865	0.4940264768964013	0.8298053753699151
0.2014784563929766	0.9782951963332849	0.3118120481854589
0.7151206186728842	0.4955218086277995	0.8271778115971458
0.2510978796036341	0.9666160050414092	0.9882319636684755
0.7620197055951223	0.4759503441954251	0.5005421491584769
0.7593031050422141	0.9655576057536521	0.9944551251563425
0.2536246164650464	0.4716251041172939	0.5002176797625779
0.5198861952195273	0.7405407497975247	0.6069443970116168
0.5083051291706724	0.4465628675052145	0.4114772801707289
0.4218519960806049	0.6840806671321786	0.9821131882839325
0.3387802401374245	0.1732296216580878	0.7082072181798083
0.8314490266373795	0.6816498809612739	0.1922374077520672
0.6987190855943624	0.7749992220734825	0.6907277622893560
0.1837755092266420	0.2811532083448043	0.2100698359087687
0.6823328850318404	0.1773282940189560	0.2937765306269142
0.1672146963687595	0.6746644865174645	0.7818630742406043
0.3298668159294493	0.7776115760307292	0.2681074191810850
0.8249278192400666	0.2744909530111315	0.7932658275756310
0.6829157693255326	0.7735492628839822	0.2778837772640657
0.1894068916579599	0.2717840281421289	0.7959636657780180
0.3303696676773527	0.1792708229265969	0.2945207903932356
0.8482506547273871	0.6797575953122674	0.7830424742845992
0.3158671670214659	0.7724011086292748	0.6863085607297208
0.8299843035444141	0.2768839861629431	0.2086271307839098
0.6751714767627917	0.1769347740136098	0.7064612927788474
0.1837966458425200	0.6833824506871480	0.1855030575354277
0.0903528677714860	0.1846766469917753	0.4985126659624655
0.5957399603924571	0.6833925685933337	0.9836079962808810
0.9262321125850335	0.7824061031351235	0.4905602283070465
0.4219610457533705	0.2780922523584195	0.9985603915136352
0.9239631415959551	0.1847688126958360	0.4986936730928377
0.0907425838936717	0.7823989454056672	0.4861636944723600
0.5907874922433720	0.2810061665705987	0.9978573339006616
0.2746580514818078	0.9792351655023452	0.7829872777419595
0.7815209392756586	0.4810526025340920	0.2913691496973030
0.7371625193023377	0.9801292766183778	0.7881993439464310
0.2342258469894531	0.4819937801385237	0.2912283448115858
0.7366505786485646	0.9750147224499344	0.2011996576382060
0.2287032125226034	0.4785212805186561	0.7057525024143203
0.2734448459355860	0.9772865507041573	0.1955400568238161
0.7855908421954325	0.4816668845304193	0.7076722412174874
0.1555125237495574	0.9858072308738315	0.4926373528969705
0.6630706195653104	0.4842745780611324	0.9972247478812619
0.8570083652668217	0.9841945801134457	0.4986862992345209
0.3523492279807456	0.4832000495197377	0.9968658845677396

Re2 (H11C3O74Al1Si35)

0.5053514223413984	0.6683385203704901	0.7757797671973847
0.5065697723019653	0.6429520320117916	0.3687736370235086
0.5032944838356741	0.7503909605375370	0.4922974829453395
0.4268673618820316	0.6764087781447330	0.4827661262672223
0.4291324918598463	0.5311936640027426	0.6333696636755946
0.5000014624374316	0.5096482054869469	0.7868294301851151

0.5147755390817821	0.4933621713979477	0.5527066336545019
0.5949039874511755	0.3425330674378202	0.4558263060203984
0.4462160237349337	0.3591495522038741	0.4264610959336252
0.4912863807068462	0.3211796530773512	0.6217594191813021
0.4933784367945080	0.2518818735786681	0.4246652194235878
0.4844167930328567	0.6784939389077707	0.4845928783972653
0.4864576660055135	0.5336993596508890	0.6533665223335703
0.4934450449818044	0.3237672482383118	0.4762375526408889
0.3173495064487355	0.0922970844760371	0.7311284079686189
0.3947049146919852	0.2379467826834940	0.8284522093380247
0.8216695078431542	0.5854497263260967	0.2327934310381892
0.8727930054374724	0.7498931027449629	0.3340684828665701
0.6841798280946492	0.9060746266519288	0.7517420509250670
0.6357532642023642	0.7383504135776775	0.8352559096371124
0.1666592204238313	0.4176339351265739	0.2675836915511667
0.1018156347665169	0.2568947114535192	0.3251326128335634
0.6864626309156705	0.0855781167674934	0.2752933247531431
0.6273821793986585	0.2427128979502555	0.1633498524969568
0.1789494951312065	0.5909057877456121	0.7596909662386082
0.1037825535590002	0.7405866855895553	0.6715243870774827
0.3228745158968849	0.9210532887499312	0.2153104840726314
0.4017135867628809	0.7686962783722677	0.1885050634749774
0.8209829827810182	0.4050379324718776	0.7513070587124133
0.8729529960493565	0.2388475783983424	0.6679761761428580
0.6813459105559971	0.9039178946892861	0.2404408696181122
0.6025895283501512	0.7539693203150222	0.1795474286749226
0.1804795017929735	0.4082530726789102	0.7370267516171367
0.1282440388958861	0.2397636707176005	0.6734378850836364
0.3083817222938094	0.0989809771554206	0.2838130513979849
0.3764097962590682	0.2504917670118729	0.1808849623358313
0.8229222379070080	0.5878271436452636	0.7582947039248182
0.9017762725043923	0.7355936142537516	0.6812373486355341
0.3104447785945226	0.9106421699337020	0.7805118164782741
0.3759042879074102	0.7468072647930057	0.8086899255488049
0.8312148645867055	0.4034745348177182	0.2659663797477805
0.8993171777968740	0.2448141964019968	0.3195745603728781
0.6779620709224332	0.0889977182513348	0.7434936683636337
0.6034635894983893	0.2423005561316600	0.8166141346008828
0.1874723365722062	0.5973751156488945	0.2272112337109320
0.1271585522591963	0.7563008089040792	0.3212720744535176
0.3339911497108972	0.2351938126133390	0.5121181312142582
0.1008975985835150	0.0899014999677732	0.4768455535135203
0.8419628929614476	0.7206586817116474	0.9976301200966589
0.6115938062250805	0.5865645295395367	0.0267454860435095
0.6548526620096027	0.7832518710149472	0.4991540961628758
0.8990017947561029	0.9014527903686229	0.5243640821740527
0.1466259496043563	0.2963682641902281	0.0037458170182489
0.3981696850978977	0.4039284537104777	0.9893770967464235
0.6596913903751158	0.2227650844527673	0.5015332126734009
0.9019647263450423	0.0837550086170964	0.4918125593671689
0.1622603089976025	0.7290559865133019	0.9890730177634737
0.3922483586259631	0.5862041446269757	0.0306945678288528
0.3433385120065324	0.8139005390240470	0.4942971438914157
0.0973538806573160	0.9073268057620041	0.5121569486800226
0.8485983845467899	0.2815066091447562	0.0025252510466913
0.6036070464483601	0.4034201459237252	0.9892042932385507
0.2571397747836102	0.2566395747608041	0.8059534146683092
0.7649161741228809	0.7556987286386487	0.7081486612459429
0.7613419628809481	0.2423799384789049	0.2672235361370880
0.2627381043658374	0.7533854340052883	0.2286253836467722
0.7393266286398656	0.7341502704196756	0.2317078019226031
0.2395116789160383	0.2593932846038456	0.2589023260152885
0.2414636225715583	0.7547372143990785	0.6996857980927729
0.7417423108477692	0.2534503254607543	0.7843632012499029
0.0009577373441232	0.2114330168832552	0.5498957007568777
0.5071903836322065	0.6993018255159171	0.9351457530348313
0.0006378372705385	0.7798745388258439	0.4504611643167635
0.5003059810787889	0.2808436105385255	0.0374776928278280
0.1954059143149038	0.0050846493846421	0.6783172947960949
0.7085849629463894	0.4775425007753142	0.1724125386567721
0.8000197597573973	0.0046301096281240	0.6759296022556072
0.2935087336356688	0.4765259043071310	0.1727280409030243
0.8037864860085477	0.9851712928143499	0.3222377252398303
0.2997867744887870	0.4987158940056489	0.8202394055762170
0.1982932244572524	0.9852625567329483	0.3232067694248073
0.7018430284813277	0.4986624297028044	0.8189464231582235
0.2441485995457526	0.0257098617904177	0.0021530542297938
0.7547967754588853	0.5004745403822994	0.4978911737461118
0.7502523695323760	0.0048366110575060	0.0012025792566277
0.2489762880261992	0.5090098281616235	0.4970336372475614
0.5105520161651995	0.6302517695799779	0.6413294812557311
0.5531143858730587	0.3757571813813243	0.4148648248156661
0.4143299767787738	0.7023659177125552	0.9991796641013480
0.3250971887516414	0.2049095321700278	0.7184583123286714
0.8195780546167981	0.6976885965118527	0.2003578701043196
0.6850773091914846	0.7958814259012712	0.6999760044346459
0.1640795817073268	0.3075743777423199	0.2136349086144378
0.6839413542353441	0.1982165675580916	0.2989263809403298
0.1725419392049474	0.7038026803945456	0.7810384515447666
0.3334123900837724	0.8133040133913241	0.2805709950629804
0.8214900597519232	0.2945428563839059	0.8002109050453114
0.6705971249678981	0.7944522120480784	0.2886670602688710
0.1780213566416293	0.3006120923547826	0.8041475835165173
0.3142713652309297	0.2110985710005906	0.3067914166763188
0.8328758190619965	0.6994670132552656	0.7875031929710872
0.3188494160413313	0.8052332783260923	0.6995205284219210
0.8348589210099888	0.2933238912727987	0.2131030735601485
0.6712958374180573	0.2009568829311661	0.7123366700490976
0.1855397073685623	0.7094653522607643	0.1931848976204975
0.0839558588754201	0.1997549306553381	0.5057651966628640
0.5899478556353799	0.6952612865222889	0.9969970499750076
0.9186729061379850	0.7918931076960547	0.4978827561632286
0.4160002742785807	0.2939933891576700	0.0095108821906820
0.9187884070540946	0.1946768114087831	0.5072614179603576
0.0838573128890090	0.7958753157499103	0.4895694267674955
0.5834743875971000	0.2932345174635955	0.0048683644993659
0.2669463538159818	0.0071017837181273	0.7973571133430895
0.7790934228750857	0.4923116591183074	0.2922061354631253
0.7285345022990342	0.0009049332305793	0.7927451931816307
0.2245511456801523	0.5011039793487626	0.2906659988739264
0.7309755579206580	0.9948729919323753	0.2097860332034429
0.2273836225862951	0.5019652941402697	0.7050091794395783
0.2682070712970201	0.0072716027758872	0.2062666992175295
0.7750516751043435	0.4978471746064628	0.7072334697306104
0.1490003155153801	0.9969798986761091	0.4976731583120753
0.6569383331980347	0.4913940635685563	0.0022801064836884
0.8508259561965517	0.9937180695871746	0.5034030099669735
0.3459560793359273	0.4944840145321336	0.0045429417967995

Re3(H9C2O74Al1Si35):

0.5045302250429984	0.6568567713162281	0.7755464768182776
0.5053324708389226	0.6388679648777469	0.3680283042336541
0.4982924699134941	0.7427816389144937	0.5010544145605635
0.4244945337815622	0.6646007950982380	0.4839466513141062
0.4309688814221815	0.5163066086668380	0.6343608777009280
0.5130233151857624	0.4857231053871800	0.5302588127700076
0.5079061656862152	0.4925681738086141	0.7677278840601858
0.5256972128448965	0.2846985428908368	0.4169438127451954
0.4516423183173757	0.2882471495167636	0.5008239085795410
0.4819523605783971	0.6700594246043323	0.4867747113767962
0.4886068777286496	0.5214368672366163	0.6425538468686085
0.4955339544073425	0.3231986540814376	0.4910622533919451
0.5096846879545893	0.6195390896432187	0.6392799102098436
0.3205471635053740	0.0882371367394654	0.7319889612367305
0.3959212994329988	0.2372424838093181	0.8230085475911437
0.8140873462779012	0.5881167220360908	0.2265399993066097
0.8722547396228322	0.7473526418680763	0.3297761745806156
0.6866288377264311	0.9006525036782733	0.7542004637422721
0.6341763862994227	0.7347000442201690	0.8334756494794036
0.1635153476671576	0.4135797902566978	0.2621976798680095
0.1021756145166890	0.2506431994776790	0.3205764564173776
0.6828130081977761	0.0912680283552092	0.2646033192326129
0.6199593666729939	0.2527441287992005	0.1916720565759036
0.1796188826988114	0.5870220645104780	0.7592123377622357

0.1032914287735736 0.7344879583558708 0.6667594482468289
0.3186789286891809 0.9160457963923818 0.2108539760090338
0.4000353826057221 0.7656325458901918 0.1834242020243693
0.8190577190762625 0.4011457093866397 0.7423974405562959
0.8752695381048383 0.2351578868513755 0.6819094695126182
0.6732515563776786 0.9085629880090735 0.2494760940171048
0.5999730234231819 0.7544502423071506 0.1759790174997420
0.1802495589469260 0.4044246627071431 0.7376990751640733
0.1272035453773555 0.2368621039530439 0.6702768584048542
0.3041149724894581 0.0942729508172704 0.2822256291211502
0.3758048965046115 0.2422617306732917 0.1737638291760462
0.8253638125408429 0.5839706569922356 0.7646955920134104
0.8996016932442927 0.7351098682626613 0.6784221862305699
0.3106567192930925 0.9066739382433866 0.7782518247977848
0.3752360599610967 0.7424231614365340 0.8037443446569057
0.8300532405186232 0.4072594445258417 0.2759755980388121
0.8946521173737239 0.2462620919596361 0.3300596709809724
0.6798982362108461 0.0831765036389882 0.7489514863611717
0.6068426134252931 0.2334857010418574 0.8375678851006754
0.1817705848030613 0.5943458500182430 0.2284248182929431
0.1252897621364539 0.7564037911306372 0.3160495018002365
0.3399638233607973 0.2282034968740092 0.5066613023830868
0.0986639051804445 0.0855372111098092 0.4783229544120928
0.8379737350909409 0.7230810465613970 0.9935105643694939
0.6117562044761513 0.5852448661321006 0.0323438700217977
0.6505287906493606 0.7819665012477941 0.4984890405011555
0.8978353140703632 0.9002173018746120 0.5159718820917831
0.1501345024231995 0.2885779264522554 0.0015177827723747
0.3952315799992405 0.4004030302117059 0.9938270409946099
0.6619914698435423 0.2186956966902542 0.5152150691291695
0.8995221620137102 0.0828690821179023 0.4926522333253303
0.1618572716219191 0.7259655213133840 0.9857514415933935
0.3904340037336667 0.5827899450309886 0.0279878587316773
0.3426964640261048 0.8109316245945521 0.4903765632779340
0.0973865754419947 0.9035026951432172 0.5183096925589012
0.8493833834422020 0.2882348230804084 0.0104949973711754
0.6019065350369743 0.4025654449744351 0.9864025841884327
0.2577639802213767 0.2521565531133447 0.7921582429607952
0.7620422443605435 0.7464193862995927 0.6971135212367301
0.7572731698332902 0.2482870547709339 0.2651958716238028
0.2614990769930827 0.7464574685614522 0.2293448646514094
0.7376516047864641 0.7431232605722897 0.2305460836314026
0.2405925620557881 0.2592191005030386 0.2668591648151661
0.2406407414035741 0.7518344500931136 0.6959421437338449
0.7437904235661534 0.2473777993589792 0.7979347802476298
0.0001439671829715 0.2094023482255096 0.5452536925867406
0.5060043874021929 0.6925895735739402 0.9315384414451344
0.9994806383888317 0.7778899909408281 0.4504213773673911
0.4991778855963460 0.2804892354694815 0.0392602507869810
0.1985620289262755 0.0041068361577317 0.6664372658982813
0.7049447655617627 0.4726274626504008 0.1805460040822311
0.8035713013252419 0.9997952961858942 0.6914811403537513
0.2884485248984348 0.4761669252863854 0.1632533933864551
0.7962106321318174 0.9866766066410264 0.3382511166461342
0.3007340456641501 0.4943550205543232 0.8111372957994121
0.1933365411901704 0.9814100233462426 0.3125020722414220
0.7031311179552020 0.4976139162751352 0.8267285894146497
0.2427636587465543 0.0225870576875096 0.9951741612793654
0.7525354351008815 0.5057948347388148 0.5025678370145314
0.7496775526976478 0.9984812160357706 0.0102758999695425
0.2462846627631805 0.5042797966642993 0.4915696623434584
0.4131192893910836 0.6984835911311933 0.9951786180066833
0.3273284896461348 0.2006456261001688 0.7138345477661829
0.8159463575987473 0.7005160461329965 0.1968151438463011
0.6835827630362488 0.7913745135159331 0.6971694394488992
0.1644188570359049 0.3030101650622186 0.2120392048515285
0.6811774635225376 0.2025523414166841 0.3077735855013586
0.1722653084164136 0.6998789084006347 0.7780230070275493
0.3315233410031979 0.8088055796373439 0.2770409064607051
0.8221667451006880 0.2929324029042917 0.8069249045692359
0.6662928105612522 0.7974313053539888 0.2888254669577250
0.1786729133784419 0.2959421504663950 0.7996167488698944
0.3143264251312488 0.2058654181537136 0.3040381613360736
0.8313845751939226 0.6968456682850857 0.7847540108772293
0.3183567894281580 0.8015207257846626 0.6955622657390848
0.8324512063192415 0.2973322297169645 0.2201227446979743
0.6733138339622187 0.1956432252415539 0.7240470054585657
0.1832609063263604 0.7062312369907886 0.1911988511559954
0.0830107616323374 0.1959395755853990 0.5031410687597386
0.5885318825557562 0.6928800736732796 0.9962170604048666
0.9172289354156291 0.7903504878798628 0.4942550345394849
0.4151937798545831 0.2912652272642973 0.0075043746074570
0.9172440718040065 0.1933886999694181 0.5123614472493969
0.0828492112031185 0.7927113152050751 0.4884985063399725
0.5825334398602635 0.2923746775976426 0.0116048512086024
0.2681046223708137 0.0038919562274131 0.7922238548993334
0.7756140513563921 0.4940799749640874 0.2966481795019362
0.7301745213824091 0.9954399119901112 0.8008093500788718
0.2205250350931180 0.4981511059879580 0.2859684367563844
0.7259037806562390 0.9965728410890335 0.2165140314109948
0.2270566815236563 0.4978039323681926 0.7009693904516539
0.2646420465119590 0.0028679184325853 0.2003739220459337
0.7750539153654969 0.4967178586197107 0.7099170367369751
0.1479540728487478 0.9935287585003110 0.4937974120967681
0.6561625231021679 0.4894634291171869 0.0059795043443032
0.8489781002555574 0.9924124343189078 0.5090882349921912
0.3437738506557722 0.4916217944274095 0.0005360746564946

Re4(H7C3O74Al1Si:

0.5043977903800752 0.6544902803295187 0.7971678829665763
0.5080602062028703 0.6289196835024455 0.3737668731567183
0.5000513891014506 0.7328863940801398 0.5059350828882145
0.4260185219573529 0.6559300027283470 0.4820817021508574
0.4295996901107912 0.5074908717880717 0.6293971738324871
0.5140671820597333 0.4767039358331999 0.5382573541921261
0.5028217902913283 0.4837832027835773 0.7745154504263283
0.4835344713028888 0.6601958979112155 0.4913481867335250
0.4869903976155499 0.5130498320523813 0.6465875302357489
0.4994375196854364 0.2630055679505432 0.4977816107814093
0.5004896914091717 0.1836196611177385 0.4829462574114487
0.5082125046213761 0.6102492560917980 0.6464911442756289
0.3137361317779792 0.0904691973948104 0.7327109842837771
0.3934018861486663 0.2330717695346394 0.8343338025681177
0.8160614939776707 0.5896282193551627 0.2233937282245435
0.8726239832667773 0.7498174701869047 0.3282762463225311
0.6854343466970434 0.9056879397871711 0.7530460831786954
0.6335370127364328 0.7389380746458940 0.8303011278738097
0.1675091146380225 0.4153338207667403 0.2726870101731009
0.1029724873104882 0.2544184896666906 0.3279311828359184
0.6859910198358605 0.0920628268395021 0.2695530835516493
0.6249171569775811 0.2520296021999329 0.1802630790709827
0.1778099454073327 0.5899375321812457 0.7617063438669689
0.1033581182717356 0.7403764239982351 0.6749164816163997
0.3253897910564092 0.9186519228812600 0.2194077086294541
0.4029902428334822 0.7648263088091838 0.1918028952164619
0.8217653407991037 0.4048864576266240 0.7391592170350165
0.8747060893665974 0.2372891086431395 0.6737482516549562
0.6746540188697310 0.9096868355496071 0.2402385053554994
0.6003114259336115 0.7557692687310791 0.1744241309646881
0.1789899357026243 0.4070452994485976 0.7370721631599721
0.1275351527129089 0.2377116964185433 0.6772228656420083
0.3106077085869430 0.0974507827333042 0.2848146854736413
0.3785408146005622 0.2503505843990581 0.1887284461179490
0.8229575586855660 0.5879050513708464 0.7625695881475920
0.8992932979428332 0.7371382831766269 0.6769518892669097
0.3104510566271301 0.9086258586381817 0.7819892328631681
0.3748382868864866 0.7444223728693018 0.8137826583994467
0.8324535740045675 0.4085116745086310 0.2724757712582928
0.8991065397957598 0.2487250830095249 0.3246208553047651
0.6821176119467367 0.0887933099494944 0.7438074889238635
0.6076597657465257 0.2397122530508753 0.8265757524963107
0.1891228192684480 0.5949846225007747 0.2255923502039465
0.1277514382643190 0.7521638581170862 0.3245395251040648
0.3324066928234544 0.2337651747007286 0.5164542646776198

0.1026502587985121 0.0876588678489512 0.4791017716897272
0.8375841317744204 0.7261292859767323 0.9921980987741179
0.6151833668592133 0.5875560910391968 0.0282272741112095
0.6505910660351120 0.7869663525005279 0.4962384011460372
0.9005657932516513 0.9021453180407164 0.5146296594832549
0.1458313915741556 0.2963949396757499 0.0064414664799344
0.3973009399210952 0.4007851066235091 0.9874579063915832
0.6679337871327391 0.2255402084363922 0.5098461405256032
0.8990405178527610 0.0847648663573537 0.4858976989477881
0.1636457686446207 0.7285144112439923 0.9913746180699334
0.3922802836885495 0.5824163075034150 0.0324529946033465
0.3451833928696004 0.8109032251689001 0.4978842383997275
0.0966304978770935 0.9051483850269122 0.5082486123031700
0.8542424827055726 0.2915075654535784 0.0046964307402817
0.6012921689498043 0.4051342743674695 0.9884310971934056
0.2564014191734714 0.2565189256932641 0.8100402806794662
0.7618712900751206 0.7521716392117881 0.6967663205459615
0.7618217866888912 0.2476364414662982 0.2557429693712407
0.2636833406371792 0.7518437468814838 0.2325592033069626
0.7376511953024334 0.7427291805500644 0.2315993289129779
0.2404252233258077 0.2567623391409484 0.2563628128238804
0.2412620289916703 0.7530451772087190 0.6988775579362851
0.7451869995502136 0.2528900252039250 0.8013543810950736
0.0012226523493766 0.2074650087475263 0.5487227173615992
0.5068111031358598 0.6909960257145400 0.9323903203435151
0.0004554648574810 0.7765481041847409 0.4501484820299808
0.5006070517038381 0.2801871231329969 0.0361839417266552
0.1927708370520946 0.9994071773347031 0.6882877869637269
0.7076547618539806 0.4731952421239285 0.1739353883863330
0.8038150052386399 0.0004053914651294 0.6852281866297716
0.2945474869074545 0.4729085474233372 0.1780742658713521
0.7983688098954360 0.9836987205796177 0.3318658155891185
0.2978514940398966 0.4968110755960637 0.8254089488593124
0.2017901118150647 0.9825618925320327 0.3333894037427072
0.7028217585056709 0.4955375227463463 0.8194436744843330
0.2445737112020296 0.0217194623886456 0.0086376796689223
0.7538369452291249 0.5067189952318358 0.4973107026832082
0.7506841541247979 0.0034388912083259 0.0057377776314951
0.2488683886386909 0.5087172627720733 0.5005660222571677
0.4131562729107117 0.6986153336875063 0.0018598514642036
0.3234215344851563 0.2028232333703031 0.7224344499753812
0.8165192992363401 0.7022507625386964 0.1956382278403055
0.6832121523184398 0.7964818809778933 0.6952183967295724
0.1647658006226962 0.3056515202673324 0.2158953311895146
0.6852121699721465 0.2043967609579838 0.3025723721280968
0.1724001871782335 0.7029776159621477 0.7828575460264844
0.3350635797150119 0.8106200036466191 0.2843720914372838
0.8243837047175049 0.2965062667649860 0.8034015989074419
0.6668677797715574 0.7994626347072611 0.2862564670758232
0.1770490468486159 0.2997580877172652 0.8066380744435264
0.3151417828298690 0.2096521870032362 0.3097258339336122
0.8306046393358670 0.7005109606908562 0.7832273535171410
0.3188869442963025 0.8030704999675393 0.7016715215251281
0.8364512854855519 0.2989041975346467 0.2143015213854014
0.6759643472471524 0.2013404947884609 0.7192646228299182
0.1867032971837048 0.7074167602185426 0.1952700665803064
0.0845179763256212 0.1971212340220916 0.5078200232305647
0.5905546089820888 0.6944504806706675 0.9944349519002387
0.9182875495306320 0.7916814372665826 0.4928462019351159
0.4159039920404197 0.2914199982642671 0.0118721431891302
0.9185501588290137 0.1945930499827924 0.5080532703848775
0.0836126347011259 0.7933780279800473 0.4901693271716780
0.5839913202119149 0.2937347764047900 0.0078857281054781
0.2655799026829726 0.0038359627122659 0.8024810074566631
0.7777814914444292 0.4950837581139140 0.2919516695421933
0.7307887860991471 0.9995877063823428 0.7964463309132697
0.2253184378427946 0.4989923245306827 0.2938943824606000
0.7277887461544026 0.9977931642084386 0.2124694865452526
0.2261721541917296 0.5008091875847001 0.7077015271565159
0.2704223150450460 0.0047112670269982 0.2119128565334734
0.7754771226212753 0.4984625288259181 0.7053198171136117
0.1495059926617586 0.9938391628777694 0.5022353850919998
0.6575801201987517 0.4900632906253932 0.0018487151468776
0.8501479555900104 0.9929074323527018 0.5041919913548725
0.3453658958170323 0.4911555555176861 0.0070059610873727

Re5(H13C4O74Al1Si35)

0.5049185670157428 0.6568559608507769 0.7992158654216368
0.5077655207830176 0.6283671931154231 0.3720365042858802
0.4969406320326328 0.7325400110571465 0.5022678897844557
0.4250121587332458 0.6521560210398079 0.4795347568791115
0.5043950460797006 0.4858228449479896 0.7773717184446909
0.5182820116033682 0.4773266616122314 0.5419074801183115
0.4321953153132299 0.5051683877092117 0.6272595537310437
0.4832811275933437 0.2741481259462617 0.6324186391106537
0.4635548209282518 0.3161970897962547 0.4147167297542538
0.5507972030219221 0.2776579678144060 0.4675626556856329
0.4750787381763431 0.0367794054122399 0.4874218442601617
0.5585442607117841 0.0932448988642989 0.5143714570833851
0.4905724029238243 0.1108085874114906 0.6758933515516503
0.4824073910694011 0.6590543972971830 0.4891286984250556
0.4892595856675968 0.5134197016641124 0.6475670069967450
0.4943778698816672 0.2652067838797549 0.4904943827724608
0.5011368933838938 0.1008600467543630 0.5332126599898004
0.3110765385378969 0.0943290671632937 0.7277349792291119
0.3902490709523292 0.2358437549865187 0.8340082832966473
0.8161165213126793 0.5892155997792869 0.2244483342913313
0.8742461838580766 0.7487435110429388 0.3277697979483705
0.6850800390606224 0.9049506727608829 0.7527627611617120
0.6356955175364476 0.7369188070618478 0.8314109466925359
0.1690187802751524 0.4173912584370356 0.2729172048770607
0.1001799181211211 0.2593580140060254 0.3281522122952083
0.6868049534809089 0.0913709664080713 0.2708698456617995
0.6236034199736480 0.2499445159381395 0.1825286626994540
0.1781941048570346 0.5935739553500312 0.7574132617140918
0.1051508030226884 0.7460232858436626 0.6758153692862692
0.3275676113257830 0.9220217023345754 0.2183637903844371
0.4046183782889727 0.7677533698912313 0.1922046751072983
0.8215754769828010 0.4041079116802209 0.7379045886071793
0.8740428929902819 0.2359555167029725 0.6738310206274107
0.6741546916275922 0.9091734626237411 0.2427944516061302
0.6015974271650890 0.7541449724401446 0.1751448860245475
0.1783172267990878 0.4106517677206085 0.7363496002697971
0.1258438549188909 0.2417724821929284 0.6766946483899616
0.3130805789726239 0.1009764997746387 0.2838781863951922
0.3738355355998593 0.2599218108808827 0.1860057618811837
0.8221273814523471 0.5871707399100927 0.7628152968931587
0.9007727946813233 0.7344594202957353 0.6757874888697017
0.3113705631766877 0.9126977920613868 0.7804838220423918
0.3769607254967582 0.7493515861456288 0.8137035389107794
0.8326194292516078 0.4080576042779498 0.2738043789114144
0.8982252098892403 0.2472660922957246 0.3250213682159711
0.6826539179988629 0.0881140245058845 0.7455168453923164
0.6082211839167400 0.2386001067376995 0.8282562018230024
0.1921584422258249 0.5970161095783695 0.2240355060777546
0.1294375048401406 0.7530524379113572 0.3252140058993973
0.3256631717159024 0.2410137673827961 0.5166179764888383
0.1050726835382108 0.0914167783709274 0.4763758597208323
0.8394461994114408 0.7246685890911451 0.9920502598814736
0.6145984003476244 0.5857847161665859 0.0279049520443024
0.6520054210015616 0.7847100346243536 0.4968444565386889
0.9006454885744688 0.9006220077852447 0.5169570119851343
0.1420766881581486 0.3016468333449822 0.0058329174563596
0.3996740415783222 0.4051043496075692 0.9811382677713993
0.6708741160803626 0.2251022423463240 0.5112584401460367
0.8986569589185720 0.0833916948599835 0.4862678980591113
0.1652822913681931 0.7306175765215173 0.9913978177866056
0.3920797976054473 0.5860171289828244 0.0313724792586640
0.3467298205631764 0.8145443830852557 0.4975754841269762
0.0968229568572525 0.9084519761304222 0.5005491053921602
0.8555977689252785 0.2923936635685322 0.0045908573294966
0.6010809023367045 0.4033799078036253 0.9911925443028267
0.2536671155611288 0.2590669253201980 0.8158745008066859
0.7637790244379161 0.7533688930428681 0.6976536520100822

0.7612042368429570	0.2481097186878714	0.2500985572712509
0.2652038000135875	0.7553671172594250	0.2321868546617694
0.7392650265918093	0.7437466627116223	0.2302078697379812
0.2367165198671515	0.2540049533209654	0.2509449754305351
0.2432707547612267	0.7558123234247702	0.6996165755391175
0.7455319572915187	0.2522987004651185	0.8061326802410278
0.0005534910953457	0.2060033114328021	0.5490837370736372
0.5078729535276025	0.6924106207205938	0.9320544461499054
0.0018386254737379	0.7773871792341609	0.4500381376924025
0.4991813948762527	0.2794813510772869	0.0320589927196053
0.1914604887965012	0.9994648586548607	0.6911455126772097
0.7079241075918574	0.4725751227210253	0.1736410529093249
0.8039128990804087	0.9989885205848665	0.6865627505976661
0.2966943388849899	0.4734907629168603	0.1785878529096365
0.7984263314560707	0.9815533383206088	0.3331307465458480
0.2974056875065472	0.4996223195387230	0.8265340307159477
0.2047575883322992	0.9854398122452537	0.3363121595556464
0.7022588959283738	0.4936517907502136	0.8191844683609943
0.2456894528353857	0.0245706686136913	0.0093810466828970
0.7532720334415757	0.5061813769605550	0.4972619996532615
0.7506121912748753	0.0014822151022571	0.0069903630747348
0.2503695478560033	0.5103947470118939	0.5004073754968132
0.5081514276752728	0.6111721205732437	0.6453072221099561
0.4729401660510533	0.1752745224676886	0.4304061468888776
0.4139317927360295	0.7018360533612267	0.0017128095807166
0.3199792158197994	0.2069748748089140	0.7214613583493161
0.8178038767125955	0.7017220215047928	0.1954412144942594
0.6844744034020849	0.7955164698876374	0.6957628883167928
0.1629405487560760	0.3080262873410575	0.2147527250628229
0.6855444039494714	0.2038416166915127	0.3022914278091449
0.1738612973968898	0.7064748705157058	0.7822490221288305
0.3366360690422496	0.8140381124619083	0.2840432121479353
0.8246868713917550	0.2960755742955925	0.8041914844671467
0.6678967195226306	0.7985290443185349	0.2869538679499897
0.1748450692945056	0.3036601498226972	0.8075278559100099
0.3134768450393324	0.2134628755299204	0.3089207107115897
0.8317945650877405	0.6995172705903414	0.7830580558757063
0.3204099751118576	0.8069937817103110	0.7014157091676836
0.8365346675972702	0.2987053428881055	0.2136429154178998
0.6768336471152665	0.2007822820476335	0.7214811090992512
0.1886519422230108	0.7095031743029310	0.1950594987754641
0.0839951629607540	0.1998533821349540	0.5068241712164863
0.5917343924536524	0.6933655641080065	0.9947234582266518
0.9195272036964113	0.7905942952897504	0.4930650234082492
0.4144680025031064	0.2946039673491256	0.0097970916976422
0.9180018900921709	0.1932689557668752	0.5083117783837565
0.0849296798983659	0.7961860450055696	0.4885581132600691
0.5830118278776553	0.2919484977109761	0.0093444542337338
0.2650610161971017	0.0067454817641490	0.8021727936342842
0.7778386556837305	0.4945702094356292	0.2923305762437565
0.7308927127939100	0.9984040944932033	0.7975659870535878
0.2272606322297506	0.5004258844236986	0.2934612133287615
0.7279262345748236	0.9965646949567954	0.2139194547086163
0.2262948371505047	0.5036186955733868	0.7067461613722159
0.2725787365523686	0.0082828189461779	0.2122720596437588
0.7749781295948992	0.4975132042778228	0.7051769789314122
0.1505468243646462	0.9965229654773680	0.5011998872036330
0.6573379449124417	0.4883704361276813	0.0022209989397410
0.8501860476789673	0.9912653871657184	0.5054701080590291
0.3462189420748274	0.4937705129561962	0.0056991282423624

Re6(H11C3O74Al1Si35):

0.5049294567026834	0.6629328623945199	0.7938142220789786
0.5094041040658013	0.6415885467328550	0.3695760625308679
0.5011419224790288	0.7444848613465140	0.5043720590303948
0.4271910294169246	0.6676184944375620	0.4780908408553017
0.5034109219893566	0.4931927752097138	0.7652107092584970
0.5123931843274363	0.4891677919346833	0.5279153095983702
0.4290726416691939	0.5201817247223488	0.6256784980263745
0.4893174297901908	0.1829378208308512	0.6292419294360414
0.4502618393090003	0.3296107432212665	0.5495215004634488
0.5070916903875897	0.3202435040949752	0.3632528398070676
0.5442745537573117	0.3305647639998242	0.5798422797331639
0.4846443170946344	0.6719271978830008	0.4876664649194211
0.4866788723449886	0.5243832229991270	0.6395925849695985
0.5004859597250686	0.3003849728818864	0.5021537314156177
0.3141048299128997	0.0910552500730191	0.7293439100981303
0.3941853251986061	0.2329047196771370	0.8302508239369502
0.8155871103627277	0.5901832512059926	0.2247118653493274
0.8723086083610312	0.7505121017334773	0.3285663174804228
0.6856248725686882	0.9058602742010819	0.7538320995326302
0.6346527638468515	0.7387684703541026	0.8311136735782796
0.1681409836318437	0.4161313196268281	0.2687324541898803
0.1027756991837592	0.2557641295563897	0.3257859192062895
0.6846030254209268	0.0933886136928948	0.2693606492860212
0.6244726992427161	0.2546574885525317	0.1822659697671938
0.1773744760802671	0.5906534894537856	0.7554119306337697
0.1027621134320640	0.7415545301558382	0.6724310897840411
0.3252901513372066	0.9200613198809720	0.2175481306576046
0.4021580482474576	0.7659578491429002	0.1895633534932983
0.8198599040197934	0.4053665939544899	0.7398977127496167
0.8751932894489229	0.2392544355478847	0.6746423200139446
0.6749355156440302	0.9109172370870340	0.2430952056441953
0.6007007038997898	0.7573387365764290	0.1750441057199126
0.1801175560006385	0.4078086825428429	0.7366166220940258
0.1285736509357278	0.2387930540965897	0.6740117816288418
0.3112405809743137	0.0987052475404937	0.2817824845992760
0.3777223990486505	0.2527453515308409	0.1845640172705103
0.8234917532296180	0.5883805262947490	0.7599719729866479
0.9001444170390371	0.7379988829833408	0.6764952088138259
0.3106599630462483	0.9094494824807740	0.7798927345173325
0.3745409797672394	0.7448140302980093	0.8108461089427550
0.8311901322494819	0.4090053045028981	0.2737499542744928
0.8985179402327574	0.2495550687191113	0.3253628517110414
0.6815969316251511	0.0886000992852871	0.7422803172249104
0.6083470325090730	0.2394896249188960	0.8284306542727862
0.1897398818815219	0.5958162842154806	0.2252257430767912
0.1274199253384722	0.7528082261059978	0.3223629957260954
0.3310065133630076	0.2356251397738589	0.5142575544123389
0.1024513479614768	0.0888730037068015	0.4774251792182227
0.8389709284740263	0.7253421110023766	0.9920666251559851
0.6131002814981201	0.5883912445623736	0.0295690263607824
0.6517928668974946	0.7865693299088719	0.4966283353726197
0.8999501339072182	0.9032091577313892	0.5138323670320233
0.1455220175211451	0.2967247344882722	0.0039750675009387
0.3973048461447419	0.4016189990365646	0.9805234506771967
0.6701756397105001	0.2278002049874388	0.5112058025253319
0.8994388930338246	0.0859746102431842	0.4888100241631435
0.1621570366774066	0.7273885969351639	0.9890175245055346
0.3937836696235948	0.5831737562257331	0.0300513767629127
0.3437525803537724	0.8112206430283209	0.4951996244569372
0.0969372700292368	0.9062956009285372	0.5052832111502710
0.8548282650482548	0.2934206057828206	0.0052992612956970
0.6010580292014680	0.4060950347431671	0.9861246166767685
0.2569907864353453	0.2569778588932508	0.8107896074650611
0.7629154479041347	0.7532339492249136	0.6978178905454797
0.7613972861962424	0.2475287248249955	0.2522544230653949
0.2628367308496991	0.7538136300887004	0.2271898427125407
0.7379510411486905	0.7440779005268183	0.2282652051808753
0.2401718634460579	0.2569293120050133	0.2533355631753267
0.2405904414014657	0.7543503837014143	0.6988628382295161
0.7457271491993183	0.2513888406677083	0.8048404143136949
0.0014632492388031	0.2093028392138976	0.5480088654391935
0.5069394681771371	0.6958512383253677	0.9318940145205090
0.0002257666684287	0.7785158745762288	0.4472916791418058
0.5005309853111228	0.2809338347798089	0.0315841439126601
0.1930021340751509	0.0003416257567679	0.6854973867172021
0.7066366239351893	0.4748210476088559	0.1741262944273885
0.8038832514768899	0.0011437849866809	0.6867950680148383
0.2954034846086415	0.4741569181827288	0.1748638868875290
0.7980814666956846	0.9870094360495614	0.3329236275567169
0.2982261056985749	0.5001501211046033	0.8229010411161539
0.2017715544297403	0.9846713661845712	0.3304812753835122
0.7021888248699781	0.4991023397716248	0.8203212297068063

0.2448079382841470	0.0229842949147994	0.0058097446997962
0.7524635736582326	0.5075185169357752	0.4976358259238793
0.7500221014777964	0.0045238668586407	0.0066171112197893
0.2498552476968072	0.5081309771537832	0.4977985723188212
0.5091896187237808	0.6208061555669602	0.6414731152063169
0.5008533045040835	0.2008947632642943	0.5083425822349383
0.4130773697397672	0.7000042336673715	0.9997953767328767
0.3233104662414448	0.2034454436153439	0.7195641834239979
0.8167343163535179	0.7026380633516638	0.1951185134619990
0.6840610016033324	0.7967146165257191	0.6959089783126728
0.1647684884019895	0.3062686599216704	0.2132502935212131
0.6846052564324125	0.2056632820267339	0.3032235838353969
0.1715948995716303	0.7034609074013503	0.7801300364207648
0.3341926306974727	0.8118885685047132	0.2815186989913343
0.8241851145219528	0.2971716091318868	0.8046886086208360
0.6675103799269166	0.8005055674881589	0.2866564988996956
0.1775986307287596	0.3004068074923012	0.8051772413422782
0.3153120323200937	0.2108620757643394	0.3070357766429268
0.8315856528120307	0.7008356714219630	0.7827564906467401
0.3183544225576469	0.8037557683206131	0.6997633665736629
0.8359146735520753	0.2995605211446986	0.2144373785894302
0.6763740998401815	0.2013167549416153	0.7195484502030851
0.1861455059136503	0.7079712405505063	0.1927220576352227
0.0846164650743901	0.1984673714280731	0.5058628509119174
0.5906047812039290	0.6961333446483735	0.9951927125876949
0.9181697363960666	0.7929261368792666	0.4921134636425819
0.4158528343620134	0.2923410148217158	0.0082046512003515
0.9186364873306445	0.1960334001969670	0.5089085632162025
0.0833799185415671	0.7946531809255077	0.4875765180508509
0.5842265557601820	0.2945925507996350	0.0079374464255153
0.2657505622005729	0.0046875957818457	0.7998092419205776
0.7768117776461381	0.4958470357493709	0.2925969243216429
0.7306118051742629	0.0001213887727474	0.7970873366522682
0.2260682687334068	0.4994943229506532	0.2911325311471487
0.7272838414097151	0.9996855694743729	0.2136236937107441
0.2266146237753262	0.5018486130036663	0.7047226137456590
0.2705735777484080	0.0063486539699795	0.2093254583232422
0.7746945232049356	0.4996881802524911	0.7053122519965243
0.1495614187198271	0.9952214818904892	0.4995974474224028
0.6566674478182719	0.4917669418664019	0.0019844092295287
0.8499899691233992	0.9945098358117477	0.5052501927781989
0.3460695049087548	0.4926152671943029	0.0035634102358415

TS1 ($H_{13}C_4O_{74}Al_1Si_{35}$):

0.5083114200024141	0.7496223302064351	0.7174439336403680
0.4951275212955863	0.8227132909042467	0.3628536386711048
0.5331896864751542	0.8938746308566650	0.5346300523616847
0.4436745437384246	0.8543635344617044	0.5521964328644984
0.5558564638544752	0.6286069884712049	0.4390227529439841
0.4578734802703650	0.6429490092592616	0.4328692372665444
0.5025342684005167	0.6033987088646811	0.6430169762513884
0.5542395415473180	0.3703683729510914	0.4496058661939202
0.5597219566176506	0.4435252833453589	0.6432248743662541
0.6051740125393152	0.4759244022092943	0.4429062559382047
0.3893500309761961	0.4962532918837681	0.4456083712288006
0.4282653886802734	0.3822873695324489	0.4524946208620833
0.4323164566542204	0.4563959445450152	0.6455707101262718
0.4966031800213315	0.8362001575447735	0.5057552163430401
0.5053411859583221	0.6227999228700725	0.5042393386835613
0.5580760061588634	0.4427158517832126	0.4973417707497845
0.4325365276231707	0.4546396031239865	0.4998452842696892
0.3291997964432340	0.0611613260432975	0.7628889837513810
0.4024379043726825	0.2139369785909295	0.8239008720990668
0.8242678619710659	0.5648349655679539	0.2568134691546931
0.8790289260880257	0.7296629965157138	0.3356992059903803
0.6900003971038871	0.8815336694340132	0.7242014159803034
0.6436234158733143	0.7165050570825500	0.8354580314869793
0.1808096291352115	0.3884193093817316	0.2320366941065259
0.1107673739209076	0.2357671772196284	0.3257935432289898
0.6855014079725308	0.0637443774068700	0.2409315610493152
0.6249684533436408	0.2275688138447052	0.1741111775874771
0.1720872230794157	0.5600544799168503	0.7279651149860058
0.1034869340585800	0.7175266363495325	0.6690452031689702
0.3226737109014621	0.8860767340538160	0.2551083860354311
0.3982416411728877	0.7335210387527908	0.1865217636668248
0.8232258858977701	0.3819152548649427	0.7698006442338325
0.8788443230943273	0.2194568845330522	0.6759481813125134
0.6850063315848587	0.8807317525473763	0.2638435578028009
0.6134576291089289	0.7260210743656614	0.1838349108105177
0.1834611102213728	0.3783460922367752	0.7595402424511306
0.1291267833476297	0.2138616572661221	0.6760496153731452
0.3180945619408959	0.0686448529672674	0.2365238915099326
0.3833157933294444	0.2283521212385651	0.1777275092721737
0.8331243586955850	0.5648868641362839	0.7362697595001890
0.9114603526236138	0.7161111504401774	0.6797639258896808
0.3090304169329343	0.8820885487179595	0.7123230537738223
0.3799797292942984	0.7299562588791346	0.8027251951734868
0.8251937848806961	0.3820328532923867	0.2438409922317675
0.8957900203807441	0.2277121684185914	0.3233343359137493
0.6792220663536668	0.0634208775497527	0.7694238663305626
0.6057806728753334	0.2179854639524947	0.8218144308221653
0.1872380290225308	0.5711309067510157	0.2616137281211834
0.1309239070370865	0.7366914656915853	0.3216771276876642
0.3527110367426900	0.1822108955231201	0.5033759128359421
0.0989283032325261	0.0672598414443399	0.4733806423260916
0.8628260210867253	0.6800974717969339	0.0006237764590864
0.6054866748409324	0.5650538268371577	0.0092775757461112
0.6787089926967127	0.7409579066839527	0.4947843891650763
0.8975554157563863	0.8825992402264475	0.5258630537745717
0.1655043162729299	0.2480507967065293	0.0056845676499506
0.3910697350344350	0.3792362718446896	0.9763763361375339
0.6522011138597890	0.1791938862283047	0.5025463851431624
0.9061461305096259	0.0653762540995686	0.4925306544321373
0.1448217590138015	0.6761456209465351	0.9933732891995959
0.4054453726864864	0.5610676584373167	0.9963421812500286
0.3244908953677097	0.7444868286188751	0.4823538352118106
0.1103350089981561	0.8857955193599025	0.5229898274956994
0.8386384314880004	0.2465111415453620	0.0061422522005827
0.6139983902234789	0.3822296518231454	0.9813680256732056
0.2649241789424082	0.2276838730040893	0.7647639209345414
0.7763560923287756	0.7362650768596737	0.7426947812988303
0.7573638093824542	0.2216028717952412	0.2895799970340676
0.2577954135408547	0.7264342627240126	0.1751889987313007
0.7516288309281904	0.7205835792033142	0.1945365040773908
0.2494859440304182	0.2290652525214369	0.2873723097847858
0.2397522670217924	0.7231602452613188	0.7523951790522219
0.7424862261337566	0.2306916585162142	0.7544166696592640
0.0035428138802445	0.1968398941046843	0.5344051037389761
0.5121331102749735	0.6999644256776278	0.9415614769330176
0.0053153588914086	0.7730412844785164	0.4453199632762477
0.5035027268423704	0.2743538405088444	0.0347128259469400
0.2023150362480663	0.0013517370689513	0.6703387395535358
0.7043107369573960	0.4731459427876672	0.1859699673814958
0.8027033872699363	0.9915466928483312	0.6779473207343827
0.3043560823442348	0.4748283251504546	0.1817323987671031
0.8048858460255772	0.9733453786903965	0.3241656853981496
0.2951918720124809	0.4866503557261197	0.8286192336283804
0.2011282492722941	0.9744541416287475	0.3162450149561380
0.7106244542887694	0.4890448067943893	0.8308743639217866
0.2508683789454267	0.9674229544290469	0.9924904764673244
0.7565450344645512	0.4679194677415950	0.5054333005251053
0.7535960405725817	0.9631729280277597	0.9998983156279166
0.2516934871557339	0.4647511449305881	0.5006310534081422
0.5225874397468360	0.7528800441781485	0.5910763427061738
0.4975803563199577	0.4941635047589656	0.4332655269129688
0.4215511621722925	0.6807215526567916	0.9861609403543596
0.3361526729066924	0.1709168284566260	0.7131988342764046
0.8297891735530385	0.6738558386119848	0.1980214499895041
0.6960901747659776	0.7684988891233431	0.7003333254128208
0.1768566519076984	0.2754263466489490	0.2124772743059680
0.6803038089732354	0.1727392488219692	0.2999642559740323
0.1661004707081872	0.6695955275842707	0.7852647600681522
0.3271300292976207	0.7720555655454575	0.2734484340505716
0.8210642209793519	0.2693799406641446	0.8001156143960202

0.6825146392755386 0.7676103062605932 0.2854181859966261
0.1858838591323817 0.2671132798736977 0.8001416415679117
0.3252070714143969 0.1764651535855322 0.2995280713219728
0.8454963829245798 0.6739676371332877 0.7901891820167748
0.3146058502578057 0.7683792069543927 0.6919779120383964
0.8291773127299109 0.2695086706329235 0.2151047088166322
0.6708556207365177 0.1723448443673092 0.7122984736031981
0.1808288889560572 0.6778259657598653 0.1890625550345675
0.0866182158717734 0.1785042356889193 0.5017937141473965
0.5923833264692249 0.6778862571279518 0.9923163124171026
0.9234028764412869 0.7755028044427377 0.4977515172761997
0.4189454376367223 0.2740412219404504 0.0028893053885284
0.9207515061847502 0.1770475465932790 0.5065385675587564
0.0886663974867616 0.7776113218239971 0.4902908956022074
0.5872291752683267 0.2750468858513244 0.0028503178721166
0.2726720777253036 0.9763115605468116 0.7853158883840763
0.7780654331568044 0.4722341353672732 0.2972492246183194
0.7317058656973003 0.9743509601993807 0.7926782119212987
0.2310158244091696 0.4753561085508480 0.2921270856692075
0.7326488525399100 0.9704224485113073 0.2079587433081400
0.2258819670295793 0.4727596471005242 0.7056923405825088
0.2731790635078752 0.9734591261673984 0.2004185299064503
0.7812139781928877 0.4758640540583912 0.7116897942856468
0.1540980619958603 0.9813820391217831 0.4953061716747058
0.6592271869930428 0.4775663083078854 0.0017570776744975
0.8525888149528384 0.9776169526628422 0.5045065532566042
0.3495648732616772 0.4784365899049945 0.9967701228470602

TS2 (H11C3O74Al1Si35):

0.5159724309764826 0.6545544384007798 0.7220592285647243
0.5009201768207348 0.6424970099233605 0.3411678740203143
0.4801148873250298 0.7418354549610200 0.4733363350204769
0.4280117893580595 0.6383426371193437 0.4925036159267364
0.4963948822521473 0.4873110639263416 0.6978458630571609
0.5911831320532102 0.4975534719924255 0.6320895416712702
0.5208482023362748 0.4971649788697832 0.4576041768501042
0.5846937793805580 0.3356608554274487 0.5437120984408566
0.4853195954351222 0.2481778317739938 0.4787102663882123
0.4876285798131974 0.3502019197265085 0.3398022554439493
0.4336075746720525 0.3481260572435545 0.5352741604384903
0.4814023740949835 0.6654552457517013 0.4706769220514175
0.5362791527613595 0.4888962645573969 0.5953671933708193
0.4828849117979033 0.3244773127305862 0.4759538079162695
0.3119772573843704 0.0807262931698176 0.7158758102302656
0.3953765320427749 0.2195001891092545 0.8221558995321914
0.8093824156144009 0.5870336371714444 0.2306264844377850
0.8714871248387439 0.7405782808079024 0.3448878702229266
0.6851570478993807 0.9050527367997176 0.7759384103044269
0.6375090085311186 0.7361119156715930 0.8487810155474378
0.1717742748077100 0.4015331530736646 0.2587847703228050
0.1039926809875382 0.2422977183661246 0.3132548870516985
0.6792168279254938 0.0946092003786276 0.2820628731655788
0.6177797317540126 0.2571649093583162 0.1993619390356756
0.1724179632489538 0.5819665006097452 0.7379981374244338
0.1030398422230121 0.7381814656341490 0.6697586372097248
0.3215250136200751 0.9073370003064412 0.2060220812675837
0.3954730141877931 0.7510415762214961 0.1818601927064805
0.8243969446847430 0.4067102106941149 0.7543329757410362
0.8824699253410202 0.2411316038139475 0.6928407520229243
0.6736884019449292 0.9116994967667793 0.2526925725927072
0.6011736546968578 0.7560853839835460 0.1888156280745861
0.1780491161923266 0.3989948709718831 0.7300725528679521
0.1314912033468900 0.2282673252676162 0.6613174208965447
0.3200754581123206 0.0875730388937939 0.2612707430300389
0.3789846323150385 0.2481932850040494 0.1755852472361710
0.8242308156380318 0.5898416602296095 0.7683262723217794
0.9003913629993363 0.7411204093787518 0.6929274642504453
0.3089298128281683 0.8995636871713160 0.7754135006949738
0.3763906309196798 0.7367067100996181 0.8025152745163595
0.8298760228961655 0.4070223519741276 0.2877651848072205
0.8930190128262012 0.2444491615183679 0.3388376562638342
0.6866430078606811 0.0870816831499184 0.7370939092485500
0.6130534249526960 0.2336405605337291 0.8474938419056741
0.1897478990686992 0.5813487897040091 0.2129776508945369
0.1201268971227165 0.7315782416137111 0.3157181804546951
0.3367532045508526 0.2220831961723277 0.5017963197586468
0.0977606665543433 0.0775734716751870 0.4759721598786655
0.8370956254381241 0.7243053077929531 0.0058182925046992
0.6158598443297691 0.5875267204146652 0.0467796186612759
0.6513761777362248 0.7909639599814469 0.5117635923741517
0.8981402405967955 0.9004969470100832 0.5088479312419523
0.1489765052933905 0.2825721072343654 0.9933803892831037
0.3934380136773896 0.3917880385468280 0.9539291363355460
0.6595227043719447 0.2301808588452232 0.5215194508549175
0.8987626528653010 0.0838634970044083 0.5104329220250250
0.1607334827484408 0.7147134719781008 0.9837914904180565
0.3959975941208214 0.5724138774035481 0.0141325320665970
0.3416599067108450 0.8042741458209406 0.4890281886021057
0.1013889515430435 0.8951262030596823 0.4782031464261678
0.8499313899495391 0.2897741829163820 0.0195096800723533
0.6009481229332678 0.4047788774220606 0.9904752841107509
0.2598556784252679 0.2510791193046629 0.7922493794150967
0.7632699487338428 0.7547160165354541 0.7072172890371888
0.7559760118983476 0.2491963304178668 0.2683623319516570
0.2560061683476889 0.7447030234204703 0.2351748595109697
0.7380162143066400 0.7464415195329082 0.2464530841107759
0.2412305882956360 0.2403071977610267 0.2475411732393411
0.2414733760144756 0.7424303583760619 0.6977665088674954
0.7489027005961120 0.2523390754504078 0.7907142159641580
0.0027621786494407 0.2068242953516872 0.5409953399208263
0.5084058807769978 0.6925570817135807 0.9444307575812942
0.9984197798362473 0.7748095813844174 0.4530922812011937
0.4994980303937311 0.2789857026236220 0.0244834829050760
0.1906398316266475 0.9896983862767073 0.6799288093576834
0.7031124717618980 0.4666852525268226 0.1943876874673123
0.8064650341302055 0.9926228041005487 0.7062574351363509
0.2988781611841986 0.4642243404689381 0.1703329528894528
0.7942114511968867 0.9933010000440774 0.3512131504485723
0.2930775763598064 0.4961907709505056 0.8205219349943604
0.2041479261476979 0.9890305405791153 0.3258178745013041
0.7054058126012634 0.4956850289032104 0.8399821758107322
0.2443758455589418 0.0164779621846591 0.9969293084600821
0.7516122947825608 0.5040186674043383 0.5140117055468778
0.7480435033056239 0.0111873319888147 0.0210488931281034
0.2493754285837966 0.4966945415626469 0.4906116989657860
0.5303191845774364 0.6314940784794345 0.6021242318392979
0.5395176201124698 0.3611673160115458 0.5860410924748010
0.4166356754759875 0.6904065292943216 0.9896387845498253
0.3249046131080675 0.1921882620137509 0.7077239169867156
0.8142166687214285 0.7000152523798988 0.2078725797425207
0.6834628966061373 0.7962447964787529 0.7116205322855294
0.1669799770971991 0.2920914210674042 0.2031099135605245
0.6792845491988615 0.2065810090238571 0.3140870031430083
0.1707078400680402 0.6943928799905450 0.7738572837534221
0.3299959537659021 0.7997909003616144 0.2760800415753479
0.8271172502652391 0.2976016619185984 0.8132782994374637
0.6662117488770889 0.8010781590912401 0.3006173863277724
0.1793120102757442 0.2908325890404626 0.7933661185456344
0.3183565974171998 0.1991500277783231 0.2948784369607154
0.8311846591265990 0.7024623439755615 0.7950925229243708
0.3186520997933542 0.7938535408705576 0.6948265725321789
0.8321481094190020 0.2975802080025218 0.2285938903236513
0.6781715466065847 0.1992808034831626 0.7260762792386828
0.1825925774441330 0.6938191975314609 0.1882192893933166
0.0849949339828555 0.1888091483096233 0.4970484976521732
0.5887235767682739 0.6946253650250966 0.0078145828671978
0.9170905150562376 0.7897872792566432 0.5013149344323509
0.4147939320854093 0.2848440355982049 0.9932692642831071
0.9191750026485366 0.1936947134762690 0.5204515393151112
0.0827238490867472 0.7847176873286941 0.4798378709114672
0.5831111601093966 0.2940815956650482 0.0136958408312609
0.2641642531477117 0.9946962844389660 0.7914864596956122
0.7740459924515122 0.4925789269627074 0.3071508514481707
0.7319248735154744 0.9980402852589592 0.8107222773009752

0.2278866141922293 0.4871267843697993 0.2826077849401472
0.7243546495649866 0.0017867762140895 0.2273386752273936
0.2235727436963231 0.4938931606412638 0.6960243707835048
0.2723246359374301 0.9986016148870362 0.1982963177163626
0.7767943798131469 0.4993400470308345 0.7205846076888420
0.1496141198611411 0.9876669793669777 0.4903246612734904
0.6567517943586694 0.4902895990540159 0.0176237239164294
0.8490543470304317 0.9922888838141830 0.5185345938364563
0.3454176981305395 0.4851073337432936 0.9916002340833288

TS3 (H9C2O74Al1Si35):
0.5049760847699289 0.6242036959011799 0.7390613217506257
0.5017634879312425 0.6237384424170074 0.3564964996533675
0.5000252255937028 0.7238782569794182 0.4976803007808712
0.4253850346843819 0.6477312115976659 0.4889359901280059
0.4340617128798101 0.4757598701792531 0.5925742858807368
0.5193463227127708 0.4637103938856981 0.4684151839420423
0.5174268546542820 0.4436347949914463 0.7122352988937095
0.4434386227878804 0.3207236409812495 0.4456117305468368
0.4501030887182225 0.2998890867844040 0.6480279276961696
0.4831179230895941 0.6510709217228268 0.4838146286491281
0.4892724588112785 0.4561470885435526 0.5892147772803327
0.4752085420358938 0.3297105286417903 0.5469562444301701
0.5150486641728236 0.5950620828937829 0.6213026376328230
0.3225157041951690 0.0937742900564231 0.7407775507737000
0.3965451626361656 0.2471263753972224 0.8179552290618304
0.8160904757295455 0.5875454032520651 0.2251776639028193
0.8767333068121772 0.7459142662323828 0.3257170188500851
0.6840643003531923 0.9058356650203265 0.7545290702051872
0.6321390706983578 0.7385152059992990 0.8303805814223253
0.1633914945502184 0.4118372314982395 0.2700437313519259
0.1020906105633230 0.2477325325361832 0.3205491583174762
0.6851124400770985 0.0915638540524029 0.2674901592133452
0.6252076570668663 0.2522069540478569 0.1785115060963989
0.1870758036892539 0.5903408465537794 0.7605195963085620
0.1054434775115496 0.7335397482220998 0.6726436641287989
0.3188249489737842 0.9155792147872504 0.2140605696109006
0.4011267750481551 0.7663187721928750 0.1864986478260207
0.8220878193505499 0.4040041523030808 0.7426071411632691
0.8727564692621357 0.2359710531228814 0.6713248919737325
0.6768930161524978 0.9092334839093894 0.2411583714705117
0.6033161110776817 0.7551179305199724 0.1744996544096722
0.1797438820289372 0.4077702902855620 0.7394726044886681
0.1290022552070980 0.2388920644886738 0.6685651773791150
0.3080330616080076 0.0944722585364062 0.2842353846524759
0.3770152001980946 0.2456833130158245 0.1784426232215495
0.8202607137708497 0.5869535932022885 0.7587689879204902
0.8985930285323960 0.7344699933747023 0.6768111537966206
0.3152564744764277 0.9116612124495660 0.7798679130864841
0.3760187549547283 0.7444220334570275 0.8100430335006052
0.8343263218530907 0.4063163754073429 0.2689971130803741
0.8987361143059118 0.2446694624883179 0.3222900592486369
0.6794136450677613 0.0888322659098506 0.7455962953589506
0.6051869800850795 0.2406706805337180 0.8248904558904684
0.1838466357374671 0.5928854016900473 0.2310137865629116
0.1258654426499248 0.7537354331743344 0.3212268101167552
0.3363581101795958 0.2326116095364625 0.5079181376828501
0.0985439988313459 0.0848223424954426 0.4871297795384919
0.8383858513192450 0.7225916620351853 0.9926841529189758
0.6188638607438506 0.5881770459349269 0.0309313988752180
0.6514580883313101 0.7874572294367539 0.4968319806178627
0.8972917670864504 0.8997833360241074 0.5160136885338815
0.1473199002331995 0.2918221096294644 0.0016049549715476
0.4000804684083974 0.4044391205700535 0.0040909892395078
0.6634704544959789 0.2237792037811275 0.5084523901920477
0.9017973151830532 0.0822760969526684 0.4913066303537619
0.1645126607116154 0.7256134592532391 0.9914851013920156
0.3855592465916828 0.5860882205798887 0.0346814333191503
0.3451046019877779 0.8133439640776672 0.4949739225475511
0.1020646777810796 0.9024282941791242 0.5239019899973414
0.8516467764973754 0.2860788416418316 0.0038528027610667
0.5994540871171381 0.4060484583603667 0.9888706448031499
0.2577278066395436 0.2568438222487046 0.8007743384494788
0.7614930012230872 0.7537290970294066 0.7012344202795475
0.7611244814957274 0.2473988446557485 0.2632466392591368
0.2625709081743892 0.7461313203950297 0.2403888720386007
0.7408995756888501 0.7436786304849718 0.2382357922169618
0.2399116892731499 0.2564869338966247 0.2576164866512087
0.2416678745970415 0.7599449439244736 0.7020522794595152
0.7427943767989760 0.2534795131454857 0.7960398003772795
0.0005669525843288 0.2103718951208791 0.5503654024458413
0.5066109829989003 0.6868809879494364 0.9454824189362370
0.0012800059646025 0.7802101930258090 0.4593329846182499
0.5005404766814365 0.2794454804025293 0.0399563222217338
0.2018046041538213 0.0078894955883371 0.6689967096380656
0.7094605521151607 0.4683006559037821 0.1679113764298847
0.8013372499291478 0.0026490327953184 0.6808020515603914
0.2873324546284479 0.4700116259948075 0.1605550920569598
0.8001153368075222 0.9879480939098002 0.3271006040667999
0.3042025281094922 0.4876824644164017 0.8091502725459492
0.1946578824935372 0.9860758885521435 0.3158722425541214
0.7011549652988975 0.4912946284634430 0.8142626017806904
0.2447827445643895 0.0247663924120047 0.9989199810839793
0.7535783803777676 0.5008820988920917 0.4941567018415398
0.7506993378097491 0.0047668452057508 0.0034827005381857
0.2494415121317957 0.5017358329146404 0.4915962835206997
0.4163636979692100 0.6991724676386113 0.9980667082120007
0.3257405757421949 0.2054422710845003 0.7164467961638366
0.8180378085736066 0.7002144658659191 0.1970760141951260
0.6819223215431620 0.7960806140488330 0.6976480489499224
0.1631247111213412 0.3023118435177565 0.2123171411557578
0.6842242508909493 0.2034055601683704 0.3032967686476837
0.1757342445747341 0.7026127746133923 0.7828158518205797
0.3333880507711129 0.8085602290534055 0.2821550377978284
0.8226650578783605 0.2949383763087638 0.8022310617976487
0.6685573619534253 0.7987135638723188 0.2872237339118300
0.1776570956762740 0.2994322314300391 0.8016501941395546
0.3137389178716106 0.2062825892111633 0.3037140310404141
0.8294207952709201 0.6991940234509215 0.7834189117988259
0.3212409559395297 0.8050441941399953 0.7003191356289591
0.8359866235620359 0.2961918149535165 0.2141155021716870
0.6733690382906303 0.2009442048517940 0.7175964814172531
0.1851104752667894 0.7054345662300463 0.1972718848374342
0.0830487662711334 0.1955405185880608 0.5061909727383096
0.5884669025197979 0.6935541638819700 0.9951292261613389
0.9181006633762223 0.7902850288979195 0.4948222244806857
0.4168622212445854 0.2944834842694988 0.0122458254483817
0.9180821271098978 0.1931957188801121 0.5085555810792641
0.0848980624920870 0.7921088237612324 0.4946315999439932
0.5842758363711056 0.2943826199192188 0.0081568379650108
0.2708110553780827 0.0067675280652324 0.7963866225681429
0.7784601452376592 0.4918570806981251 0.2891203834473828
0.7291016481650630 0.9998064094939849 0.7953489903742366
0.2212746254375944 0.4959752823352304 0.2876598762527820
0.7283969755048503 0.9981225658862698 0.2104804821497055
0.2301090670788852 0.4979940527190081 0.7013347504749987
0.2664471854187269 0.0034621562091317 0.2037636951224471
0.7743611444929996 0.4960096727126171 0.7029988305676298
0.1500005433460885 0.9946021338467332 0.4989729043293423
0.6578407527836063 0.4898135241118098 0.0000594826657121
0.8496345502142404 0.9929274755905979 0.5037591396182890
0.3440199675211275 0.4921468868876602 0.0031555370409606

TS4 (H7C3O74Al1Si35):
0.5060489401466484 0.6381007100257676 0.7419819384452353
0.4998500192709727 0.6314293122467021 0.3570475535596032
0.4959736415901830 0.7334665798570299 0.4931993085823194
0.4237207944687640 0.6533781378038626 0.4924915371196548
0.4359927868163069 0.4870577341612261 0.6030162637212371
0.5247963049921083 0.4759101902504597 0.4922503598659844
0.5165149532910078 0.4622695138715400 0.7397885688601136
0.4811681385400490 0.6597182155086614 0.4835481620180317
0.4921787949299556 0.4725095246699524 0.6105066635455429
0.4831140051750893 0.3421150399158392 0.5635409446415593

0.4981761494250421	0.2775002979539849	0.4844320632805363
0.5160249503275836	0.6081005447958603	0.6232199683630668
0.3219018741176995	0.0932015997135167	0.7366341963766274
0.3968417498310117	0.2431997939263866	0.8244889762004206
0.8164168580132412	0.5889711908179862	0.2291173724178890
0.8754196137078196	0.7483935045883996	0.3301412014155147
0.6840822253608252	0.9077769584768589	0.7550240140081002
0.6345526437166527	0.7398041404499480	0.8352570603996128
0.1644203752908169	0.4155674932226106	0.2682714057232545
0.1025523054277642	0.2522081963142710	0.3229590849833599
0.6851669466842338	0.0928197810950095	0.2694613711214942
0.6264310706571905	0.2542971120661619	0.1791026922412300
0.1855398868981410	0.5907364183920478	0.7588474785383426
0.1052841338310202	0.7354796217069008	0.6698456046621644
0.3218166120155246	0.9193393854474365	0.2161238403531058
0.4012771884309956	0.7675286756554769	0.1858667283803399
0.8220264894194926	0.4061820553512518	0.7474178193597112
0.8743168406512538	0.2390904834599610	0.6736507356242640
0.6772911895694378	0.9104318453016731	0.2458225516732639
0.6027963977028250	0.7570764173108557	0.1781541244484401
0.1820168290417001	0.4081068007137674	0.7390805169237069
0.1298577871277757	0.2399302714650133	0.6709375959002841
0.3076659855822257	0.0976981599664839	0.2856867885214029
0.3770800419339508	0.2479145900264541	0.1802761057090123
0.8206008593709271	0.5891826959507114	0.7599184606281923
0.8999103155192074	0.7362617860643184	0.6798519721947613
0.3146152231866637	0.9113113866377205	0.7801919596349975
0.3760390081767468	0.7444181593602863	0.8084330063409482
0.8339876294473285	0.4076609282012456	0.2713176664112211
0.8996267927416568	0.2467500353904768	0.3247711473442791
0.6792360192143008	0.0906997528819318	0.7494272458371114
0.6058827453983682	0.2441596654553422	0.8251048221808901
0.1857767275931153	0.5962120592820810	0.2312024444909042
0.1257048834042180	0.7560867766587975	0.3186545492745481
0.3359214831062616	0.2347817133669778	0.5113771334988186
0.1005865483182475	0.0873876563341156	0.4834690674844140
0.8393647796929642	0.7235427716209273	0.9957280967612547
0.6175168229996544	0.5898167242356251	0.0341805632403762
0.6523137375157901	0.7873765527268333	0.4999592244388751
0.8974375975552036	0.9018114010461460	0.5200498997398029
0.1478377603062100	0.2941822007043413	0.0029646891559807
0.4003568009498863	0.4046960012487943	0.9988547191221215
0.6632498869108545	0.2251457611306833	0.5099413204407668
0.9017733230391656	0.0845377077139275	0.4942930034603847
0.1637119687101105	0.7267806624767488	0.9889476646835931
0.3894371583106349	0.5861087405674041	0.0338709251578209
0.3450681900127606	0.8133710341990152	0.4940881492133045
0.1009546126332381	0.9048323087856360	0.5207662759293115
0.8517236521238871	0.2872542425506808	0.0066209388833371
0.5995992885629278	0.4081757479199410	0.9908877901609401
0.2585626512413413	0.2562392255728838	0.8052092746649464
0.7631051163932554	0.7568628108752904	0.7038404335290593
0.7619526755809503	0.2480290997756072	0.2671920743793805
0.2622390035523452	0.7515407789222621	0.2346013446069790
0.7402906486715466	0.7441427601922754	0.2372621352994599
0.2404499705725485	0.2599252639643339	0.2604105461160628
0.2417580724664816	0.7591041518923447	0.6999847735497866
0.7438044065298115	0.2545937247333541	0.7959060419015103
0.0017759684894756	0.2110810948205355	0.5517441304166866
0.5073801904357538	0.6916608101791226	0.9426423773478447
0.0010368435945622	0.7818568663497203	0.4571755414980601
0.5010669019623819	0.2811498622202677	0.0423084345185458
0.2006265290939453	0.0068753957979482	0.6723572813791066
0.7090717843600487	0.4709099298129189	0.1717668047275893
0.8012710095352702	0.0051069965744546	0.6832745888745393
0.2892042246120639	0.4734364564544506	0.1611304986023256
0.8004661625245078	0.9897295854258559	0.3297490279707134
0.3049033780211090	0.4927071991543244	0.8095249603755121
0.1965452572036881	0.9855436838715761	0.3179231896539605
0.7012299258125267	0.4945441674980700	0.8178508195333052
0.2456925960623551	0.0254168189620358	0.0000299329180820
0.7533809141355476	0.5018884209514951	0.4975358728624641
0.7505500084527128	0.0053784521016240	0.0061028553721343
0.2505688132720252	0.5043289046464210	0.4916868444801921
0.4165386372431925	0.7007129429995516	0.9974066235626040
0.3268762172714474	0.2055183364784128	0.7186108813469119
0.8180640421226357	0.7015926630067923	0.1996420910914409
0.6831595545627422	0.7977510494516906	0.7002489730770094
0.1640423997877463	0.3055141304911544	0.2134362705655164
0.6847288611919282	0.2046407545124325	0.3048937781559928
0.1750978932259528	0.7031808063069391	0.7805840559116675
0.3338023035046547	0.8114095829497998	0.2808572142393402
0.8234841692183679	0.2967314802967636	0.8045410650492855
0.6687055025791722	0.7998096495312765	0.2901418782446825
0.1791944968782531	0.3000506248247419	0.8035868076553783
0.3144470222208824	0.2095883206717275	0.3069635146653837
0.8306022776763626	0.7012328407570507	0.7859930947995650
0.3209008800148325	0.8052464198525868	0.6993529526814513
0.8365164169097667	0.2974757215324999	0.2169858047407374
0.6739271280013881	0.2026625397464059	0.7193705778551234
0.1852548215730110	0.7083816354808192	0.1947176785355040
0.0845107991818779	0.1977692083614997	0.5067385144140459
0.5889706895563833	0.6961094025634580	0.9977085066584337
0.9183350515276416	0.7923708275205854	0.4972190400966312
0.4168352581010097	0.2943578686506712	0.0124469380562076
0.9192611004218634	0.1951986123271894	0.5109476946317670
0.0847707716924475	0.7942932593857464	0.4921491164467184
0.5842526861048825	0.2962621880283081	0.0100584504615640
0.2706458702001555	0.0071290102390691	0.7966680613336692
0.7784946587376353	0.4934226845309055	0.2924619964167405
0.7291140358196202	0.0015561274894367	0.7977956797502515
0.2227788225479514	0.4988977086675703	0.2874012253271871
0.7286608676254573	0.9993275516821996	0.2134424304303835
0.2307244861260713	0.4997148343268876	0.7011006632066383
0.2678510983421987	0.0058788872089011	0.2051704588500343
0.7745546705948456	0.4981290197306762	0.7063443221924501
0.1506143236364892	0.9958288129359332	0.4985977096677843
0.6577278380760845	0.4921596885727979	0.0034389484853534
0.8499076403918053	0.9950232630317615	0.5067284343452556
0.3456516290869374	0.4935320571205679	0.0024499699197307

TS5 (H13C4O74Al1Si35):

0.5004027707165051	0.6112710570986550	0.7522180605271487
0.4907924930783949	0.6045964918117761	0.3701527461284400
0.4906352414200796	0.7070635513153931	0.5060382637880778
0.4166549804499695	0.6297292005564756	0.5098855413571641
0.5023852921294534	0.4276916264595343	0.7489886101214793
0.5310675269767060	0.4430125451997355	0.5132406260568700
0.4348345345791103	0.4497902923851953	0.5715660393378172
0.4819970266568632	0.3346296833514657	0.5791172314443713
0.4845861756278822	0.3092163256947558	0.3452065861349993
0.5576957855773017	0.2730546523401358	0.4909222531454418
0.4619913467284675	0.0604747484592068	0.5795453886419253
0.5499191821765663	0.1020750064972162	0.5414893660096638
0.5008347696120410	0.1423544569111144	0.7313163469553814
0.4742099974056586	0.6337618050805958	0.4978110506322508
0.4891083556514602	0.4349580875165842	0.6093046733490723
0.5007207848942272	0.2749168409949547	0.4678776206145514
0.4975244699259278	0.1202956202461181	0.5923530902217280
0.3141759642479087	0.1023422697090339	0.7256608254399950
0.3897509427103500	0.2497022204037194	0.8175412368683013
0.8160064336607036	0.5880026379816670	0.2214002668707948
0.8778609594503521	0.7448387201046600	0.3259646027008500
0.6821058876573360	0.9088568779552091	0.7558548347679377
0.6317612080698396	0.7404046840488334	0.8309159963909210
0.1664570063600976	0.4160084403750588	0.2738986412426314
0.1009650254861256	0.2541446921631803	0.3157042853594589
0.6857303464239806	0.0915878410405639	0.2712815854512201
0.6222057448083547	0.2488880982580923	0.1778960642628533
0.1895967988259173	0.5987273513984590	0.7599539610575263
0.1081980534813169	0.7431728510107618	0.6764982218732172
0.3238861931444035	0.9214462613349852	0.2130481066594498
0.4031019138691008	0.7685603918839945	0.1911512404515676
0.8239499177852352	0.4071460624035907	0.7339433336708510

0.8748425449529478	0.2379759182171099	0.6686317360070433
0.6763489179442246	0.9093657245133357	0.2374878319266287
0.6038855917117374	0.7537628125970670	0.1757031289624820
0.1766524101206102	0.4165866941059164	0.7304393550003070
0.1259497406641827	0.2468198750429167	0.6650039903391516
0.3112366030309914	0.1009449725162526	0.2864528826265840
0.3756100517262482	0.2538883269738434	0.1766508505728126
0.8168130211143989	0.5898623730516164	0.7622503434160990
0.8978221998588083	0.7347591060755008	0.6772564787097224
0.3180600315219309	0.9211114436208234	0.7782407746055000
0.3785013600947380	0.7539519481980150	0.8145498145729846
0.8336012628205083	0.4067625326825279	0.2742584475189318
0.8968512220022973	0.2433397859322213	0.3185140381255636
0.6823250044061950	0.0918529892605022	0.7408851077570731
0.6093145287767996	0.2441890692930073	0.8206145744277009
0.1894508176993241	0.5965924485405623	0.2214837428069742
0.1268520619782478	0.7524272519876689	0.3233568456952796
0.3221022416615682	0.2469294625112184	0.5046424253381545
0.1034205243105788	0.0906044103361552	0.4813291663890453
0.8371954756525568	0.7256579409221260	0.9935466950925402
0.6202833116375501	0.5884252027479349	0.0263861827606391
0.6505373706296140	0.7904334169603402	0.4976872253115872
0.8985315595420005	0.8994523298028483	0.5138280609026324
0.1397015835464188	0.3060068699927569	0.9961005724701266
0.4037062280458628	0.4094658085058640	0.9973331367829985
0.6698077931322999	0.2281129794321259	0.5051897608709908
0.9003799429372634	0.0824415274184958	0.4905438062910434
0.1684958622701487	0.7336381068969331	0.9931050364860496
0.3839537358862302	0.5907871240404248	0.0297764222651651
0.3492142777980831	0.8209620192991309	0.4975077462121646
0.0994664639656762	0.9073380701538980	0.5072466305579155
0.8561745176276787	0.2938547445624593	0.9999694743172753
0.5987141704788428	0.4058511038030588	0.9944877824003768
0.2523819694090719	0.2650230147071468	0.8080357525139306
0.7610970007314464	0.7582902928134132	0.7019232609923733
0.7603887540320073	0.2485569669954444	0.2410332997419502
0.2640338546108794	0.7539590309598339	0.2482469315957871
0.7414604320073650	0.7444551647337718	0.2442639433984866
0.2369110466815130	0.2554748412822150	0.2297053217868097
0.2446414415964071	0.7682570558809019	0.7024495523489094
0.7467076779114947	0.2559310111126365	0.7983823324015518
0.0006464548717346	0.2090034822627658	0.5415730107411036
0.5067141271165099	0.6860850098156916	0.9475856280308932
0.0022014578232543	0.7785013745858578	0.4623180753881542
0.4992899341533672	0.2771931164829695	0.0233437349102701
0.1970653977316132	0.0032561652980334	0.6801795010432770
0.7095679028972128	0.4681582592042588	0.1674258225478482
0.8020096275444573	0.9997949373187325	0.6813016513743122
0.2914088338566572	0.4700407440834624	0.1646527773287616
0.7998155302888463	0.9860166688806515	0.3272502252857450
0.3026075097024545	0.4888181182213316	0.8118059279924381
0.2005982661610162	0.9882246500305243	0.3232331792299732
0.7008569975552490	0.4865795900294145	0.8130035402472444
0.2473090324971494	0.0303898735970378	0.0024171622961973
0.7525341701892998	0.5048420599820105	0.4929650728802102
0.7503739512027039	0.0072798552266917	0.0034325425851733
0.2514843250853218	0.5100352237386616	0.4916508733798111
0.5092992387657418	0.5807759811909250	0.6351521711101711
0.4664509407451760	0.1928218548625225	0.4844895093969868
0.4166921861836101	0.7031753445616715	0.9998334926034573
0.3188091442733167	0.2150665698970684	0.7117698033907942
0.8182185933192656	0.7010822251002722	0.1978864279861554
0.6809852639309923	0.7988336336647706	0.6984936451154837
0.1616164147162920	0.3083161880396759	0.2045966598472546
0.6855424338502729	0.2041757386862884	0.2967750180218118
0.1788517788110511	0.7109185843428705	0.7839924756749141
0.3362364296363722	0.8144845101969622	0.2852033453265292
0.8262854234214174	0.2990540889908715	0.7988917608516743
0.6684279724405968	0.7990570046354009	0.2880400680247064
0.1732519565954433	0.3091167758377011	0.7986525613278133
0.3114899404158393	0.2140054448571647	0.2986501588977220
0.8281779148289630	0.7019228428111788	0.7844037234152950
0.3240519212333481	0.8137989192970423	0.7021612874362546
0.8366346499185227	0.2983673457211182	0.2087742851079142
0.6782625035668914	0.2042442291849440	0.7159487054473601
0.1882684533765300	0.7098570649692491	0.1980464237106787
0.0838838469335101	0.2000957493036566	0.5001059671343313
0.5885381818660420	0.6935208341348797	0.9948152722202934
0.9190792740173066	0.7895906215958917	0.4951824692318993
0.4145852519342128	0.2975592860757388	0.0043460164229714
0.9182302162982126	0.1929948450862540	0.5044079604177156
0.0858665207604687	0.7953448480196670	0.4928243170412188
0.5833428000080616	0.2939964495692138	0.0048729396152751
0.2690303645001961	0.0125606415909374	0.7965154366232312
0.7781718841216474	0.4931881031625736	0.2888773475694535
0.7296795262196198	0.0008573614279186	0.7948459444946110
0.2248260683094472	0.4996922363565730	0.2872864286287395
0.7284438634397077	0.9979219330501721	0.2103733746560295
0.2301304994427582	0.5044288695407531	0.6999723593307721
0.2707500710389965	0.0091651940705546	0.2064235012368840
0.7736816033871179	0.4976360761734071	0.7014077649163255
0.1512035804595797	0.9970969338802149	0.4980678105167371
0.6578164713248427	0.4889827334399541	0.9994466936286983
0.8499512189921106	0.9916207059970432	0.5030868433255264
0.3451298762029467	0.4944327981387175	0.0017302391136198

TS6 (H11C3O74Al1Si35):

0.4998250188876341	0.6295055084284726	0.7386169009195243
0.4901381815359827	0.6201668058295411	0.3559586308372218
0.4890908642661032	0.7240347259756135	0.4875392513712882
0.4160988289509362	0.6452565779639627	0.4956963395376448
0.5152966215510304	0.4462705069519117	0.7221390796353120
0.5167243537999013	0.4663551916553530	0.4768684357742288
0.4305876868212550	0.4679877297568993	0.5992909390686449
0.4792044400854536	0.3583246130148510	0.5687066136713881
0.4702020223232424	0.2921807562333356	0.3661726672941299
0.5581471290344382	0.3097310783141793	0.4676450041461422
0.5276466365159038	0.2033941098011169	0.6700877275635406
0.4735324788034134	0.6503765407174953	0.4828096931629062
0.4870634475344815	0.4541742723237476	0.5972822982440888
0.5029976741316560	0.2893030832538912	0.4853933803317645
0.3173968840620063	0.0968136804929642	0.7310269299412866
0.3877626792482758	0.2481854619846118	0.8301400456971919
0.8170719309202283	0.5835482706176336	0.2182318708680242
0.8786388588534209	0.7406082102532991	0.3232045531379057
0.6836425551412546	0.9025661190434420	0.7528933467863510
0.6341582868845279	0.7338780180449831	0.8304739107093795
0.1642232520903733	0.4174609625677448	0.2767594885802538
0.1011775031456565	0.2539348029356532	0.3226332545333932
0.6850961148553338	0.0863660591917395	0.2659177357404587
0.6222458091146308	0.2442419603604051	0.1726635508090979
0.1877894165754199	0.5949914377066605	0.7619354239589811
0.1074329900257993	0.7401343557337015	0.6735022421427459
0.3214941598136818	0.9222610918034349	0.2162508944508161
0.4030820404558710	0.7721112413821274	0.1864559826786092
0.8221142614109027	0.4019930899847566	0.7344393895253205
0.8691767222846778	0.2317753170402526	0.6626723308971734
0.6770293069287234	0.9040389467306085	0.2370068504092799
0.6048909968403819	0.7482271389088453	0.1744572836675289
0.1796825787329368	0.4127515626602616	0.7358637377461292
0.1218132989300358	0.2471736527299413	0.6736198478753934
0.3084744954536589	0.1012830620189220	0.2847159560088031
0.3772416972773271	0.2538436656649949	0.1875255536595972
0.8204795338132840	0.5848779975606959	0.7552956867019347
0.8999137400706019	0.7312966298900676	0.6742841696343547
0.3155517884682695	0.9154118972899923	0.7831152821276390
0.3774103482680431	0.7487545475206261	0.8101432579141985
0.8328454014221691	0.4022246538198857	0.2660034426283246
0.8959486915227686	0.2390155549778896	0.3146995511600679
0.6820661273871806	0.0857491499373424	0.7404438452659150
0.6037191854048487	0.2336476964460559	0.8165949100675363
0.1877640272805507	0.5975599765386619	0.2274905495474897
0.1282620059970796	0.7566187361732233	0.3211116304275237
0.3283312787797072	0.2412391912569589	0.5117128178158552

0.1011138735788393	0.0904745857545990	0.4885154521053607
0.8391731527378496	0.7202536358123339	0.9899614840604585
0.6154748292663612	0.5821094627514398	0.0232711654831519
0.6521766468960735	0.7835706426722595	0.4957033746685866
0.8982541283833712	0.8955810582366794	0.5108768386126954
0.1420388319661470	0.3032112921157761	0.0030946122240110
0.4027933844488061	0.4093701893282500	0.0041027960870323
0.6648942768990693	0.2206480957702084	0.5029490085845012
0.9013666681032149	0.0782448706155776	0.4875964297795363
0.1676508788653592	0.7316816759444293	0.9909042419632073
0.3846301225676285	0.5907959208622202	0.0380727252556312
0.3478968945271035	0.8194726063890769	0.4959879045097821
0.0985787530929727	0.9071379235181283	0.5134845208961423
0.8519681134986925	0.2849111903452695	0.9965788743397539
0.6017977412746021	0.3992018339210830	0.9810244535045953
0.2507528099856415	0.2563871820584822	0.8050567165211859
0.7628465254426047	0.7523727487255698	0.6987435605608141
0.7588351248178355	0.2445404860326192	0.2482848574266399
0.2648690099487752	0.7526360386317918	0.2415774294920823
0.7425673115728628	0.7398058182085663	0.2388348500199911
0.2382173664005691	0.2601385591203780	0.2458290831081200
0.2438119118275637	0.7630243034102691	0.6977684732092371
0.7412283978149077	0.2540706140115816	0.7954726988416709
0.9981011711332144	0.2094584266401327	0.5433340246237321
0.5063701242657004	0.6883985584836481	0.9439717182510350
0.0028885620063619	0.7770986971681779	0.4565644603367360
0.4987202598948315	0.2773826573164868	0.0279849072342699
0.1980857735501758	0.0036552436745865	0.6774427690070439
0.7087777248406795	0.4664841594819791	0.1638318721021648
0.8024901443620180	0.9966209962738617	0.6791074814113074
0.2889831150196898	0.4711164396373277	0.1635476312384228
0.7998948608362824	0.9829980387040536	0.3252822007328291
0.3043637986075325	0.4908711234005949	0.8125198281607595
0.1970440201299937	0.9896482933619239	0.3212759492263686
0.7014743556535379	0.4890194962327286	0.8094672318735121
0.2451748415619122	0.0286077874357531	0.0017181788735883
0.7533637191654847	0.5000476647401868	0.4890738288704668
0.7508967229413912	0.0012944131512853	0.0012363914472076
0.2513510904291110	0.5084027589332081	0.4935304296276470
0.5093407389776274	0.5994995733851454	0.6211228081563576
0.4919995453837700	0.2092804497666023	0.5749841489949006
0.4162983998715220	0.7031903122599772	0.9994464690568492
0.3203730922181549	0.2094696044476478	0.7178576904968992
0.8194867766461797	0.6965716301822980	0.1941946129084613
0.6825954133142389	0.7924736435222783	0.6963531656136226
0.1617685822248873	0.3088251668631727	0.2124553058367411
0.6836698921816264	0.1983403683227039	0.2956358793600344
0.1777560834905785	0.7075526977027806	0.7822251771997912
0.3356748385496502	0.8151082879672060	0.2831711168486422
0.8222338731027037	0.2934178695252641	0.7958991487456331
0.6695056060943827	0.7935889316375437	0.2860562499879649
0.1734869722414132	0.3054824589388971	0.8034668669822480
0.3121403286216236	0.2135154664983645	0.3058456312983822
0.8305002828269892	0.6970498013369379	0.7805615777040700
0.3228119762470811	0.8098233835947681	0.7003156285132661
0.8347629258694482	0.2929135265251404	0.2064132411341788
0.6751338686474710	0.1972800851042535	0.7127368634068034
0.1881616708427032	0.7102519987240967	0.1964148900877944
0.0818248909187389	0.2001300417908070	0.5060558804221316
0.5882766483120889	0.6896876323295871	0.9925645273902148
0.9198714822913772	0.7862556628430966	0.4915094365497552
0.4140141710880130	0.2976670419692837	0.0119789338652438
0.9164419660764835	0.1895015227979471	0.5021015601416750
0.0860830419928149	0.7952014535383043	0.4920917982608728
0.5824820167205758	0.2892192705474272	0.0020646701875009
0.2691247083908408	0.0092656748339621	0.7979104757966269
0.7782074500295835	0.4892711302905468	0.2842103470090649
0.7302112602638999	0.9951859973444712	0.7927724259194164
0.2233697779020929	0.5002654511205051	0.2897844599848369
0.7285096470193935	0.9929120015971904	0.2079902915089633
0.2308734205552625	0.5025009635792586	0.7024700722926553
0.2679976863592088	0.0092588070299655	0.2065020822374777
0.7745244662334940	0.4945268784960248	0.6976805249151354
0.1497416225656778	0.9974161580603109	0.4999987125375327
0.6575968420878056	0.4860529873627684	0.9942672166274633
0.8502275635296925	0.9880790867909042	0.5005985247622817
0.3450001502947515	0.4952757350131449	0.0055586903155032

P1(H13C4O74Al1Si35):

0.5066978588465787	0.7790524434497499	0.7056172931882330
0.5086391978090248	0.8750729131070969	0.3745126489651511
0.5346978493463510	0.9332205341993500	0.5746762060294088
0.4462551788249201	0.8913854573583109	0.5477637358142786
0.5514550951540755	0.6216468596984157	0.4292955629254962
0.4567324621022664	0.6240945783276501	0.4130207682402727
0.4988774416190438	0.6039916943750043	0.6271349836434297
0.5619544892714003	0.3716625799256477	0.4582796307929726
0.5678318471998196	0.4467156630986722	0.6513505991616597
0.6104922751180979	0.4796300343128337	0.4458539782922131
0.3927672352331868	0.4832090422826738	0.4475009032816075
0.4388460000756069	0.3735651536002678	0.4580137657735790
0.4360034683896075	0.4484710142976809	0.6523349776655252
0.5019389666253957	0.8765503417361558	0.5189489234514255
0.5021733130137775	0.5949394339512750	0.4832860896486721
0.5658466707225571	0.4436797037472360	0.5061161281389701
0.4367841438819653	0.4454563680720298	0.5071028373784685
0.3300771401784459	0.0551501670751477	0.7438195740074818
0.4066691229314330	0.2004204232408284	0.8366160472116277
0.8250984834013835	0.5615764377854262	0.2537629600377471
0.8806405500782830	0.7251521795283864	0.3369088120704333
0.6918924085365106	0.8767885946085272	0.7299764242861997
0.6445495905178262	0.7097022509266537	0.8319566218334842
0.1834359803068324	0.3826176044509637	0.2526276941825287
0.1136402745556921	0.2275010215022135	0.3321021728599973
0.6891629140371691	0.0622904248521436	0.2471142344288708
0.6316413914004428	0.2277510901585913	0.1763813734982733
0.1750884109099431	0.5556288288030231	0.7294740271962468
0.1038693868710538	0.7128081953714940	0.6767530250442988
0.3314636790838819	0.8841148125722924	0.2546316194724270
0.4004062249035414	0.7254497001589115	0.1904825710636757
0.8260459316905582	0.3780046167382736	0.7687258735313591
0.8828049778560541	0.2157577572973253	0.6785848626067548
0.6877598422612508	0.8791325016228342	0.2655844548116590
0.6154317155009323	0.7257746798508364	0.1808676863942651
0.1880653426004457	0.3734062290500546	0.7576207347697874
0.1356823193541317	0.2067697842251164	0.6807832350716652
0.3278673274413819	0.0665530717270286	0.2556278673908494
0.3871605775356954	0.2302298443674999	0.1877226652025925
0.8356174452957248	0.5609647210085740	0.7392843010808434
0.9130453780323222	0.7124767520437914	0.6808210125747749
0.3160638869555186	0.8747329770270937	0.7385756529826253
0.3798085778562523	0.7138179718872237	0.8074879376905102
0.8321748661537782	0.3788366939663135	0.2540915806562367
0.9029219949670590	0.2237978218601242	0.3278875523756213
0.6818134251933330	0.0589813875006442	0.7631316176693588
0.6099221983051351	0.2139934963081132	0.8243126680567414
0.1999602191605518	0.5646369248265231	0.2533715522837880
0.1322975489605867	0.7196277366925088	0.3299765803561030
0.3544339854301697	0.1905908273990775	0.5131918222669185
0.1046626969204638	0.0593208256207802	0.4824405412469517
0.8642391071544253	0.6780646444503375	0.0009890496727110
0.6111339023983859	0.5615657886477976	0.0205277352906563
0.6802677524271488	0.7379380336837116	0.4937603339270922
0.9011237893921020	0.8784437483082144	0.5244237446263043
0.1649878776113027	0.2494545366449898	0.0116233171358502
0.3963644535557123	0.3714371149035429	0.9611897927355102
0.6589934400925657	0.1823016370996413	0.5054847878230362
0.9090482359428762	0.0609713310828539	0.4966268215789000
0.1496399773321215	0.6709155851573243	0.9972350560628414
0.4094771985261758	0.5513020655887715	0.0141579624294366
0.3286322993369950	0.7456095285605215	0.4877054416199087
0.1144401810296287	0.8767617549823754	0.5097625041176670
0.8469593672174227	0.2466469182734556	0.0102021834390484
0.6145078762007117	0.3797756925177396	0.9787772330873139

0.2693066511953575	0.2226956392386015	0.7873535175230657
0.7772987856016655	0.7313640176304759	0.7412702621973111
0.7643956235976077	0.2176628561375722	0.2895429557333102
0.2594990682395917	0.7298883789781243	0.1834397187874899
0.7531817055845380	0.7184238702659371	0.1949071725801232
0.2519087890001259	0.2216965301488187	0.2848945769182691
0.2404122383522846	0.7206349918078629	0.7474787833319354
0.7474278895024824	0.2246014998279549	0.7687220741429073
0.0085251710050329	0.1883340641179814	0.5459811452418535
0.5139132313384991	0.6900166591707162	0.9454713098778171
0.0074917205241221	0.7660774277498206	0.4456657990662691
0.5078573623367504	0.2673081792257648	0.0407101198669793
0.2030772336958009	0.9842898709010015	0.6859095142085252
0.7086799719691612	0.4613065615827310	0.1856147755519189
0.8053713307915018	0.9851725224072965	0.6787910239629511
0.3114327550598901	0.4540801252065805	0.1904425155420384
0.8083568188308377	0.9703147989867205	0.3242670894567965
0.2971321134237712	0.4836530044089287	0.8419567938941199
0.2106558101839013	0.9717053025274822	0.3299884350839264
0.7129024580591263	0.4840724633943765	0.8309131862205987
0.2585797942578481	0.9740551714368095	0.0038485830833181
0.7587978610450037	0.4653413154440926	0.5063705628369988
0.7555629720376444	0.9629390331172232	0.0012777233150487
0.2594521409353661	0.4632365758587156	0.5087800986857332
0.5235571857249397	0.7871461476277375	0.5844111363109050
0.5017097858848203	0.4918189597031244	0.4437980105583250
0.4244390587663460	0.6714978821558830	0.9918963640588672
0.3388502719587336	0.1666293144953812	0.7198349795603534
0.8309503571183825	0.6711000437651222	0.1979057561456656
0.6967492213222926	0.7641054876691058	0.7000200423278216
0.1787767434863525	0.2704011919301337	0.2200026386761991
0.6864203463194869	0.1717931500884973	0.3026760195444993
0.1683221592871078	0.6653078360008990	0.7876894511885411
0.3317961438076082	0.7701730538470954	0.2780396886343652
0.8260417546786982	0.2659723551431412	0.8049643272118274
0.6839295151669376	0.7659433817640391	0.2853999667797140
0.1895502569719270	0.2632201952598052	0.8077737761434634
0.3296058225866858	0.1761681214202895	0.3083584351265216
0.8468556812595764	0.6707369900554951	0.7907796858232956
0.3179874193803016	0.7621428235705636	0.6990394176165822
0.8365749169098819	0.2666893142466265	0.2198330099970676
0.6756322717784968	0.1691441859731029	0.7153666456860464
0.1863120977392896	0.6720043887575713	0.1920591636300273
0.0914515501388067	0.1703632733900733	0.5098431165662447
0.5945115188731265	0.6735856700043925	0.9932962856495422
0.9256291704366078	0.7707146749956308	0.4979713025200425
0.4233788911788139	0.2671768654455917	0.0064334128165839
0.9255817844837395	0.1719325884069320	0.5119980712861434
0.0907849496666060	0.7685242945470208	0.4916037110517877
0.5914283975907324	0.2712660020357305	0.0049662730330624
0.2767329491693857	0.9700061893169050	0.7939703562182814
0.7819177537304753	0.4673540177095177	0.2989515154882127
0.7339810991213369	0.9699515536517396	0.7931749230261858
0.2380836726856259	0.4670128828106925	0.2994472313829277
0.7355389516098825	0.9684627684801015	0.2100551793074601
0.2299645642471333	0.4693385595054451	0.7113805288792818
0.2819292768517840	0.9728956686298531	0.2111749693906972
0.7839310871218998	0.4721311099499204	0.7126093906202229
0.1591969788196721	0.9723754148525074	0.5015379624936926
0.6627039150302210	0.4726269640396623	0.0039352706055809
0.8555507977377488	0.9730449168679272	0.5055211634738086
0.3539609595360318	0.4686088853970318	0.0030309493649909

P2(H11C3O74Al1Si35):

0.4986614081686227	0.6799781613980329	0.7092212185700006
0.4973486519037920	0.6364876204791017	0.3389050193268943
0.4777265857239783	0.7482947243453779	0.4283326102196199
0.4199966601232674	0.6523564935361250	0.4703150068635082
0.5449413769244060	0.5162581791344110	0.5715322085922452
0.6305937462900673	0.4629648608945658	0.5458748375165925
0.5698457382139850	0.4638273508765280	0.3607514987746185
0.5821337895173357	0.3171558451525627	0.5362816104222148
0.4629291048508719	0.2793161032208147	0.5501724599864914
0.4719941494057451	0.3573041169151594	0.3637492281693113
0.4416023610675452	0.4014070826512679	0.5750831464029247
0.4753297973981319	0.6729208025999824	0.4545274210906669
0.5753356237482876	0.4610371521668171	0.5051730685927325
0.4750736348297053	0.3503649491030387	0.5079034389520203
0.3053486534415768	0.0758317093671721	0.7398980147843019
0.3876167353221760	0.2171015492904971	0.8253311289909391
0.8086820244981254	0.5994984858201420	0.2383189212019658
0.8776990366791537	0.7490889986952070	0.3397192571184746
0.6821763444296849	0.9222869524619597	0.7917961111937331
0.6179611353358894	0.7633841106814572	0.8447153073075668
0.1762536947308817	0.3988279850620344	0.2376550781458420
0.1195314470745359	0.2350198555089946	0.3198660398080548
0.6675393548868698	0.1089442107045642	0.2779558432796989
0.6022341281253887	0.2720023626029615	0.2031680304540018
0.1608706719395911	0.5780572440697966	0.7117342912129558
0.1063692852447247	0.7479478420754617	0.6723793947000800
0.3119769402071171	0.9033139587375416	0.2602682754101551
0.3689592053159103	0.7400614498770324	0.1743309371121455
0.8279060597091998	0.4197939982877585	0.7644981068051280
0.8934832012063296	0.2597961350205082	0.6932471371914986
0.6757934960243261	0.9252731633524007	0.2428001505011181
0.6043524101772064	0.7678674572793582	0.1948381145012377
0.1655238993147066	0.3941542948887147	0.7422521896891894
0.1173249980117319	0.2238747371508012	0.6757283848356508
0.3240242547376795	0.0846576289385865	0.2148151751936140
0.3930107115951458	0.2436198658208895	0.1800875397327175
0.8183413406893436	0.6029097484789645	0.7720267389051827
0.8872553870738855	0.7590120089997825	0.6953891644362997
0.3001507185526791	0.8936762359595605	0.7374406566215299
0.3789843550942962	0.7422819608710114	0.7920634302644487
0.8247624276867995	0.4166050442374782	0.2646891132128459
0.8772704647097598	0.2503649858210650	0.3391795567078333
0.6823574573599487	0.1030903819150595	0.7336304147475090
0.6256596384034978	0.2595502872229360	0.8584279396132359
0.1863768449285743	0.5816603815009245	0.2521084527002273
0.0993178621152069	0.7208256373138724	0.3210944679331220
0.3495640345159714	0.1944672401878549	0.4953965373840816
0.0866456685643371	0.0746956315356258	0.4812250275962953
0.8286725949520246	0.7376933060475679	0.0114811201128404
0.6189378115761528	0.6074345318880958	0.0269991285724984
0.6464356770589532	0.8183590740427320	0.5157085212110388
0.8856743386276591	0.9133512528065779	0.4966593324627127
0.1629230581235532	0.2644821582991952	0.9949367234532218
0.3784412963948185	0.3876840572207030	0.9558743419979336
0.6449557471038716	0.2425705880701940	0.5188919922617714
0.8980805406380767	0.0961661937569147	0.5226159033963640
0.1329163303161636	0.6830518422027581	0.9914860102877512
0.3930567974125054	0.5700694241210442	0.9841803243559255
0.3086616725614633	0.7636017469116396	0.4890777254255383
0.1118221552197838	0.8927666031435777	0.4477896842927365
0.8369735042687623	0.2876425041526218	0.0110866637238090
0.5945435358232416	0.4245946765517346	0.0007204361296473
0.2545245935006974	0.2508714605000861	0.7305610599144998
0.7491448712447379	0.7622354787852359	0.7235690683941840
0.7414440927063239	0.2670755953340205	0.2624132072708605
0.2285239300506916	0.7588050147995631	0.2052708587409882
0.7404288443687363	0.7611021957337556	0.2791724727093836
0.2563176021506379	0.2474715106965206	0.2541325025168106
0.2392188561288862	0.7288566294878507	0.7827284906576040
0.7557594165835297	0.2623176631571411	0.7274767076355673
0.0004116657427318	0.2196576155240422	0.4870357080727672
0.4998281124849981	0.7040579076430191	0.9887932301653777
0.9949750529600863	0.8001368647939557	0.4934291932713748
0.4976211337496466	0.2927578041444932	0.9750902449390111
0.1819525456292226	0.9929701720238313	0.6857472486034055
0.6987717620980689	0.4860394419393970	0.1955730968154583
0.8025342229605528	0.0084213340958996	0.7105076283079299
0.3024603349485915	0.4782916777901178	0.1916579372983804
0.7875299493707431	0.0232052467327009	0.3575889550872660
0.2751729136739982	0.4959833782146319	0.8475255372296786

0.2046716844966170	0.0179495451293832	0.3373337981107909
0.7041387682285105	0.4976957126739094	0.8381028856096435
0.2392638005105425	0.9887955680568297	0.0020620927535333
0.7536586054399308	0.5076217344147977	0.5144926354917772
0.7451476746952252	0.0366637711260225	0.0229687724835017
0.2459237678862181	0.4788178180125797	0.5055740204764660
0.5172966206151628	0.6474538460404133	0.6051462099805462
0.5477467474755358	0.3700097915992444	0.5663859987293449
0.4081335743480210	0.6904840812653532	0.9889465521524130
0.3223244260591258	0.1837355656070514	0.6989500788799319
0.8134999454939589	0.7126137566568431	0.2175223159615527
0.6732764203879356	0.8152925404559213	0.7197253445240719
0.1786927238625893	0.2869681025799409	0.2019827123706932
0.6662501654430315	0.2206494908620584	0.3088285605928789
0.1609308068821989	0.6850448513172367	0.7882813077496209
0.3068295130335272	0.7891356633989375	0.2794384537933823
0.8294897704523549	0.3078665697364400	0.8005360013246730
0.6665112293641471	0.8170076628583729	0.3071796942573144
0.1752117995124465	0.2838780097768918	0.7858159255726704
0.3291888565447891	0.1912404656266986	0.2857473406985775
0.8207919689383019	0.7158085362182405	0.8013266289359677
0.3089237840235057	0.7811262010520466	0.7017490173004575
0.8208352803288648	0.3055210726715387	0.2193611678482199
0.6788238449807480	0.2151488331560074	0.7159208134602508
0.1632746077551346	0.6863906925185574	0.1931063060642231
0.0823560545795203	0.1881181037553574	0.4908565983767872
0.5820975397528088	0.7112236793263662	0.0138462212161201
0.9112659747022249	0.8052502695262262	0.5073207918004101
0.4119313105818918	0.2860851010311194	0.9855329901522154
0.9172127926061577	0.2063399893838067	0.5111405346216600
0.0795409266421743	0.7896398616310520	0.4842835196938680
0.5794946693096179	0.3130887831956812	0.0083614723776080
0.2569128708408712	0.9858773727261934	0.7916525549057667
0.7720457194766723	0.5039779352345946	0.3038580460320759
0.7283449177167753	0.0159331275155381	0.8150854946139177
0.2277879181439459	0.4856966611704649	0.2952116616951272
0.7196026396177118	0.0213561169713898	0.2265958016253435
0.2124869495998709	0.4875599881446320	0.7031109270157216
0.2697144837917591	0.9960358249535304	0.2032996567254983
0.7763986064652855	0.5081389149927986	0.7232009572687943
0.1468143920223000	0.9932934079588804	0.4884359807542396
0.6543282408326832	0.5065922481445497	0.0135963226453839
0.8433724593273766	0.0097135422292709	0.5206795760535243
0.3382150272928115	0.4872190388098616	0.9945935395389114

P3(H9C2O74Al1Si35):

0.5076763531600292	0.6291027856933908	0.7250622816482348
0.5009918505586697	0.6105627583980393	0.3478550916332992
0.5044443193418715	0.7174898968913439	0.4684129660345420
0.4272108295174348	0.6465290877672700	0.4779520043423702
0.4365285982632869	0.4466461270421505	0.5868293104375510
0.5125117437595037	0.4318729693313088	0.4434834955618925
0.5223555756276355	0.4199483768606243	0.6794577480944994
0.4402944276055791	0.2928791111646447	0.4561105450538037
0.4489563149940139	0.2832095931497065	0.6652747503376872
0.4851347817863569	0.6451915925846308	0.4715764109834808
0.4869252136047564	0.4114419814492152	0.5666170857049693
0.4741914467343378	0.3074698550191215	0.5519584562530895
0.5153154082834988	0.5932779343132424	0.6153648213088232
0.3253044525470585	0.0952732606822835	0.7454401616387045
0.3988219589314355	0.2502326100162051	0.8130799003989253
0.8179699377409477	0.5890814832315456	0.2332742414389415
0.8769004079795266	0.7493739926934708	0.3294457122151044
0.6837033056210444	0.9072391723938011	0.7569465508329500
0.6337735291106910	0.7390571547615536	0.8349676254115934
0.1613084007902259	0.4133534565383599	0.2670840178763925
0.1025305977337680	0.2475237860381228	0.3193937326708607
0.6858698565860735	0.0929782579870775	0.2698059424504393
0.6238796928659909	0.2522855338476901	0.1820630897649309
0.1880069424434367	0.5916658562710495	0.7606743681343886
0.1051055610622882	0.7328352881088520	0.6704950542105692
0.3140411184616170	0.9158083038527067	0.2113132688036288
0.3986583882832164	0.7691695462502324	0.1819812682701070
0.8204948046537126	0.4054338529785966	0.7500826115854480
0.8728318552025556	0.2387024406830633	0.6756969130702331
0.6797074771350253	0.9108085977770841	0.2489131854075310
0.6043005952981488	0.7590896641900713	0.1771669147593258
0.1801344548516894	0.4093001128220166	0.7411023623406265
0.1300825648057327	0.2404107195709386	0.6671198600230781
0.3039569994946518	0.0945786290910462	0.2833706130212121
0.3760740446968001	0.2419019300360148	0.1718329968277246
0.8197033659843811	0.5882631796934135	0.7614934086465652
0.8995551418964354	0.7344300320928170	0.6799679401920571
0.3152902532607484	0.9130764397736186	0.7792427790913692
0.3755986171470269	0.7453454917203715	0.8069760855845871
0.8347513980184829	0.4077125227941423	0.2719145782225709
0.8973815002697521	0.2447111869303669	0.3258850058995577
0.6761029488851520	0.0901386172197007	0.7539280601918392
0.6038459321940763	0.2443189484771793	0.8275571340216885
0.1791173480064501	0.5949279299670565	0.2351737379924046
0.1241721425836246	0.7585959445775572	0.3192371700931176
0.3404094304095082	0.2305413289156277	0.5041618755257531
0.0963537023440963	0.0860589675348962	0.4910591514333476
0.8403660800727053	0.7223539709883156	0.9968287791082417
0.6179696559521375	0.5907462591104249	0.0379801535898565
0.6532873245588107	0.7875580628622885	0.5004306088934527
0.8951089574943225	0.9014831822792999	0.5262733910162751
0.1501112880367472	0.2903611367868777	0.0012799531274581
0.3969074502275589	0.4050094848225285	0.0092542438402035
0.6605488940689170	0.2232881786852090	0.5119457357058224
0.9035841588756597	0.0838661791969386	0.5003652481664815
0.1629789016841841	0.7263864545329710	0.9909491028260362
0.3853532931882953	0.5879050423719860	0.0329452744000491
0.3431265343648704	0.8159801845889589	0.4925798341149203
0.1036894367923580	0.9039316950076284	0.5328372711133653
0.8494275382870029	0.2854579332424478	0.0084766156820777
0.5997245882147553	0.4083076653153626	0.9966860832582256
0.2594365731859156	0.2590549920101992	0.7943893746275634
0.7630284468231991	0.7568793839269858	0.7071895707499891
0.7595184640740769	0.2509699835024151	0.2714430048635634
0.2608721775497003	0.7450408671869226	0.2413786490398380
0.7415605400154206	0.7441281559381920	0.2390325690960609
0.2406369567595590	0.2610009135926532	0.2645463847821148
0.2407607980454429	0.7625205764702159	0.7031840954948976
0.7418914323323378	0.2536675082759743	0.7961604348552100
0.0006345552933382	0.2150566100457780	0.5519241002752366
0.5068645793348026	0.6928692274683002	0.9502821842704989
0.0008228786411664	0.7857077898424265	0.4628998374499034
0.4999281057936500	0.2826282561679037	0.0436523704821710
0.2053210799259872	0.0137173208440728	0.6575540460740115
0.7105658436166209	0.4715097684095255	0.1694453534541296
0.7986007115417451	0.0085260482087861	0.6794850939803183
0.2835981652579633	0.4751780065055797	0.1536302781273449
0.8028902053756539	0.9928587353948473	0.3262745264970874
0.3051630432203751	0.4885412291900550	0.8041871437506956
0.1894392834672445	0.9885693847841921	0.3074798848842661
0.6997607422862870	0.4942188626375810	0.8172047386110677
0.2427427423758459	0.0267965742289746	0.9935045292410720
0.7534282166837087	0.5008490320673360	0.4977241175245055
0.7509113889461929	0.0060448204440817	0.0051852736767728
0.2479466581222667	0.5019551407144363	0.4885223754350747
0.4164711357540583	0.7011831667644515	0.9957738138597350
0.3270208506606167	0.2060741783530275	0.7139507944154815
0.8190838827641613	0.7014707691373445	0.2011208212318820
0.6827271992694364	0.7970319634297454	0.7017576471868949
0.1631559729934793	0.3036193664145230	0.2126269986934659
0.6832847215595947	0.2043943932067123	0.3075156033876425
0.1753895127429672	0.7037276764772642	0.7825574059592029
0.3311080375395150	0.8092494356754045	0.2801320424415010
0.8214870830818626	0.2960305497808378	0.8062874482250422
0.6698651045842468	0.7999593255613391	0.2910500230382524
0.1787359223835665	0.3007331608771224	0.8002578402021143
0.3129115454700226	0.2056919848568128	0.3013796768910382
0.8302069409821513	0.7002046404175104	0.7874841578351663

0.3208595002338868	0.8062364226941199	0.6986776565879396
0.8348981703641130	0.2974461228310474	0.2188349926210265
0.6716444119420686	0.2019456578258101	0.7213590066412578
0.1827443167774661	0.7071356690617847	0.1975918632562141
0.0826758579227800	0.1973497208206680	0.5068210369786854
0.5883804702397839	0.6964935301542283	0.9994465147515541
0.9176177412684225	0.7928399393229668	0.5000909438969003
0.4164994328564404	0.2965642357602363	0.0132874773029528
0.9180600472818057	0.1954028452293670	0.5133166011785661
0.0846107509434404	0.7946061390485397	0.4965594488983456
0.5841105665860766	0.2971743514425000	0.0126682686585937
0.2715656103088762	0.0084873801419789	0.7933226543626972
0.7790502585692352	0.4935758766393903	0.2930828568931005
0.7275979045165997	0.0018369130076579	0.7981286480730319
0.2185697741120407	0.4984789736846480	0.2854346218919730
0.7300143357222311	0.0000208839234475	0.2131500682220704
0.2304833073605650	0.4993960692400066	0.6992654721809259
0.2624265066006615	0.0039786246673188	0.1991952909009953
0.7732544081946386	0.4976065514833365	0.7071262524624089
0.1492240233716728	0.9970572775289241	0.4973022484156431
0.6574059197538134	0.4932537837699797	0.0045519775402880
0.8495499819824559	0.9961364209195338	0.5079906605190203
0.3427653332866569	0.4951852850205185	0.0008864388689531

P4(H7C3O74Al1Si35):

0.5072156504714798	0.7450265366120092	0.7489970382533997
0.5443719548935562	0.8840653297929393	0.5593663240956701
0.4515411885341010	0.8745605895357542	0.6041031631583564
0.4860172750482477	0.8350575292151277	0.3944411810176476
0.3970377756853622	0.5873908504340264	0.5732902499729278
0.4809322567260352	0.6955540348272322	0.5521553608558847
0.4345860395184005	0.5944371029843651	0.3444338826660527
0.4965667909803315	0.8429818762187224	0.5363499122357993
0.4432779716048216	0.5857350315532344	0.4872150169779417
0.4948171506404719	0.5279166873783581	0.5348061914943116
0.5101097180316785	0.7496739813990061	0.6072845765052293
0.5426139515103543	0.4823514619331419	0.5795651284218124
0.3304418930533330	0.0942991565680202	0.7735669398217695
0.4014211602613145	0.2493016923915121	0.8288385531914315
0.8225221448275875	0.5984694623001729	0.2637766195219768
0.8750937201996294	0.7651012869388794	0.3411229930854489
0.6898373164080880	0.9142109738001167	0.7296855162151701
0.6390237708704021	0.7520954667379414	0.8386585569211036
0.1732522308834916	0.4212436005391993	0.2552731821822576
0.1090541068034412	0.2616312335002107	0.3300383265556803
0.6855948743537041	0.0968622013501275	0.2452183360054576
0.6253488198752777	0.2604893711719072	0.1810742925611066
0.1769491357604949	0.5967360881399628	0.7385573076300886
0.1004140364114292	0.7482888112399451	0.6754694831136092
0.3160871181067506	0.9222828140998729	0.2723244557791205
0.3976889253435019	0.7775898747084895	0.1902023485121092
0.8212844076852548	0.4135426282265229	0.7791670374362738
0.8778549676928264	0.2514061814683402	0.6863696336012666
0.6823749831659711	0.9139559215048578	0.2675698437423577
0.6081980222473575	0.7617717887276072	0.1862117530271590
0.1902136618860624	0.4157485302106565	0.7768473908824305
0.1274452283796066	0.2574073668496411	0.6827474486535081
0.3119671955670000	0.1042352916635636	0.2410028730999088
0.3804206890753790	0.2613168076120331	0.1810757619984997
0.8328600019947245	0.5957359488879845	0.7466487423213692
0.9083729541335188	0.7490508171545542	0.6850301942610955
0.3180833659356779	0.9140990552716630	0.7312332043144920
0.3748685951151103	0.7499950493550074	0.8101783479594928
0.8229223294838306	0.4156910196129004	0.2484712248347378
0.8950026558101314	0.2634257076589464	0.3332745228704113
0.6781231834062851	0.0954391367910858	0.7794260795111470
0.6048583345345335	0.2503508504292640	0.8303419806232029
0.1857020800141456	0.6039869273577949	0.2665358289455639
0.1303652073825248	0.7698392827954024	0.3302074109564899
0.3517867226459757	0.2130109685946664	0.5091876004748883
0.0987836377917333	0.1006140860748630	0.5047186580789429
0.8579388106950248	0.7159012127729767	0.0063798829766029

0.6038835238907510	0.5985485157169066	0.0157436464970644
0.6712722751768112	0.7763770520268949	0.4999747395736236
0.8976534130262621	0.9164308222829050	0.5333775570062329
0.1659822746204540	0.2844216810542832	0.0147815702290544
0.3936086122855471	0.4140856381334430	0.9846204297207350
0.6506978466348556	0.2086834410071887	0.5103202391765080
0.9043122373718617	0.0992452918239835	0.4959619069594723
0.1445934519258003	0.7119730216911009	0.0005927496245590
0.4032633187678769	0.5951267565537393	0.0400297823565978
0.3254294693908761	0.7786627565149260	0.4906935103156655
0.1077235230879978	0.9179601148011258	0.5307287671465701
0.8363092026757712	0.2779272347044710	0.0157624945094241
0.6120176256542891	0.4152864725653629	0.9909499311015111
0.2633496990278488	0.2578965577354850	0.7699642079644562
0.7724286048423679	0.7642172335713866	0.7407158735413475
0.7568311896220123	0.2543037328769273	0.3030322014751832
0.2578582470540951	0.7582991229678626	0.1849940054760566
0.7467219953105442	0.7513660830656903	0.2038163489645726
0.2482704610427575	0.2669967706609313	0.3004101464613385
0.2352552148319873	0.7667958142166356	0.7540655653687054
0.7413424139215579	0.2620332741573321	0.7606618790107547
0.0022190260426171	0.2298665016491483	0.5447660347123389
0.5089228491586084	0.7295303478386970	0.9379216186572137
0.0031070397379764	0.8036418256676328	0.4499850695151864
0.5027784647412830	0.3043503500895852	0.0448355116288042
0.2077368611943484	0.0256902602463498	0.6668069557291716
0.7023249216798462	0.5076087532851687	0.1938368927896761
0.8020838085549826	0.0247486152507861	0.6849101114935152
0.2969097179217499	0.4981926492451905	0.1773688810606302
0.8033722464033417	0.0036103905512377	0.3319210646970703
0.3030044026558372	0.5264814918954457	0.8266192452975289
0.1933482628906233	0.0106361198821716	0.3133447836763850
0.7094051468642988	0.5223407396244113	0.8395829241707915
0.2496050518308692	0.9986713896936052	0.9961695319968698
0.7561654898661914	0.5008068089640574	0.5134966816794773
0.7535038638908205	0.9940732531627461	0.0064675390334159
0.2527607652415895	0.5007383556390153	0.5059166936239450
0.4179336067954651	0.7132880818988880	0.0007187740022232
0.3359026758864587	0.2038274011082493	0.7195860067132713
0.8260624015650606	0.7074952123263003	0.2049276652708656
0.6933488653724780	0.8013328305597724	0.7036628763921371
0.1743167418857610	0.3086529521144996	0.2240049891206439
0.6796146010231610	0.2053474534594173	0.3084369102921301
0.1651738848853199	0.7057741788164620	0.7921246000083357
0.3252855469259543	0.8088594147244947	0.2828113681849231
0.8194049951844633	0.3010556376405589	0.8092620102231791
0.6778733802820369	0.8011043371326139	0.2902738998881276
0.1868100134755224	0.3037855769074298	0.8095484971888283
0.3228284320677588	0.2112523680315448	0.3064837623276020
0.8427934904897967	0.7056576432704134	0.7953843864245584
0.3140449293257461	0.8009465135072986	0.7005995904657070
0.8276213461419957	0.3029718769682717	0.2243301385420224
0.6693484844516817	0.2042749668856629	0.7201222787593053
0.1800668926141782	0.7111370118684022	0.1969088638098171
0.0855200554545874	0.2124541132276150	0.5152520815049328
0.5903346462043544	0.7103235679193958	0.9969109531875517
0.9210963163329637	0.8084848095691299	0.5031864070587773
0.4186263928543923	0.3072454654944821	0.0100132510425226
0.9193862655187885	0.2107580593644229	0.5149527988326525
0.0863294885843544	0.8095835892251131	0.4972714599086316
0.5863923416397370	0.3075704727637927	0.0112591975767700
0.2760492059913560	0.0071137147383453	0.7922087362795978
0.7759986200354874	0.5057302281661435	0.3046618287663492
0.7312279156541166	0.0071137364043494	0.7999112991592057
0.2273470036992862	0.5067009344908584	0.2999487909891556
0.7316094737765297	0.0027415456474387	0.2138178831284350
0.2308731126314569	0.5097256082923280	0.7133531312449790
0.2676997238577101	0.0087552867480341	0.2059759735916700
0.7797626573944072	0.5077129166632588	0.7202157135697149
0.1526591106919639	0.0128539921216628	0.5031979196455936
0.6575189228142406	0.5101913808349110	0.0093143502624571
0.8516514857139441	0.0106900941339774	0.5108939592861930

0.3495400010319187 0.5106442025854108 0.0083294602972472

P5(H13C4O74Al1Si35):

0.5118302822446594 0.6540605794698706 0.7069120989418565
0.5048735201026773 0.6059785710761574 0.3390071080834431
0.4873877935749604 0.7195393261382748 0.4261718881847258
0.4295466663487062 0.6250000282734476 0.4758931586492849
0.5126148233734469 0.4647834095331120 0.5769856718418040
0.5434177780611975 0.3483978548374154 0.5442624719777811
0.5015223653204828 0.4100859046098719 0.3660130592334596
0.4502090315803713 0.3720689750738586 0.5510558095866145
0.4888424301112479 0.2228902751402657 0.3964979372717607
0.4811031104431507 0.1617789787098189 0.6214750021668607
0.4279906280083381 0.9622165904077278 0.4280374144189096
0.5225453683025592 0.9525095197468616 0.4481106480493722
0.4718140058314404 0.0051485082336882 0.6271145919640873
0.4851050315778380 0.6442622070778725 0.4548193601148185
0.5018429513856475 0.3983962519102988 0.5095974402904844
0.4830964750577493 0.1597400750187035 0.4761602427502822
0.4759663363724371 0.9933345366914708 0.4839480194138019
0.3248337230098883 0.0816008516983118 0.7499565553716039
0.3991545666217391 0.2301028213375886 0.8298626489433900
0.8150478524114604 0.5977513740526632 0.2272638381765688
0.8727145612191194 0.7579168148750952 0.3278831555142858
0.6827052952304200 0.9147836699757050 0.7496733792447330
0.6358344110453515 0.7474404104156847 0.8384055602913065
0.1696032153461715 0.4135165269642727 0.2483889291931263
0.1071614983652012 0.2532299581357336 0.3326758533646981
0.6838541228738180 0.1007827734259479 0.2607198893129095
0.6236573310349911 0.2609982397456250 0.1761330810826180
0.1695704878502653 0.5837186324031904 0.7242077478718727
0.0974627731014408 0.7396202487127965 0.6718731142835566
0.3196213154703074 0.9181805366701141 0.2645863503344827
0.3924466617436835 0.7688039214463913 0.1701914694557374
0.8202638945034550 0.4145416858745108 0.7498963011068724
0.8742759210218836 0.2485062277564225 0.6745341315412503
0.6751706979267382 0.9191254379109708 0.2498407076400895
0.6012201980181118 0.7668552862822011 0.1769080108503616
0.1811827579348702 0.4021496396534090 0.7665001921111667
0.1257708840450888 0.2373062552948468 0.6862881751411010
0.3103700225111368 0.0986336381317443 0.2396463794535038
0.3778373999515239 0.2555301319890901 0.1790954740843773
0.8218803271158421 0.5973105100417868 0.7551854134431863
0.8999076250614024 0.7455756333373174 0.6771252301159834
0.3133416486817993 0.9011450935366909 0.7300188715879159
0.3735256019781872 0.7368807002300954 0.7963523604595579
0.8322084546520259 0.4165936418953251 0.2657336659401395
0.8969158319682577 0.2556243671018592 0.3236472399330310
0.6774774428664827 0.0967206055584171 0.7509630530998663
0.6034849568809477 0.2481970921553511 0.8248069031939878
0.1946954149010693 0.5953775044533387 0.2543428685776732
0.1236624608610342 0.7493287740682320 0.3230149170425136
0.3535574715554688 0.2062686696561471 0.5054578765269113
0.0953584296790666 0.0878815160238418 0.4918602549433047
0.8389935723309847 0.7316215009932066 0.9925423993674869
0.6142868800336956 0.5982826495592661 0.0350340057801333
0.6514760434328011 0.7941887859123682 0.4987152438733347
0.8967223874845445 0.9109653938425168 0.5173910453437855
0.1660379414390434 0.2723276137141042 0.0158103838811670
0.3904239997420973 0.4012079545032066 0.9611764272336671
0.6559851549132745 0.2259560889181529 0.5067388308415914
0.8999487694502122 0.0936118433268192 0.4932115685008540
0.1439111764571948 0.6982851792646301 0.9917923243944529
0.3958899583940294 0.5829578915417670 0.0314595220741296
0.3231383130781538 0.7733202020428109 0.4780429753465469
0.1043975569044520 0.9045581564239740 0.5091183960686365
0.8456052785050048 0.2915290395834660 0.0064567657548338
0.6007523175470837 0.4151457535213581 0.9855325898573213
0.2614233284517127 0.2499086016290022 0.7651792828472708
0.7631706196202046 0.7647728641555531 0.6997590163579872
0.7588174294601089 0.2581941663858842 0.2767584448628426
0.2509343846177501 0.7624439070121483 0.1783144482861871
0.7389139179282438 0.7533592763053960 0.2311785736415359
0.2455558510329681 0.2606436208330436 0.3071935164401367
0.2337455625729348 0.7504653666396379 0.7391350926333047
0.7416538156692525 0.2623489077649594 0.7819419347183754
0.0004273416844995 0.2191517960878855 0.5460573986148276
0.5062755508615808 0.7073378949608013 0.9415360522738894
0.9988028107975282 0.7907457315269539 0.4465923167546819
0.4997036408370974 0.2903829647184466 0.0402140444269179
0.2003562349382548 0.0100904037034795 0.6671542425570394
0.7071489490383911 0.4806843195181401 0.1702994325123797
0.8000796288990699 0.0132031747576775 0.6815997737939696
0.2985449721270541 0.4738002295461925 0.1798199196253876
0.7991593374824433 0.9985880551812087 0.3281549692874179
0.2919581996840677 0.5117136299628129 0.8336895337301371
0.1941402453530401 0.0010373974963684 0.3113298392599120
0.7012166933915935 0.5071792988091488 0.8187873499539435
0.2486367956425184 0.9944411609682717 0.9925170525349571
0.7533110563183101 0.5094812374960114 0.4964944827026585
0.7497076327641850 0.0092995674282292 0.0041044980372504
0.2494143084494524 0.4856642600494752 0.5040640796761053
0.5285070299906209 0.6196523998526260 0.6023050797687546
0.4839098896920646 0.0834056674091812 0.3918001956526496
0.4160471357858526 0.6994983837032009 0.9884023233173806
0.3317412038571277 0.1926939659867699 0.7153190455515812
0.8165070855912901 0.7100153603823642 0.1957990140575916
0.6826424228561123 0.8037337933612250 0.6986372974056891
0.1714493941057000 0.3007865155368918 0.2241691871952654
0.6818522270905092 0.2120809751215020 0.3025433382106399
0.1625367317516933 0.6931288335024846 0.7820446229100071
0.3234496224948415 0.8025755585532224 0.2706034189871218
0.8210495318645954 0.3047185565994326 0.8020341749057280
0.6673454249274826 0.8071931579639724 0.2889784747883013
0.1829083270723189 0.2914377772885999 0.8078498054179448
0.3197619145229922 0.2057688689814867 0.3034394411947758
0.8306415360620250 0.7093017967502320 0.7826412883035161
0.3126542915524411 0.7876285168866204 0.6900376178811172
0.8331759405642671 0.3059663915271926 0.2171408634337695
0.6713152916998695 0.2084392998075941 0.7164952849747337
0.1790716752759423 0.7013913617025480 0.1882444382970618
0.0831528758191311 0.1995031980029935 0.5132948056958355
0.5870583398459601 0.7048265591668326 0.9968624390281207
0.9166082047736239 0.8012213307411784 0.4931775818264654
0.4157482776709926 0.2968687767385916 0.0023150716497398
0.9174815255391024 0.2042070292614184 0.5092764071337683
0.0822843702218279 0.7954523995463205 0.4881739934601725
0.5830340005118728 0.3053376111536394 0.0057525148407009
0.2709935538771759 0.9940056294130315 0.7851843116418209
0.7765503050308951 0.5023635600529914 0.2901895322812393
0.7280677526510715 0.0075506197700577 0.7959730912051839
0.2285015586394579 0.4940769904599591 0.2956807822110008
0.7274907718370969 0.0063056504328216 0.2113181035845599
0.2237757176132220 0.4964391568856428 0.7082719210912742
0.2676029297258609 0.0013881538085769 0.2018289929268278
0.7737273172384391 0.5073313843252265 0.7055645419880676
0.1491189106978936 0.9997870054019700 0.4947415766916592
0.6557935657768539 0.5021405547768438 0.0012265175747928
0.8486642313322719 0.0035915178768988 0.5049392795082497
0.3452569789277078 0.4966878346665453 0.0024637235429452

P6 (H11C3O74Al1Si35):

0.5148779035936997 0.6315935483643500 0.7232442169472861
0.5109805213130301 0.6129266749103763 0.3463062493249254
0.5069913407000470 0.7209602890785902 0.4641120419685905
0.4361150088704520 0.6398287373115608 0.4800254523252718
0.4739101213251615 0.4615413742271812 0.5750214091915140
0.4510159658074144 0.4301603086540311 0.3510021296828825
0.3835463850979082 0.4606788634428298 0.5047478269548407
0.5433708344141408 0.2155122411885273 0.6916646667452326
0.4261444697575030 0.3534567113765164 0.5317072073552112
0.4754210449238698 0.2437134380291184 0.3509535688540950
0.5446287842582791 0.3181061730421604 0.4719386750254202

0.4937695247467673	0.6462777298648348	0.4699526437791235
0.4349681519704731	0.4266060033115906	0.4901383773047243
0.5090319750443157	0.2580664654895557	0.4660037476675285
0.3254669973645505	0.0833421703748982	0.7424329059750505
0.3957465266273203	0.2371358504641705	0.8207727327177068
0.8177443653153347	0.5883040497088246	0.2200393837623054
0.8767347818934326	0.7465859122444982	0.3274522042924985
0.6840191045502095	0.9100912498921687	0.7563612957400565
0.6345448049258096	0.7421976344062315	0.8359655841926070
0.1632383878896064	0.4064754127609334	0.2656667559877803
0.1066405575555720	0.2398978415752708	0.3192125693728443
0.6876185542878801	0.0911191357156795	0.2713898029145838
0.6239175876894620	0.2468220669546284	0.1718403041245438
0.1825123453624116	0.5806374373889795	0.7621782868642342
0.1048420001527788	0.7256376234121262	0.6654995509914343
0.3126486658043532	0.9084409121924111	0.2098171988716899
0.3985894585481802	0.7628331553032552	0.1767335230355869
0.8250280679160298	0.4085339021538132	0.7362604437058025
0.8720610600078089	0.2378451114576308	0.6642159230337015
0.6787543878454159	0.9091587802641143	0.2361842350397367
0.6044756828987374	0.7550164202949929	0.1773475040746604
0.1809120366359735	0.3984128893414223	0.7335263711658371
0.1280279253891976	0.2306349452482479	0.6703145982006191
0.3048543117561309	0.0880887257815814	0.2716880636770540
0.3815888162409564	0.2347252550466479	0.1780944300776497
0.8184747931354579	0.5911140572805706	0.7594194361646274
0.8992825100988924	0.7360194074847328	0.6786927829849532
0.3106279714692803	0.9016293779594733	0.7746976851942264
0.3764237489123019	0.7377596995752782	0.8016907723453031
0.8351087677487996	0.4073808354696666	0.2686239748217361
0.8979962232274588	0.2438751723760646	0.3162882186818834
0.6838091595833262	0.0931069687358104	0.7431883533758139
0.6051214723891434	0.2414770913522730	0.8130362306148768
0.1776855853675556	0.5880116557399830	0.2315392006524277
0.1249832444040280	0.7529614261781177	0.3140404799529861
0.3384681115494601	0.2189973431845900	0.5049449364303129
0.0933488534256242	0.0784604507339566	0.4879431794146356
0.8378253039998285	0.7264209637377874	0.9936790428779148
0.6205104362424905	0.5892536931928873	0.0275030855280605
0.6519182253827793	0.7926311613314425	0.4990271000368267
0.8999109024063259	0.9004263848583136	0.5160705441068885
0.1500226650653147	0.2843513188654825	0.9998990091791029
0.3952620414395156	0.3968237737134146	0.0056667158762735
0.6663476307180431	0.2271489199317429	0.5027171565071527
0.9024714943597871	0.0832729200520959	0.4894831574149734
0.1623839290460225	0.7196129494127348	0.9859132535305690
0.3886857649114432	0.5798868878038590	0.0267565823628715
0.3425841517398993	0.8067880665239855	0.4877929337293310
0.1032310890307002	0.8971471675603908	0.5284680182908303
0.8544420662964782	0.2903825436337115	0.9979963604751632
0.6020807766492169	0.4048687852255028	0.9887508215188276
0.2580468898937198	0.2459412322514964	0.7927809213091166
0.7626628047945019	0.7597544650983750	0.7008293316049716
0.7610315369457510	0.2498567211571512	0.2479005589430727
0.2612754115312441	0.7363502659622796	0.2339020307907376
0.7413498333816335	0.7429132473448234	0.2441558328607201
0.2445973142118874	0.2561138887936281	0.2542443516038730
0.2415216673818321	0.7468090980817905	0.6958749148920944
0.7437649479662340	0.2612412134529833	0.7958383275372611
0.0008643130545209	0.2125153044994548	0.5424924734639518
0.5078477350663348	0.6901293716862682	0.9475965347600166
0.0015861163187552	0.7778176468564908	0.4566658846466254
0.5008764402695931	0.2777406573179491	0.0298403911541811
0.2034359836927067	0.0079454501182141	0.6543889670486820
0.7108666686673502	0.4689276103821598	0.1643793826388560
0.8033899313013961	0.0027977947505278	0.6801047312346995
0.2854572679012222	0.4727753597600994	0.1566125740484026
0.8020368234397424	0.9867261376918322	0.3265739321554335
0.3026894951608625	0.4841457372142415	0.8065269258058407
0.1887966709779079	0.9842502749452890	0.3045565055030153
0.7021531974533417	0.4893928028212002	0.8109675508199601
0.2405466217313972	0.0169745327843742	0.9890754208584482

0.7546292256624980	0.5037595736059188	0.4907901585966528
0.7526464983984624	0.0087575659589731	0.0033262088455288
0.2468758720795634	0.4987271203661265	0.4889923109876548
0.5291737262517415	0.6001478177868549	0.6129600252770615
0.5081505166966949	0.1987309099241585	0.5894614422758906
0.4168292665584588	0.6943944852801920	0.9914084522043112
0.3275012011696248	0.1950983608224988	0.7140106821111587
0.8184459989327948	0.7014659250011874	0.1976057313061617
0.6822337532494345	0.7996324745998393	0.6997336958122702
0.1661915594632575	0.2969538768473043	0.2098374983037488
0.6864386139419807	0.2032319957889541	0.2959050031113577
0.1742037284335183	0.6936021121133606	0.7786383912152672
0.3306454398342638	0.8017466713352803	0.2752188871632697
0.8253749592364699	0.3001189707194101	0.7970670221412348
0.6691325962487085	0.7991128927011744	0.2890470858458158
0.1790857945156645	0.2907166875640073	0.7985423036318124
0.3155603284663542	0.1987550012527279	0.2998420399300628
0.8292962135797382	0.7031121008804249	0.7842597147106929
0.3198937694272601	0.7957775513542131	0.6933231720470516
0.8372933615483973	0.2985270320679518	0.2078445571067604
0.6785765391169321	0.2041826175939363	0.7122351300827674
0.1826645989903994	0.6998375050477037	0.1921193647980175
0.0833131671807266	0.1904858572465997	0.5039883366120606
0.5890487755130067	0.6949677321906407	0.9961498707604761
0.9192138252972910	0.7902000793678496	0.4952546253695687
0.4150604469102888	0.2884716556336997	0.0072993137882662
0.9187089875605849	0.1941691212035650	0.5034277065162200
0.0854426701856141	0.7877527793446575	0.4916763084556379
0.5839427010280929	0.2954121655102711	0.0044604563863970
0.2699046367038846	0.9994297731850921	0.7893317310365191
0.7794319010946680	0.4936744244238298	0.2859838919994573
0.7315752111890532	0.0014558468040278	0.7952130403287825
0.2190349068065274	0.4931430813421471	0.2848362680202143
0.7306025146369421	0.9974665760581587	0.2099289741537760
0.2286450937507780	0.4914906295377932	0.6989305123755258
0.2618122994039780	0.9975012990746137	0.1946340077915210
0.7749059448492659	0.4992169353677554	0.6997660804131129
0.1480976657471231	0.9910836112463741	0.4934950671339138
0.6589405982513554	0.4911902427963923	0.9968334605433995
0.8517992730800614	0.9926801194982531	0.5029261551160786
0.3432887827462920	0.4888292196629465	0.9991672620045762
